# Maximum Entropy (Most Likely) Double Helical and Double Logarithmic Spiral Trajectories in Space-Time

**DOI:** 10.1038/s41598-019-46765-w

**Published:** 2019-07-25

**Authors:** M. C. Parker, C. Jeynes

**Affiliations:** 1 School of Computer Sciences & Electronic Engineering, University of Essex, Colchester, UK; 2 University of Surrey Ion Beam Centre, Guildford, UK

**Keywords:** Thermodynamics, Dark energy and dark matter, Information theory and computation, DNA

## Abstract

The ubiquity of double helical and logarithmic spirals in nature is well observed, but no explanation is ever offered for their prevalence. DNA and the Milky Way galaxy are examples of such structures, whose geometric entropy we study using an information-theoretic (Shannon entropy) complex-vector analysis to calculate, respectively, the Gibbs free energy difference between B-DNA and P-DNA, and the galactic virial mass. Both of these analytic calculations (without any free parameters) are consistent with observation to within the experimental uncertainties. We define conjugate hyperbolic space and entropic momentum co-ordinates to describe these spiral structures in Minkowski space-time, enabling a consistent and holographic Hamiltonian-Lagrangian system that is completely isomorphic and complementary to that of conventional kinematics. Such double spirals therefore obey a maximum-entropy path-integral variational calculus (“the principle of least exertion”, entirely comparable to the principle of least action), thereby making them the most likely geometry (also with maximal structural stability) to be adopted by any such system in space-time. These simple analytical calculations are quantitative examples of the application of the Second Law of Thermodynamics as expressed in geometric entropy terms. They are underpinned by a comprehensive entropic action (“exertion”) principle based upon Boltzmann’s constant as the quantum of exertion.

## Introduction

In 1823 Olbers formulated the problem of the dark night sky as a paradox of cosmological geometry^1^, but its overriding significance to us as living beings is its functioning as an *entropy engine*^2^. Landauer’s seminal work^3^ (following Shannon^4^ and Brillouin^5^ teaches us that *information* has calculable *entropy* and obeys physical laws, while the introduction by Jaynes^6^ of maximum entropy (MaxEnt) as the basis of the rules of thermodynamics (for example, the determination of the partition function) is now recognised as far-reaching. The associated variational approach to entropy production first described by Onsager^7^ also provides critical insights into issues of thermodynamic reciprocity and symmetry in systems far from equilibrium.

Today, the entropic treatment of information is standard in the analysis of the efficiency of communications networks in the presence of noise^8^, and it has become clear that information and its transfer are associated with discontinuities^9^, implying non-adiabatic (entropy changing) conditions. Indeed, Brillouin considered information (negative entropy, or *negentropy*^5^) to be anti-correlated with entropy, and Bennett^10^ showed elegantly how information *erasure* has an entropy cost: note that perfect information *copying* is excluded by the “*no-cloning theorem*”^11^. Applying *Landauer*’*s Principle*^3^ to a computation involves the *transfer* of information and therefore also results in a rise in entropy^12^.

Parker & Walker^9^ have also shown that holomorphic functions (which allow analytical continuation, with the sum of the Cauchy residues being zero) by themselves cannot transmit information. Instead, network stability^13^ considerations require a filter function’s denominator be a Hurwitz polynomial^14^, resulting in the necessity for meromorphic functions (that is, complex functions analytical everywhere except at isolated poles, with a non-zero sum of Cauchy residues) being used to represent information transfer.

We will show that certain geometrical structures with simple analytical representations – the double helix and the double logarithmic spiral – can be treated formally as holomorphic; and further, we calculate their geometric entropy with Lagrangian methods (based on a calculus of spatial gradients) showing that the appropriate Euler-Lagrange equations are satisfied, that is, they are maximum entropy structures. Then, to verify the formalism developed, we will calculate certain observable quantities conforming to the Hamiltonian and Lagrangian equations of state and show consistency with real observations.

## Holomorphic Info-Entropy

The simplest meromorphic function is functionally equivalent to an isolated singularity, that we place in a Minkowski space-time, described by basis vectors (*γ*_*μ*_, *μ* ∈ {0, 1, 2, 3}) which obey a Clifford algebra that formally distinguishes the special behaviour of the time axis *γ*_0_, being characterized by a real time axis and imaginary space axes (see Penrose, ch.18^15^; we follow Penrose’s choice of metric). An information vector *h* can be defined in Minkowski 4-space, and can be shown to be obtained from the sum of the temporal residues *h*_*n*_ associated with each spatial basis vector *γ*_*n*_, *n* ∈ {1, 2, 3}, (see Appendix A Eq. A.4 in Supplementary Information) given by:

1a $$h={k}_{B}\,{\rm{l}}{\rm{n}}\,({x}_{n}){\sigma }^{n}\,n\in \{1,2,3\};\,{\rm{E}}{\rm{i}}{\rm{n}}{\rm{s}}{\rm{t}}{\rm{e}}{\rm{i}}{\rm{n}}\,{\rm{s}}{\rm{u}}{\rm{m}}{\rm{m}}{\rm{a}}{\rm{t}}{\rm{i}}{\rm{o}}{\rm{n}}\,{\rm{c}}{\rm{o}}{\rm{n}}{\rm{v}}{\rm{e}}{\rm{n}}{\rm{t}}{\rm{i}}{\rm{o}}{\rm{n}}$$

Note that we use Einstein’s summation convention in Eq. 1a (and subsequently, where stated) using tensor index notation where the lower index indicates the row and the upper the column. The bivectors *σ*_*n*_ ≡ *γ*_*n*_ *γ*_0_ also represent unit vectors along the co-ordinate axes of the 3-dimensional space, forming a quaternion sub-algebra isomorphic to the Pauli spin vectors with the associated pseudoscalar *I* = *σ*_1_*σ*_2_*σ*_3_, where *I*^2^ = −1. Mathematically, this has transformed our starting Euclidean geometry into what will turn out to be a much more useful hyperbolic geometry. Penrose (§2.4)^15^ emphasises that such a logarithmic representation is characteristic of hyperbolic geometry, and we see here its intimate relationship with entropic quantities.

We choose to define the entropy *s* as the Hodge-dual *h of the information since this definition can be shown to have the correct properties; note that Penrose (§19.2^15^) points out that Maxwell’s equations are *self-dual* in the *orthogonal complement* sense of the Hodge-dual operation, with *σ*^*m*^ = *σ_*n*_ = *Iσ*^*n*^:

1b $$s={k}_{B}\,\mathrm{ln}\,({x}_{m})I{\sigma }^{m}\,m\in \{1,2,3\};\,{\rm{summation}}\,{\rm{convention}}$$

Thus we amplify Brillouin’s assertion of the close relation of information with entropy by treating entropy mathematically as an *orthogonal complement* of information.

We choose entropic structures exhibiting a transverse helical geometry, that is, *s*_3_ = *h*_3_ = 0, with a “trajectory” axis (plane waves travelling) in the *γ*_3_ direction. Then, given that *s* and *h* are conjugate (that is, the orthogonal complements of each other), the entropy eigenvector can be written as (for the right-handed chirality; see Appendix A Eq. A.6b in Supplementary Information)

2a $$s={k}_{B}(i\,\mathrm{ln}({x}_{1})I{\sigma }^{1}-\,\mathrm{ln}({x}_{2})I{\sigma }^{2})$$

and its (conjugate) information term similarly written as (Eqs A.7b and A.9c)

2b $$h={k}_{B}(\mathrm{ln}({x}_{1}){\sigma }^{2}-i\,\mathrm{ln}({x}_{2}){\sigma }^{1})$$

Note that Eqs. 1 treat the generalised singularity of an isolated pole, whereas Eq. 2 constrain this singularity into a geometry isomorphic with the double-helix implied by Maxwell’s equations.

Courant & Hilbert^16^ point out that the Maxwell equations are a hyperbolic version of the Cauchy-Riemann equations, and Salingaros points out that the vacuum electromagnetic (EM) field is holomorphic^17^. To form a holo-morphic info-entropy function we combine together the expressions in Eqs. 2 for information and entropy in the same way (and for the same reason) that is done in the Riemann-Silberstein^18,19^ complex-vector (holomorphic) description of the EM field:

3a $$\underline{F}=(\underline{E}+ic\underline{B}){\gamma }_{0}$$

where *E* and *B* are the 1-vector electric and magnetic fields; *F* is a bivector (see Penrose^15^ §19.2), hence the need for *γ*_0_. The equivalent complex-vector for the bivector info-entropy case is:

3b $$f=s+Ih,$$

so that we have, from Eq. 2 (see Appendix A, Eq. A.10b in Supplementary Information):

4 $$f={k}_{B}\,\mathrm{ln}({x}_{1}/{x}_{2})I[i{\sigma }_{1}+{\sigma }_{2}]$$

Note that the argument of the logarithm is now dimensionless, as is conventional. Note also that meromorphic functions are only piecewise holomorphic, so they can transmit information.

Just as Maxwell’s equations have a complementary (dual, in a strong sense) helical structure of the electric and magnetic fields, we continue to choose a similar double-helical structure to the info-entropic geometry, such that the loci of the *x*_1_ and *x*_2_ co-ordinates of the info-entropic trajectory are related to each other by a pair of coupled differential equations:

5a $${x^{\prime} }_{1}=-\,{\kappa }_{0}{x}_{2}$$

5b $${x^{\prime} }_{2}={\kappa }_{0}{x}_{1}$$

where the coupling parameter is given by *κ*_0_ ≡ 2π/*λ*_0_ with *λ*_0_ being the helical pitch along the *γ*_3_-axis (that is, the *x*_3_ direction) and the prime indicating the differential with respect to *x*_3_ (the trajectory axis) *x*_*n*_′ ≡ d*x*_*n*_/d*x*_3_ as usual.

In the entropic domain the *x*_3_ co-ordinate plays a role analogous to that normally played by time *t* in conventional kinematics: to amplify this point, note that *x*_0_ ≡ *ct* and *x*_3_ are also commensurate conjugates in the Pauli algebra (see Eq. A.6a in Appendix A in Supplementary Information). Considering only the functional part of the complex-vector, Eq. 4 allows us to write the ‘local’ geometric entropy for a double-helical structure as (Eq. A.12):

6 $$s={k}_{B}\,\mathrm{ln}(\frac{{x^{\prime} }_{n}}{{\kappa }_{0}{x}^{n}})\equiv {k}_{B}\,\mathrm{ln}\,W\,n\in \{1,2\};\,{\rm{summation}}\,{\rm{convention}}$$

which is functionally equivalent to Boltzmann’s equation for entropy; where the quantity *W*_*n*_ ≡ *x*_*n*_′/*κ*_0_ *x*_*n*_ therefore represents the number of states available for the *n*^th^ plane wave.

We now consider the case of the double helix in more detail, and in particular as exhibited by the structure of DNA (which is naturally right-handed). Without loss of generality, we define the locus in space *l*_1_ of the first information-bearing helix of DNA with its axis aligned to the *γ*_3_ direction:

7a $${l}_{1}({x}_{3})={\gamma }_{1}{R}_{0}\,\cos \,{\kappa }_{0}{x}_{3}+{\gamma }_{2}{R}_{0}\,\sin \,{\kappa }_{0}{x}_{3}$$

where *R*_0_, *κ*_0_ and *x*_3_ represent respectively the radius, pitch, and axial co-ordinate of the helix. The second helix *l*_2_, with its complementary base-pairing and anti-parallel (C2 space group) symmetry contains the same entropic information content as *l*_1_, but π/2 phase-shifted and propagating in the opposite (i.e. negative) *γ*_3_ direction:

7b $${l}_{2}({x}_{3})={\gamma }_{1}{R}_{0}\,\sin \,{\kappa }_{0}{x}_{3}-{\gamma }_{2}{R}_{0}\,\cos \,{\kappa }_{0}{x}_{3}$$

These expressions are mathematically equivalent to those for the electric and magnetic fields of an EM wave, with *l*_1_ and *l*_2_ being complementary. Equivalent to Eqs. 3, we now express the double-helix as the complex-vector ∑ = *l*_1_ + *il*_2_ to describe a single holomorphic trajectory in Euclidean coordinates with spatial basis vectors *γ*_*n*_ (*n* ∈ {1, 2}):

8 $${\rm{\Sigma }}={\gamma }_{1}{R}_{0}{e}^{i{\kappa }_{0}{x}_{3}}-{\gamma }_{2}i{R}_{0}{e}^{i{\kappa }_{0}{x}_{3}}$$

We therefore see in Eq. 8 the functionals represented by *x*_1_ = *R*_0_ exp(*iκ*_0_*x*_3_) and *x*_2_ = −*iR*_0_ exp(*iκ*_0_*x*_3_), from Eq. (5), where the phase and sign difference between *x*_1_ and *x*_2_ are typical for a pair of coupled mode equations, and which together form a holomorphic function (see Appendix B Eq. B.1 in Supplementary Information).

## Hyperbolic Geometry & Entropic Momentum

We now exploit Penrose’s assertion (§2.7 p.48^15^) that there is a “*hyperbolic* overall geometry of the spatial universe … the space of *velocities* … is certainly a three-dimensional hyperbolic geometry” (his italics; this assertion is underpinned by extensive observations of the cosmic microwave background). So we define for our helix the “hyperbolic position” vectors *q*_*n*_ in the simplest possible way that involves the logarithm characteristic of the hyperbolic geometry (see Eqs. 1), where the logarithm is kept dimensionless by the normalising (Euclidean) metric *R*_*n*_ (see Appendix B, Eq. B.2 in Supplementary Information):

9a $${\rm{hyperbolic}}\,{\rm{position}}:\,{q}_{n}\equiv {R}_{n}\,\mathrm{ln}({x}_{n}/{R}_{n})\,n\in \{1,2\}$$

For small geometry (*x*_*n*_ ≪ *R*_*n*_) and for *x*_*n*_ having its origin at *R*_*n*_ such that *x*_*n*_ tends to *R*_*n*_ + *x*_*n*_ (that is, where *x*_*n*_ is localised in the vicinity of *R*_*n*_) the hyperbolic geometry is approximately Euclidean, *q*_*n*_ ≈ *x*_*n*_, and also independent of the metric *R*_*n*_. For the double helix geometry we take *R*_*n*_ = *R*_0_ for *n* ∈ {1, 2}.

The conjugate quantity for position *q* is the momentum *p*, so that moving towards a Lagrangian formalism, we therefore also define the “entropic momentum” *p*_*n*_ vectors in terms of an “entropic mass” *m*_*S*_ and the velocity *q*_*n*_′, where as before *q*_*n*_′ ≡ d*q*_*n*_/d*x*_3_. Note that *q*_*n*_′ is dimensionless, so that either *q*_*n*_′ or its inverse 1/*q*_*n*_′ can be used as a “velocity” (this ambiguity is a feature of hyperbolic velocities). It turns out that the inverse definition is more fruitful (see Appendix B, Eq. B.6):

9b $${\rm{entropic}}\,{\rm{momentum}}:\,{p}_{n}\equiv {m}_{S}/{q^{\prime} }_{n}\,n\in \{1,2\}$$

where the entropic mass *m*_*S*_ is defined as:

9c $${\rm{entropic}}\,{\rm{mass}}:\,{m}_{S}\equiv i{\kappa }_{0}{k}_{B}$$

and the Boltzmann constant *k*_*B*_ is introduced on dimensional grounds as the entropic analogue to Planck’s constant in kinematics. We use the subscript ‘*S*’ as a reminder that a quantity is entropic. Clearly *iκ*_0_*k*_*B*_ is a geometric quantity intrinsically based upon the pitch of the double helix. Simple calculus on Eq. 9a allows us to create the useful auxiliary identity *q*_*n*_′ = *R*_*n*_ · *x*_*n*_′/*x*_*n*_, again highlighting the intimate relationship between Eqs 6 and 9; we will show elsewhere^20^ how Liouville’s theorem allows the conjugate variables *p* and *q* to be used to calculate the entropy of the geometry.

We will use Eqs. 9 as the basis for a set of Hamiltonian and Lagrangian equations. We consider first the entropic equivalent to kinetic energy, i.e. ‘kinetic entropy’ (KE) *T*_*S*_, based upon the conventional definition of kinetic energy (Appendix B, Eq. B.8b in Supplementary Information):

10a $${T}_{S}(q^{\prime} )=-\,\int p{\rm{d}}q^{\prime} =-\,{m}_{S}\,\mathrm{ln}q^{\prime} $$

where the additional negative sign accounts for the inverse velocity. For the three spatial directions, we therefore have:

10b $${T}_{S}=\sum _{n}-{m}_{S}\,{\rm{l}}{\rm{n}}\,{q^{\prime} }_{n}=-\,{1/2}\,{m}_{S}\,{\rm{l}}{\rm{n}}({q^{\prime} }_{n}{q^{\prime} }^{n})\,{\rm{s}}{\rm{u}}{\rm{m}}{\rm{m}}{\rm{a}}{\rm{t}}{\rm{i}}{\rm{o}}{\rm{n}}\,{\rm{c}}{\rm{o}}{\rm{n}}{\rm{v}}{\rm{e}}{\rm{n}}{\rm{t}}{\rm{i}}{\rm{o}}{\rm{n}},n\in \{1,2,3\}$$

We also define an entropic potential field *V*_*S*_(*q*) as a function of hyperbolic position *q* (the ‘potential entropy’). However, for the present case of a double helix, Eq. 8 clearly represents a pair of plane waves travelling in space; which is analogous to the kinematic “free-particle” situation, such that there is therefore no associated entropic potential field, *V*_*S*_ = 0. The entropic Hamiltonian *H*_*S*_(*q*(*x*_3_), *p*(*x*_3_), *x*_3_) is defined as usual as *H*_*S*_ = *T*_*S*_ + *V*_*S*_, and (as shown in Appendix B, see Supplementary Information) is also a conserved quantity in hyperbolic space.

Using the canonical Legendre transformation, the entropic Lagrangian is given by (Eq. B.14):

11 $$\begin{array}{ccc}{L}_{S} & = & {q^{\prime} }_{n}\,{p}^{n}-{H}_{S}\,{\rm{s}}{\rm{u}}{\rm{m}}{\rm{m}}{\rm{a}}{\rm{t}}{\rm{i}}{\rm{o}}{\rm{n}}\,{\rm{c}}{\rm{o}}{\rm{n}}{\rm{v}}{\rm{e}}{\rm{n}}{\rm{t}}{\rm{i}}{\rm{o}}{\rm{n}},\,n\in \{1,2,3\}\\  & = & 3{m}_{S}-{H}_{S}\end{array}$$

such that the required canonical equations of state are obeyed: ∂*L*_*S*_/∂*x*_3_ = −∂*H*_*S*_/∂*x*_3_, as well as ${p^{\prime} }_{n}={\rm{\partial }}{L}_{S}/{\rm{\partial }}{q}_{n}$ and ${q{\rm{^{\prime} }}}_{n}=-\,{\rm{\partial }}{L}_{S}/{\rm{\partial }}{p}_{n}$ (see Appendix B, Eqs B.15 & B.16).

## Double Helix Geometry: Photons & DNA

### *Exertion*

In analogy to the action integral (with units of J·s) we now define the *exertion X* (units of J/K) as the integration of the entropic Lagrangian *L*_*S*_ along the spiralling double-helical trajectory:

12 $$X=\int {L}_{S}{\rm{d}}l=\sqrt{1+{\kappa }_{0}^{2}{R}_{0}^{2}}\int {L}_{S}(q,q^{\prime} ,{x}_{3}){\rm{d}}{x}_{3}$$

where we note the Pythagoras relationship ${\rm{d}}l/{\rm{d}}{x}_{3}=\sqrt{1+{\kappa }_{0}^{2}{R}_{0}^{2}}\equiv \chi$ due to the helical geometry.

For the double-helix plane-waves description of Eq. 8, the associated entropic Lagrangian *L*_*S*_ has no entropic potential term (that is, *V*_S_ = 0) since such a system is equivalent to that of a free particle. Appendix C (Supplementary Information) provides the proof that the entropic Lagrangian functional (see Eq. C.3):

13a $${L}_{S}=3{m}_{S}+\sum _{n=1,2,3}{m}_{S}\,{\rm{l}}{\rm{n}}\,{q{\rm{^{\prime} }}}_{n}=3{m}_{S}-\sum _{n=1,2,3}{m}_{S}\,{\rm{l}}{\rm{n}}({p}_{n}/{m}_{S})$$

as employed in Eq. 12 satisfies the Euler-Lagrange equations

13b $$\frac{{\rm{d}}}{{\rm{d}}{x}_{3}}\frac{{\rm{\partial }}{L}_{S}}{{\rm{\partial }}{q{\rm{^{\prime} }}}_{n}}-\frac{{\rm{\partial }}{L}_{S}}{{\rm{\partial }}{q}_{n}}=0\,(n\in \{1,2,3\})$$

demonstrating that the exertion *X* is at an extremum (or at least stationary) at any point along the length of the double helix since $\delta (\int {L}_{S}{\rm{d}}{x}_{3})=0$ (see Appendix C, Eq. C.22). Also, Appendix D (Eq. D.3b; both Appendices are in Supplementary Information) shows that the entropic Lagrangian for a double helix can be given by ${L}_{S}=3{m}_{S}-\pi {\kappa }_{0}{k}_{B}$; that is, in this case *L*_*S*_ is indeed a constant (invariant with *x*_3_). Note also that the exertion *X* is scaled by the quantum of entropy, Boltzmann’s constant, just as the Lagrangian itself is.

### *Entropy*

Having defined the exertion integral, Eq. 12, we can also now see that the equivalent space-trajectory integral of the entropic Hamiltonian *H*_*S*_ (see Eq. 11) yields a quantity directly proportional to the entropy:

14 $$S=\int {H}_{S}{\rm{d}}l=\chi \int {H}_{S}(q,p,{x}_{3}){\rm{d}}{x}_{3}$$

Whereas Eq. 6 describes a ‘local’ entropy *s*, the integrated quantity *S* can be considered as the ‘global’ or the overall system entropy. Eq. 14 indicates that the overall entropy *S* depends not only on the centroidal trajectory of the double helix axis as described by *x*_3_, but principally upon the spiralling path described by *l* with its radial dependency such that the entropy is a function of the full spatial extent (in all spatial dimensions) of the double helix structure. For convenience, we offset the entropic Hamiltonian *H*_*S*_ by the constant term *m*_*S*_ ln(*κ*_0_ *R*_0_) (see Appendix D in Supplementary Information, text prior to Eq. D.2a) which is an invariant for a double helical geometry – any Hamiltonian can be offset by a fixed (constant) amount to enable more convenient manipulation – such that the entropic Hamiltonian for a double helix can therefore be given as *H*_*S*_ = π*κ*_0_*k*_*B*_; that is, each KE component (*n* = 1, 2) of the double helix contributes ½π*κ*_0_*k*_*B*_. We can also exploit the Fourier (periodic) nature of *S* along the double helix as characterized by the parameter *iκ*_0_ to write the Fourier differential operator as:

15 $$\frac{{\rm{d}}}{{\rm{d}}{x}_{3}}\equiv i{\kappa }_{0}$$

Since the Lagrangian and Hamiltonian are inversely related (through the Legendre transformation) and the exertion integral *X* (Eq. 12) is at an extremum (Eq. 13b), δ*X* = 0, then the closely connected Hamiltonian trajectory integral Eq. 14 (that is, the entropy *S*) must also be at an extremum, δ*S* = 0. Given that the double helix of DNA represents a highly stable structure we infer from the Second Law that the entropy *S* is at a maximum; *ergo* the exertion *X* is at a minimum and the double helix topology represents a MaxEnt (most likely) trajectory in space. In summary, the overall entropy *S* of the double helix is given by (see Appendix D Eq. D.4):

16 $$S=\surd (1+{\kappa }_{0}^{2}{R}_{0}^{2})\pi {\kappa }_{0}L{k}_{B}$$

It is clear that the entropy *S* is proportional to the length *L* of the double helix. However, in the case of a photon its proper length is actually zero relativistically, since it travels at the speed of light: *L* = 0, therefore *S* = 0.

### *B-DNA and P-DNA*

**Figure 1:**
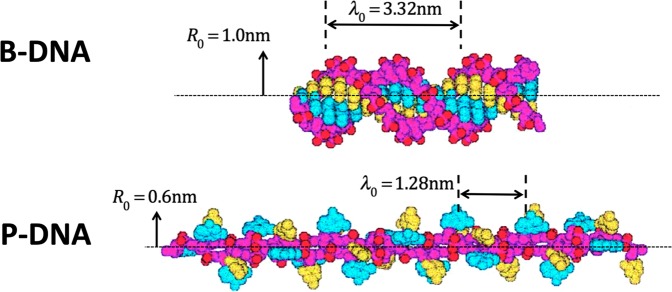
Two forms of DNA, with dimensions. Modified from Fig. 5 of Allemand *et al*. *Proc*. *Natl*. *Acad*. *Sci*. *USA* **95**, 14152–14157 (1998)^22^. Copyright (1998) National Academy of Sciences USA.

In an extraordinary mechanical experiment, Bryant *et al*.^21^ made a controlled transformation of B-DNA to P-DNA, where the latter is an artificial form called after Linus Pauling and discussed at length by Allemand *et al*.^22^ (see Fig. 1). Essentially, Bryant *et al*. held the B-DNA molecule (of length 4.681 μm) straight in tension (45 pN), and twisted it (4800 turns, with a torque of 34 pN·nm) until it had entirely transformed into the P-DNA form (with an extension of 2.8 μm). Thus, the mechanical energy expended to turn this B-DNA molecule into a P-DNA one is **1151 aJ** (126 aJ from the extension and 1025 aJ from the torque). To calculate the conformational energy changes with standard methods is computationally heavy: a recent molecular dynamics calculation by Liebl & Zacharias^23^ to determine free energies actually mimicked Bryant *et al.*’s experiment.

But determining the change in structural entropy (in this context equivalent to the Gibbs free energy change) is now straightforward for these holomorphic structures. Using Eq. 16, and *κ*_0_ ≡ 2π*/λ*_0_, where *R*_0_ = {1.0, 0.6} nm respectively for the B- and P- forms; *λ*_0_ = {3.32, 1.28} nm, and *L* = {4681, 7286} nm, we obtain from the geometric entropy (at 23 °C) the energies of the two forms of {244, 1428}aJ, yielding a change of **1184 aJ**. (Note that the Type A standard uncertainty just from Bryant *et al*.’s torque measurement is about 70 aJ.)

It is not entirely clear which values to assign to *R*_0_, especially for the case of P-DNA, with plausible values for the latter ranging between 0.4 and 0.8 nm. In any case, it is clear that a very simple calculation using the apparatus of geometrical thermodynamics is capable of a result entirely consistent with experiment, where this result is not available without heavy computation using standard methods in physical chemistry.

To explain the stability of fullerene molecules a similar comparison can be made between this simple geometrical thermodynamics and the heavy computation required by the standard physical chemistry methods (which now have a very extensive literature)^24^.

## The Double-Armed Logarithmic Spiral

**Figure 2:**
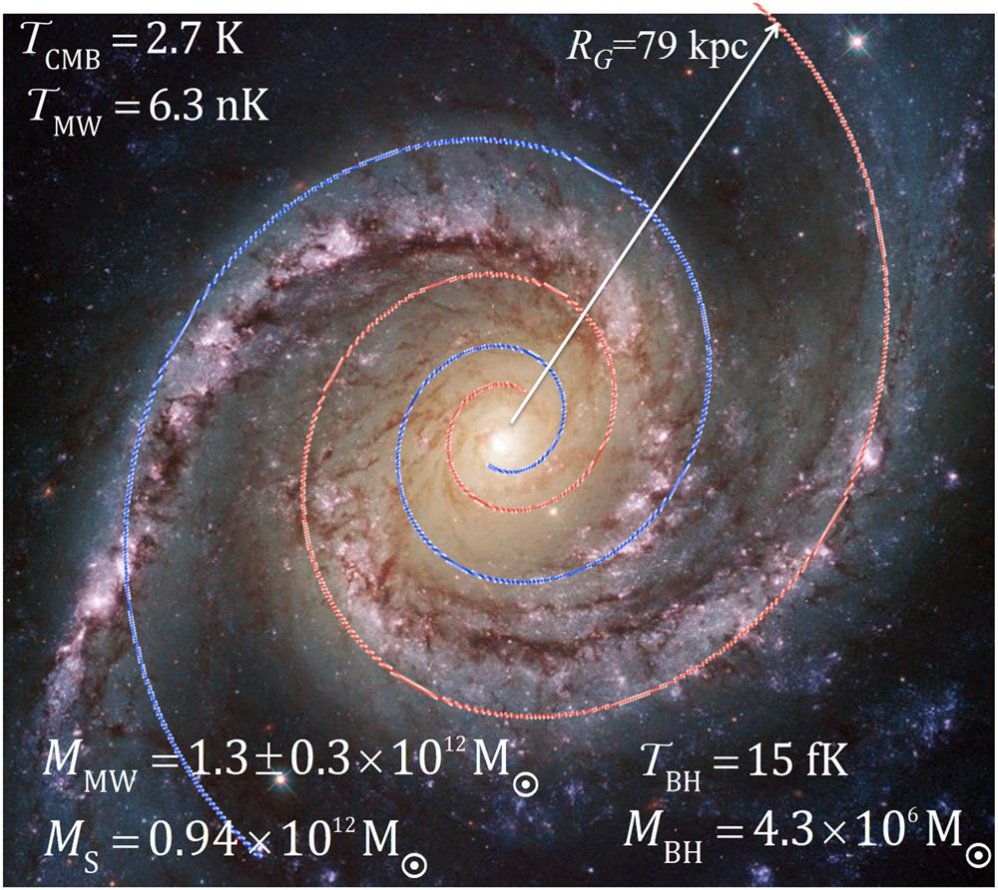
A spiral galaxy with an overlaid double-armed logarithmic spiral. Parameters given (see text) are of the Milky Way (MW): ${{\mathscr{T}}}_{BH}$ and *M*_BH_ are the (black body) temperature and mass of the super-massive black hole at the galactic centre; ${{\mathscr{T}}}_{CMB}$ is the cosmic microwave background temperature; *R*_*G*_, *L*, ${{\mathscr{T}}}_{MW}$ are the MW radius, thickness and “temperature”; *M*_MW_ is the MW observed virial mass; *M*_*S*_ is the mass implied by the MW entropy and ${{\mathscr{T}}}_{MW}$. All masses in units of solar mass M_◉_. Underlying Hubble image of NGC 1566, taken 2^nd^ June 2014 by NASA Goddard Space Flight Center https://www.flickr.com/photos/gsfc/14172908657/; licenced under CC BY 2.0 (https://creativecommons.org/licenses/by/2.0/).

Figure 2 shows NGC 1566, an intermediate spiral galaxy 40 million light-years away in the constellation of Dorado (southern hemisphere) and the second brightest Seyfert galaxy known. The Milky Way is known to have a similar geometry (but of course we have no comparable image of it) and some parameters of our galaxy, including a double-armed logarithmic spiral, are overlaid on the Figure. We will show that such a double-armed spiral is holomorphic, just as is the double-helix of the photon or of DNA.

However, we can immediately comment on the parameters of Fig. 2, which are largely determined by the mass *M*_*BH*_ of the central galactic black hole whose entropy *S*_*BH*_ (in SI units)

17 $${S}_{BH}=4\pi {k}_{B}G{M}_{BH}^{2}/c\hslash =1.78\times {10}^{90}{k}_{B}$$

is given by the Bekenstein-Hawking equation: see for example Eq. 2.2 in the review of Bousso^25^, or explicitly by Penrose (§27.10 p.716^15^) for a stationary black hole. As usual, *ħ* is the reduced Planck constant, *k*_*B*_ is Boltzmann’s constant, and *G* is the gravitational constant. But it is well known that the galactic entropy is dominated by the entropy of its central super-massive black hole (*S*_*MW*_ ≈ *S*_*BH*_: see for example the discussion in Penrose 2010^2^ §2.6 p.127), which has an equivalent black body temperature of (Bousso^25^ Eq. 2.8):

18 $${{\mathscr{T}}}_{BH}=\hslash {c}^{3}/8\pi G{M}_{BH}{k}_{B}=1.5\times {10}^{-14}{\rm{K}}$$

*M*_*BH*_ is given by Gillessen *et al*.^26^ as 4.3 ± 0.4 million solar masses M_◉_, where this 10% uncertainty is entirely due to the uncertainty in the galactic position of the Sun: the measurement actually has a precision better than 2% (the mass of the Sun is known very accurately, to about 10^−4^: M_◉_ = 1.989 × 10^30^ kg). Applying this temperature to *S*_*MW*_ to obtain the energy (given by the product of entropy and temperature expressed as a mass through *E* = *mc*^2^) we naturally recover *M*_*BH*_.

In a standard model of the Milky Way^27^ (a barred galaxy) the stellar disc is modelled as distinct “thin” and “thick” discs, with the “cut-off bulge radius” of the “thick disc” (or “bulge”) given as 1.9 kpc, and the total (virial) mass within a radius of 60 kpc being 4.0 ± 0.7 × 10^11^ M_◉_. Rix & Bovy^28^ explain that there is no well defined distinction between the “thin” and “thick” discs, but the characteristic “scale height” of the “thin disc” can be given approximately as 0.3 kpc. Patsis *et al*.^29^ show that the “bar” (bulge) of the galaxy can be described in orbital dynamics terms, and Saito *et al*.^30^ map the bulge (the “bar”) from observational data. We will assume an approximate galactic half-thickness *L*/2 = 1 kpc. The *parsec* is defined as the distance from the Sun of a star observed to have one second of arc annual stellar parallax, and is therefore relative to the diameter of Earth’s orbit (1 kpc = 3.09 × 10^19^ m).

Another study has concentrated on the galactic mass^31^: giving the observed stellar galactic mass and the virial mass respectively as 6.4 ± 0.6 × 10^10^ M_◉_, and 1.26 ± 0.24 × 10^12^ M_◉_. “Virial” mass includes so-called “dark matter” and is derived from the observations of stellar proper motions in large scale star surveys using the Virial Theorem (Clausius, 1870).

Consider a doubled-armed logarithmic spiral, as frequently observed for galaxies (see Fig. 2 which is a plan view projecting the 3-D object onto a plane). Here, the holomorphic functionals describing the *x*_1_ and *x*_2_ locus co-ordinates are now (in contrast to Eq. 8):

19 $${x}_{1}={R}_{G}{e}^{-{\rm{\Lambda }}({x}_{3}+L/2)}{e}^{i\kappa {x}_{3}}={r}_{BH}{e}^{-{\rm{\Lambda }}{x}_{3}}{e}^{i\kappa {x}_{3}}\,{\rm{and}}\,{x}_{2}=-\,i{R}_{G}{e}^{-{\rm{\Lambda }}({x}_{3}+L/2)}{e}^{i\kappa {x}_{3}}=-\,i{r}_{BH}{e}^{-{\rm{\Lambda }}{x}_{3}}{e}^{i\kappa {x}_{3}}$$

such that the instantaneous radius is ${R}_{n}={R}_{G}{e}^{-{\rm{\Lambda }}({x}_{3}+L/2)}={r}_{BH}{e}^{-{\rm{\Lambda }}{x}_{3}}$ for *n* = 1, 2 (see Eq. B.24 in Appendix B), where *r*_*BH*_ is the Schwarzschild radius (the event horizon) of the central black hole. The logarithmic radial parameter Λ is given by the requirement that the galactic radius *R*_*G*_ and the Schwarzschild radius *r*_*BH*_ are related logarithmically by the half-thickness *L*/2 (see Eq. D.15a in Appendix D, Supplementary Information): *r*_*BH*_ = *R*_*G*_ exp(−Λ*L*/2), or

20 $${\rm{\Lambda }}=(2/L)\times \,\mathrm{ln}({R}_{G}/{r}_{BH})$$

where for the Milky Way Λ = 26 kpc^−1^. The radius *r*_*BH*_ of the central galactic black hole is determined by the black hole mass *M*_*BH*_ to be 1.270 × 10^7^ km (about 18 times the solar radius; see Eq. D.14). The coupling coefficient *κ* is assumed to vary similarly to the radius, that is $\kappa ={\kappa }_{BH}\exp ({\rm{\Lambda }}{x}_{3})$ (Eq. B.24d), where *κ*_*BH*_ is the pitch at the black hole event horizon. The associated hyperbolic co-ordinates (using Eq. 9a, see Eq. B.33) are:

21 $$\begin{array}{ccl}{q}_{1} & = & i{r}_{BH}{e}^{-{\rm{\Lambda }}{x}_{3}}\kappa {x}_{3}\\ {q}_{2} & = & i{r}_{BH}{e}^{-{\rm{\Lambda }}{x}_{3}}(\kappa {x}_{3}-\pi /2)\\ {q}_{3} & = & {x}_{3}\end{array}$$

All quantities clearly revert to their respective double-helical quantities when the logarithmic spiral parameter Λ = 0. We find that a logarithmic spiral is associated with an entropic potential field *V*_*S*_ ≠ 0 causing a hyperbolic acceleration; indeed, as the entropic analogy to Newton’s second law of kinematics (*F* = *mẍ*), we solve the Euler-Lagrange equations (defined in hyperbolic space *q*_*n*_) ${\rm{d{p}}}_{n}/{\rm{d}{x}}_{3}=-\,{{m}_{S}}{q}_{n{\prime\prime}}/{q}_{n{\prime}}=-\,\partial {V}_{S}/\partial{qn_{2}}$, where the final term in the equation (the entropic potential gradient) is therefore equivalent to the entropic force *F*_*S*_. The associated entropic acceleration is given by ${\Gamma}_{n}=-{q}_{n{\prime\prime}}/q_{{n}{\prime}2}$, the minus sign being due to the inverse velocity nature of *q*′. The proof that the double-armed logarithmic spiral satisfies the Euler-Lagrange equations in hyperbolic space *q* (that is, obeys the principle of *least exertion*) is given in Appendix C (Eq. C.47, see Supplementary Information).

In Euclidean (*x*) space, we find that the entropic potential field *V*_*S*_ for the logarithmic double spiral is expressed as (see Eq. B.42 in Appendix B, Supplementary Information; *K*_0_ and *K*_3_ are dimensionless):

22 $${V}_{S}(x)=\frac{i{m}_{S}{K}_{0}{e}^{i{\kappa }_{G}{x}_{3}}}{1-{\rm{\Lambda }}{x}_{3}}(\frac{{x}_{1}+i{x}_{2}}{{x}_{1}{x}_{2}})-\frac{{m}_{S}{K}_{3}{e}^{{\rm{\Lambda }}{x}_{3}}}{{R}_{3}(1-{\rm{\Lambda }}{x}_{3})}$$

It is indeed interesting to note the existence of an inverse-square law (in Euclidean space) for the *γ*_1_ and *γ*_2_ directions at the heart of this entropic potential field; the entropic force varies as

23 $${F}_{S,n}=-\,\frac{\partial {V}_{S}}{\partial {x}_{n}}=-\,\frac{{m}_{S}{K}_{0}{e}^{i{\kappa }_{G}{x}_{3}}}{{x}_{n}^{2}(1-{\rm{\Lambda }}{x}_{3})}\,n=1,2$$

that is, *F*_*S*,*n*_ ∝ *x*_*n*_^−2^, with *F*_*S*_ also being proportional to the entropic mass *m*_*S*_ assumed located at the centre of the system and to be the cause of the entropic potential field. We emphasise, however, that although Eqs 22 and 23 express the entropic field in a more intuitive Euclidean form, the entropic Hamiltonian and Lagrangian equations are only correctly applied in hyperbolic space.

The general shape of the Milky Way is closely determined by these holomorphic logarithmic spirals. In particular, it can be shown that the ratio of the extremal radius *R*_*G*_ to the full-thickness *L* is (see Appendix D, Eq. D.17, Supplementary Information):

24 $${R}_{G}/L=4{\pi }^{2}$$

The radius *R*_*G*_ is rather poorly defined observationally, and the estimate *L*/2 = 1 kpc given above implies (from Eq. 24) *R*_*G*_ = 79 kpc, which is within the range usually given: therefore Eq. 24 has some observational support. The Milky Way in reality has a complex structure involving multiple spiral arms, a central “bulge”, and oscillating star densities reported recently^32^ as persisting to much larger distances than *R*_*G*_. None of this is considered in our zeroth order model. The present treatment should also be regarded as a static approximation neglecting the dynamic mechanisms of galactic formation and evolution. Figure 2 shows only the plan view of the model: the galactic cross-section is here modelled as a disc of essentially uniform thickness *L*, dimpled at its centre (that is, ignoring the “bulge” altogether).

The spiral coordinate *x*_3_ projected onto the plane in Fig. 2 is associated with an azimuthal angle *θ* = *κ*_*G*_*x*_3_ where the appropriate wavelength scale *λ*_*G*_ for the galaxy is given by the galactic wavenumber *κ*_*G*_ = 2π/*λ*_*G*_. This can be calculated from the galactic structural entropy *S*, well approximated by (Appendix D, Eq. D.13b, Supplementary Information):

25 $$S=A{\kappa }_{G}^{2}{k}_{B}/2$$

where *A* ≡ 2π *R*_*G*_ *L* closely approximates the area of an ellipsoid of radii *L*/2 and *R*_*G*_. Eq. 25 for the logarithmic spiral is exactly equivalent to Eq. 16 for the double-helix. Thus we get an expression for the galactic wavelength *λ*_*G*_ (see Eq. D.16 in Appendix D)

26 $${\lambda }_{G}=(2\pi \cdot {l}_{P}/{r}_{BH})\cdot \surd ({R}_{G}L)=1.059\times {10}^{-24}{\rm{m}}$$

where *l*_*P*_ = 1.616 × 10^−35^ m is the Planck length.

Eq. 25 is startling. The galactic entropy (which is almost exactly the central supermassive black hole entropy) is given “holographically” (see Appendix D, Eq. D.5 and subsequent discussion) by the surface area of the galaxy, just as the black hole entropy is determined by the surface area of the event horizon. We now postulate that just as for the black hole, a “temperature” ${{\mathscr{T}}}_{MW}$ can be defined at this holographic surface. This temperature must lie between the central supermassive black hole temperature (15 fK) and the cosmic microwave background temperature of 2.73 K.

To obtain a reasonable estimate of ${{\mathscr{T}}}_{MW}$ we note that the power radiated from a spherical black body surface of radius *R* and temperature ${\mathscr{T}}$ is 4π*σ* (*R*${\mathscr{T}}$^2^)^2^, where *σ* is the Stefan-Boltzmann constant. We therefore highlight here the appearance of the composite quantity *R*${\mathscr{T}}$^2^ that appears as a consequence of the Stefan-Boltzmann law. With both *R* and ${\mathscr{T}}$ increasing exponentially with distance from the galactic centre, it is clear the resulting large temperature gradient along *x*_3_ implies a large energy flow towards and into the black hole: the galaxy is not in thermal equilibrium! However, to at least maintain some thermal ‘stability’ along the *γ*_3_ axis, we might assume that ${\mathscr{T}}$^2^ varies similarly to *R*, so that the black body power inwardly radiated from each spherical surface along the *γ*_3_ axis maintains some continuity. Relying on the isomorphism between the double-helix and the logarithmic double-spiral in hyperbolic space we therefore consider ${\mathscr{T}}$^2^ to vary with exp(−Λ*x*_3_) just as *R* and the galactic wavelength *λ* do (see Eq. 19 and Eq. B.24 in Appendix B):

27a $$R={r}_{BH}\exp (\,-\,{\rm{\Lambda }}{x}_{3})$$

27b $${\mathscr{T}}={{\mathscr{T}}}_{BH}\exp (\,\,-\,\,{\rm{\Lambda }}{x}_{3}/2)$$

Then, at *x*_3_ = −*L*/2 with Λ = 26 kpc^−1^, we have ${\mathscr{T}}$ = ${{\mathscr{T}}}_{MW}$ = 6.3 nK for *L* = 2 kpc giving *M*_MW_ = 0.94 × 10^12^ M_◉_ (and *R*_*G*_ = 79 kpc) consistent with observation. To obtain the central observed value for the virial mass of the Milky Way of *M*_MW_ = 1.26 × 10^12^ M_◉_, we need *L* = 3.6 kpc (giving *R*_*G*_ = 142 kpc and ${{\mathscr{T}}}_{MW}$ = 8.4 nK).

To summarise: we have shown that the structure of the galaxy for which we have detailed experimental observations (that is, the Milky Way) is consistent with a holomorphic representation in geometric algebra. In particular, we have shown that the galactic *shape*, *aspect ratio*, and *structural stability* (which are all highly constrained by the algebra) are consistent with observation; and we have also shown that the total galactic *mass* is also consistent with observation. Note that this is a simplified (“zeroth order”) analytical approximation to reality: for example, the black hole angular momentum is neglected, as are the dynamics driving the galactic evolution. Also, we have not started to consider the perturbation problem implied by deviations of the star population from the ideal logarithmic spiral; although we would anticipate that the principle of least exertion causes an entropic force to be exerted so as to maintain the MaxEnt galactic structure.

Notwithstanding the approximations, these results are very surprising, because they underline the dominant effect that the central super-massive black hole has on the galactic structure. In fact, this treatment gives the proper weight to the effect of the black hole entropy, which is certainly not hidden away behind the event horizon.

## Isomorphism between Mechanics and Entropy

Table 1 shows the multiple isomorphisms that exist between kinematic and entropic quantities revealed by our treatment. There has been significant recent interest in comparable methods. Baez & Pollard^33^ argue for an “analogy” between thermodynamics and quantum mechanics, giving rise to a quantity they call “quantropy” (quantum entropy, which they call “mysterious”). They also give a Table of “analogies” between statistical and quantum dynamics comparable to our Table of isomorphisms. We believe that our results confirm and extend this approach. Velazquez^34^ has also tabulated some consequences of the complementarity of the Planck and Boltzmann constants. Dixit *et al*.^35^ have reviewed the use of “Maximum-Caliber” to characterise trajectories (“world-lines”) in non-equilibrium thermodynamics (where “*caliber*” is a term introduced by Jaynes^36^ to characterise the evolution in space-time of the ensemble of trajectories of microstates; it is proportional to our “*Exertion*”).

**Table 1:** Isomorphism between kinematic and entropic quantities.

Quantity	Kinematic (Conventional)	Entropic Equivalent	See near Eqs
Physical constant	Planck, *ħ* [Js]	Boltzmann, *k*_*B*_ [JK^−1^]	9c, 12
Space-time co-ordinates, *q*	*x*_1_, *x*_2_, *x*_3_, *x*_0_ (≡*ct*) (Euclidean, Minkowski)	${q}_{n}={R}_{n}\,\mathrm{ln}(\frac{{x}_{n}}{{R}_{n}})$ (hyperbolic, Minkowski)	1, 9a, 21
Differential operator	∇ = ∂/∂*x*_*n*_	∇_*q*_ = ∂/∂*q*_*n*_	11
Wavelength, *λ* & wavenumber *κ*	*λ*(=2π*c*/*ω*) & *k* = 2π/*λ*	*λ* = helical pitch; *κ* = 2π/*λ*	5
Time-like axis & associated Fourier differential	$t,\frac{{\rm{d}}}{{\rm{d}}t}\equiv i\omega$	${x}_{3},\frac{{\rm{d}}}{{\rm{d}}{x}_{3}}\equiv i\kappa$	11, 15
Momentum, *p*	$p=m\dot{x}=\frac{2\pi \hslash }{\lambda }$	$p={m}_{S}{v}^{-1}=\frac{{m}_{S}}{q^{\prime} }=\frac{{k}_{B}}{R}$	9b, 10
Velocity, *v*	${\dot{x}}_{n}=\frac{{\rm{d}}{x}_{n}}{{\rm{d}}t}\,n=1,2,3$	$v\& {v}^{-1}\equiv {q^{\prime} }_{n}=\frac{{\rm{d}}{q}_{n}}{{\rm{d}}{x}_{3}}={R}_{n}\frac{{x^{\prime} }_{n}}{{x}_{n}}$ (i.e. also has inverse velocity, *v*^−1^, characteristics)	9b, 10
Mass, *m*	$m(\equiv \frac{k\hslash }{c})\,[{\rm{kg}}]$	${m}_{S}\equiv {i\kappa k}_{B}\,[{\rm{J}}{{\rm{K}}}^{-1}{{\rm{m}}}^{-1}]$	9c, 15
Kinetic term $T=\int p{\rm{d}}v$	$T=\int p{\rm{d}}\dot{x}\,=$ ½ $m{\dot{x}}^{2}$	${T}_{S}=-\,\int p{\rm{d}}q{\rm{^{\prime} }}=-\,{m}_{S}\,{\rm{l}}{\rm{n}}q{\rm{^{\prime} }}\,=$ ½ ${m}_{S}\,{\rm{I}}{\rm{n}}\,{q{\rm{^{\prime} }}}^{2}$	10
Potential term, *V*	$V=m\ddot{x}x\equiv mgx$	${V}_{S}(q)={m}_{S}(\,-\,q{\rm{^{\prime} }}{\rm{^{\prime} }}/{q{\rm{^{\prime} }}}^{2})q\equiv {m}_{S}{\rm{\Gamma }}q$	11, 22
Hamiltonian, *H*	*H* = *T* + *V*	${H}_{S}={T}_{S}+{V}_{S}=\sum _{n=1}^{3}-{m}_{S}\,\mathrm{ln}\,{q^{\prime} }_{n}+{V}_{S}({q}_{n})$	11
Lagrangian, *L*	$L=\sum _{n=1}^{3}\,{p}_{n}{\dot{x}}_{n}-H=T-V$	${L}_{S}=\sum _{n=1}^{3}\,{p}_{n}{q^{\prime} }_{n}-{H}_{S}=3{m}_{S}-{H}_{S}$	11, 13a
Newton’s 2^nd^ Law: [Euler-Lagrange formulation]	$\frac{{\rm{d}}}{{\rm{d}}t}\frac{\partial L}{\partial {\dot{x}}_{n}}-\frac{\partial L}{\partial {x}_{n}}=0$	$\frac{{\rm{d}}}{{\rm{d}}{x}_{3}}\frac{\partial {L}_{S}}{\partial {q^{\prime} }_{n}}-\frac{\partial {L}_{S}}{\partial {q}_{n}}=0$	13b, 21
*F* = *ma*	*F* = −∇*V* = *m*$\ddot{x}$	${F}_{S}=-\,{{\rm{\nabla }}}_{q}{V}_{S}=-{m}_{S}q{\rm{^{\prime} }}{\rm{^{\prime} }}/{q{\rm{^{\prime} }}}^{2}={m}_{S}{\rm{\Gamma }}$	21, 23
Acceleration	$\ddot{x}$ = $g[{{\rm{m}}{\rm{s}}}^{-2}]$	$-q{\rm{^{\prime} }}{\rm{^{\prime} }}/{q{\rm{^{\prime} }}}^{2}={\rm{\Gamma }}\,[{{\rm{m}}}^{-1}]$	21, 23
Action/Exertion Integral	= $\int L{\rm{d}}t\,[\hslash ]$	$X=\chi \int {L}_{S}{\rm{d}}{x}_{3}\,[{k}_{B}]$	12
Variational Principle	δ = $\delta \int L{\rm{d}}t=0,$ Least Time/Action	$\delta X=0,\,\delta \int {L}_{S}{\rm{d}}{x}_{3}=0,$ Least Exertion	12, 13b
Entropy	$S=\int \frac{{\rm{d}}E}{{\mathscr{T}}}$	$S=\chi \int {H}_{S}{\rm{d}}{x}_{3}$	14, 16, 25
Maximum Entropy	$\delta \int H{\rm{d}}t=0,$ Stationary Phase (Group Velocity)	$\delta S=0,\,\delta \int {H}_{S}{\rm{d}}{x}_{3}=0$	15

Considering Table 1, we have already observed that the hyperbolic Minkowski space (generated through the normalising Euclidean metric, *R*_*n*_) is the entropic analogue to the Euclidean Minkowski space of kinematics, with consequent del operators; that Boltzmann’s constant is the entropic quantum analogue to Planck’s quantum of action (also pointed out by Córdoba *et al*.^37^) with consequently analogous definitions for momentum; and that the helical pitch (or wavelength) implies the space-like entropic analogue of time in kinematics. As we have seen, this latter also implies holographic properties of the treatment (that is, properties of an area being fully equivalent to parameters of a volume).

Both mass and its entropic equivalent *m*_*S*_ have natural units of inverse length, but *m*_*S*_ is imaginary as a consequence of the holomorphism (Eq. 9c). The parameter Λ describing a logarithmic spiral contributes to the entropic (hyperbolic) acceleration Γ as a consequence of an entropic force, in analogy to Newton’s 2^nd^ Law; and the double-helix can be seen as a special case (Λ = 0) of the double-armed logarithmic spiral. The Hamiltonian and Lagrangian formulations then follow equivalently for both energy and entropy, with the *Exertion* integral equivalent to the classical *Action* integral, both obeying the principle of stationary “action”.

## Summary

Formal mathematics establishes tautologies which are frequently very surprising, and we have used well-established formal methods in a properly quantitative treatment of entropy, revealing that measurable (and measured) quantities from the molecular to the galactic scale can be readily calculated in a simple analytical treatment. We have considered systems of high symmetry which are amenable to our simplified analytical approach, but we expect the method to be readily generalisable to more complex systems.

The computational demands of conformational chemistry are very severe; perhaps this approach will stimulate algorithmic advances to speed the calculations for static problems, or even to address dynamic geometrical problems (like protein folding) in new ways?

We have used a “toy” model of the Milky Way, which ignores the central “bulge” and multiple arms, but a more realistic model already available would simply take a linear combination of a spherical central feature^24^ and multiple double-spiral arms. The difficulty here is not in the modelling but in the choice of realistic observational data for the model parameters.

## Supplementary information

Appendices A-D
