# Supplementary material for: Maximum Entropy (Most Likely) Double Helical and Double Logarithmic Spiral Trajectories in Space-Time
Source: Sci Rep. 2019 Jul 25;9:10779. doi: 10.1038/s41598-019-46765-w (PMC6658702; doi:10.1038/s41598-019-46765-w)
Supplement: Supplementary file 1 — Appendices A-D [file 41598_2019_46765_MOESM1_ESM.pdf]

Maximum Entropy (Most Likely) Double Helical and Double Logarithmic Spiral Trajectories in Space-Time

M.C.Parker, School of Computer Sciences & Electronic Engineering, University of Essex, Colchester, UK
C.Jeynes, University of Surrey Ion Beam Centre, Guildford, UK

Appendices A, B, C, and D

Table of Contents

Appendix A: Derivation of Geometric Entropy using the Clifford Algebra 3
Abstract 3
Information of a Simple Pole 3
Information Integral 4
Quaternion Subalgebra 5
Spinor Isomorphism 5
Maxwell's Equations using Geometrical Algebra 6
Chirality Revealed 8
References 9
Annex - Derivation of Equation Eq.A2 9
Calculation of Square-Integrability of $\phi_n(ct)$ 10
Calculation of Information h_n 11
Appendix B: Entropic Hamiltonian & Lagrangian Analysis 14
Abstract 14
Introduction 14
The Double Helix 14
Trajectory Equations 14
Digression 16
Kinetic & Potential Entropy 17
Entropic Hamiltonian & Lagrangian Canonical Relations 18
Canonical Relations for a Holomorphic Double Helix 19
Conservation of Entropic Momentum and Entropic Hamiltonian 20
Application to a Holomorphic Logarithmic Double Spiral 20
Trajectory Equations 20
Logarithmically varying coupling coefficient κ 23
Entropic analogue to Newton's second law of motion 24
q_1-coordinate 25
q_2-coordinate 27
q_3-coordinate 29
Overall Entropic Hamiltonian of Holomorphic Logarithmic Double Spiral 30
Overall Entropic Field 30
Concluding Remarks 31
Annex: holomorphism demonstration 32
Holomorphism of a Double Helix 32
Holomorphism of a Logarithmic Double Spiral 33

Appendix C: Euler-Lagrange Equations applied to the Principle of Least Exertion **35**
Abstract **35**
Exertion **35**
Double Helix **35**
q_1-coordinate 36
q_2-coordinate 36
q_3-coordinate 37
Summary 37
Double-Armed Logarithmic Spiral **38**
q_1-coordinate 39
q_2-coordinate 39
q_3-coordinate 40
Summary 41
Appendix D: Geometric Entropy Analysis of Double Helical or Spiral Structures: DNA & the Milky Way Galaxy . **42**
Abstract **42**
Calculation of the Entropy of a Double Helix **42**
Geometric Interpretation for Structural Entropy of Double Helix 43
Calculation of the Geometric Entropy of a Double Spiral Galaxy **44**
Calculating the Galactic Wavelength λ_G 46

Appendix A

Appendix A: Derivation of Geometric Entropy using the Clifford Algebra

Abstract

We formally derive the information and entropy vectors of a simple pole by integrating over all time consistently in the positive time direction. This is rigorously transformed to a contour integral in 4-space using a full geometrical algebra treatment in Minkowski space-time. The geometrical algebra facilitates the expression of the simple relationship between entropy and information: they are Hodge duals (that is, they are equivalent to the electric and magnetic fields of Maxwell's equations). We use the complex-vector form to derive a simple algebraic expression for the local entropy associated with a helical trajectory. We also make clear not only the quaternion sub-algebra and its isomorphism with the spinor representation but also the intrinsic chirality of the double helix.

Information of a Simple Pole

We calculate expressions for the entropy due to a simple pole (point of non-analyticity) in 1+3 space-time, such that in general the simple pole is given by the meromorphic function:

$$\varphi(x)=\sqrt{\frac{x_{\text{pole}}}{\pi}}\frac{1}{x-x_{\text{pole}}} \tag{A.1a}$$

where x_{pole} is the location of the pole in 1+3 space-time, such that $x_{\text{pole}}=ct_0\gamma_0+x_1\gamma_1+x_2\gamma_2+x_3\gamma_3$, where the orthonormal space vectors $\{\gamma_\mu\}$ for $\mu=0,1,2,3$ obey the D = 1+3 Clifford algebra for Minkowski space-time, such that $\gamma_0^2=1$, while $\gamma_1^2=\gamma_2^2=\gamma_3^2=-1$, the basis vectors anti-commute, $\gamma_\mu\gamma_\nu=-\gamma_\nu\gamma_\mu$ for $\mu,\nu=0,1,2,3$ and $\mu\neq\nu$, we have the 1+3 pseudoscalar $\underline{i}=\gamma_0\gamma_1\gamma_2\gamma_3$, and $x_0=ct_0$ with c being the vacuum speed of light, so that we can write the general co-ordinates as $x=\chi_\mu\gamma^\mu$, where the Einstein summation rule applies.

By convention, we make the distinction between entropy and information, by defining information as being integration along the time-like axis (whilst entropy is integration along a space-like axis) as indicated in the Figure A.1 below. The complex (i.e. real and imaginary) nature of space-time allows us to perform a contour integral across a 2D complex plane as shown in Figure A.1.

We show a 1+1 space-time representation of the pole in one of the three 2D planes available (each containing the time-like axis). Thus the fact that entropy is given by integration along a space-like axis means that in 3D space, there are three different values contributing to the entropy, i.e. entropy is a vector quantity. Figure A.1 shows the $\gamma_0-\gamma_1$ plane example (for n=1), with the other space axes (n=2,3) being equally valid. We project the equation (A.1a) onto the $\gamma_0-\gamma_1$ plane such that the meromorphic expression for the amplitude of the projected pole in the two-dimensional $x_{01}=ct\gamma_0+\chi_1\gamma_1$ space-time is (i.e. the projection of Eq.A.1a):

$$\varphi_1(x_{01})=\sqrt{\frac{x_1\gamma_1}{\pi}}\frac{1}{x_{01}-x_{\text{pole1}}} \tag{A.1b}$$

where the location of the projected pole in the $\gamma_0 - \gamma_1$ plane (i.e. x_{01}) is given by $x_{\text{pole1}} = ct_0\gamma_0 + x_1\gamma_1$ (as per Figure A.1), the amplitude-squared magnitude of the pole distribution is given by $\rho_1 = \varphi_1\varphi_1^*$ (where * denotes the complex conjugate), and ρ_1 is square-integrable, with $\int_{-\infty}^{\infty} \rho_1 cdt = \gamma_1\gamma_0$ (see Annex for proof of this). Similarly for projecting onto the other two space dimensions (n = 2,3).

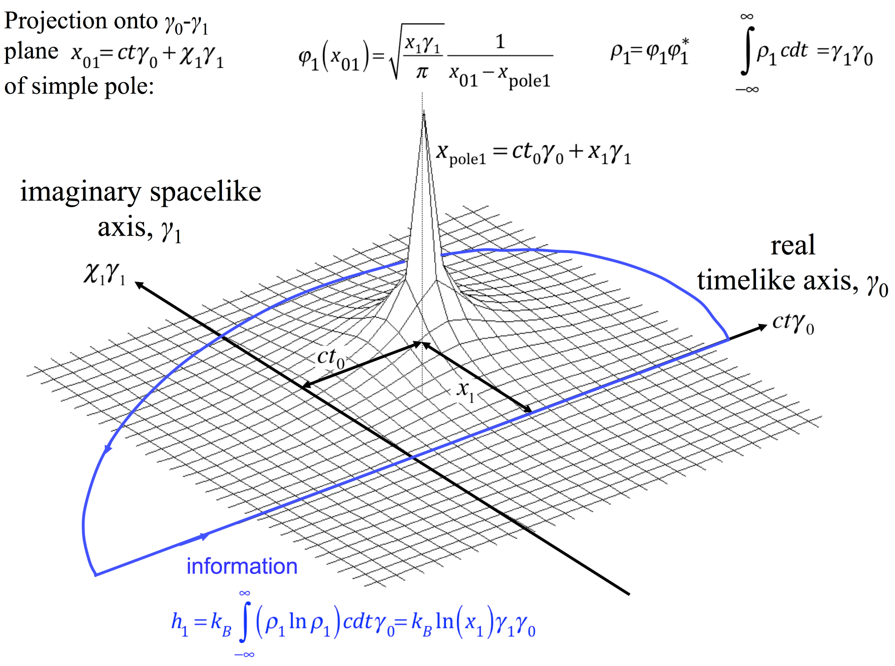

Figure A.1: Projection of a simple pole onto the $\gamma_0 - \gamma_1$ space-time plane, with resulting information calculated by integration along the time-like axis.

As previously indicated (Parker & Walker, 2010 [1]), we define the information of the pole by integrating over all (infinite) time consistently in the positive time direction. The integration is performed piecewise in 4-space by using the residue theorem separately for all three space axes and then summing the results.

Information Integral

The general integral expression for the *information* is given by [2] $h = k_B \int_{-\infty}^{\infty} \rho \ln \rho \, cdt$ where k_B is the Boltzmann constant, $\rho = \varphi(x)\varphi^*(x)$, with the asterisk denoting the complex conjugate. Note that $\rho_n(ct)$ is a causal and stable function that obeys the Paley-Wiener criterion [10].

However, as already indicated, the calculation of the information can be made using a contour integral across a complex plane (of which there are therefore 3 possibilities); and indeed, are projections of $\varphi(x)$ onto the appropriate one of the $\gamma_0 - \gamma_1$, $\gamma_0 - \gamma_2$ and $\gamma_0 - \gamma_3$ planes; i.e. in general, $\varphi_n(x_{0n})$ is the projection of $\varphi(x)$ onto the $\gamma_0 - \gamma_n$ plane. We note the requirement that the integration along the time axis be always in the positive time direction, forces us to always position the γ_0 timelike axis in the same location in the contour integral (see Fig. A.1, and equation (A.2) below.) Thus the calculation of the nth information bivector h_n (n = 1,2,3) is:

$$h_n = k_B \int_{-\infty}^{\infty} (\rho_n \ln \rho_n) cdt\gamma_0 = k_B \left[\oint \rho_n \ln \rho_n dx_{0n} \right] = k_B \ln(x_n)\gamma_n\gamma_0 \quad \text{(A.2)}$$

The resulting scalar quantity $k_B \ln(x_n)$ is multiplied by the bivector $\gamma_n\gamma_0$, which therefore has one edge always in a time-like direction, and another edge in a space direction. The proof of (A.2) is given in the Annex.

Quaternion Subalgebra

The bivectors together form an even subalgebra, where $\sigma_n = \gamma_n\gamma_0$, ($n$ = 1,2,3), where the σ_n are isomorphic to the Pauli spin vectors and also conform to the quaternion description. As an even subalgebra, the σ_n obey $\sigma_n^2 = 1$ and $\sigma_n\sigma_m = -\sigma_m\sigma_n$, and the pseudoscalar for this even subalgebra is $I = \sigma_1\sigma_2\sigma_3$, with $I^2 = -1$. Finally, the vectors σ_n can also be understood to be unit vectors along the co-ordinate axes of the 3-dimensional space. As such, the information due to a pole can be expressed as $h = k_B \ln(x_n)\sigma^n$ (n = 1,2,3), where the summing convention over n is assumed, and the x_n are the space co-ordinates of the pole. The entropy and information associated with a double-helical topology within a hyperbolic geometry is completely analogous to that of the photon. Since the electric and magnetic fields of Maxwell's equations are an example of a set of hyperbolic Cauchy-Riemann equations in space-time, with the electric and magnetic fields being the Hodge duals of each other (e.g. see Penrose, 2004, §19.2) we therefore also assume that entropy is the Hodge-dual of information, and vice-versa. The n = 0 case is null, since the contour integral has zero area:

$$h_0 = 0, \quad *h_0 = s_0 = 0 \tag{A.3}$$

For completeness we write the information h and its entropy (i.e. Hodge) dual s as follows, where we note the different indices n and m indicate the orthogonal complement is therefore taken as appropriate (i.e. $\sigma^m = *\sigma_n = I\sigma^n$):

$$h = k_B \ln(x_n)\sigma^n, \quad s = k_B \ln(x_m) I\sigma^m \qquad \text{(summing convention: } m,n\text{=1,2,3)} \tag{A.4}$$

Spinor Isomorphism

The isomorphism between the real-valued 4-dimensional (quaternion) vector space and the 2-dimensional complex spinor space is well documented (see for example Pandey & Chakravarti [3]):

$$|s\rangle = \begin{pmatrix} s_0 + is_3 \\ -s_2 + is_1 \end{pmatrix} \leftrightarrow \psi = s_0 + s_n I\sigma^n \tag{A.5}$$

where the pseudovector $I\sigma_n$ (i.e. the Hodge dual) is used here for the 4D (quaternion description). Parenthetically, we note for completeness that Hamilton's quaternions identify as follows [4]: $\mathbf{i} = -I\sigma_1$, $\mathbf{j} = I\sigma_2$, and $\mathbf{k} = I\sigma_3$. We also note, for clarity, that we distinguish $\mathbf{i}$ (here one of the quaternion basis vectors), $\underline{i}$ (the 1+3 pseudoscalar $\gamma_0\gamma_1\gamma_2\gamma_3$), I (the pseudoscalar $\sigma_1\sigma_2\sigma_3$ for the quaternion subalgebra) and i (the usual imaginary unit scalar).

Thus we see that the spin up component is given by $|s_\uparrow\rangle = s_0 + is_3$, and the spin down component is given by $|s_\downarrow\rangle = -s_2 + is_1$. The 2-component (Pauli) entropy spinor column vector can therefore be written as:

$$|s\rangle = \begin{pmatrix} |s_\uparrow\rangle \\ |s_\downarrow\rangle \end{pmatrix} \begin{pmatrix} ik_B \ln(x_3) \\ -k_B \ln(x_2) + ik_B \ln(x_1) \end{pmatrix} \tag{A.6a}$$

The spinor formalism shows the key geometric dependencies of the two eigenstates associated with the entropy spinor. In particular, considering the "spin down" spinor, we can see that the two space co-ordinates x_1 and x_2 (transverse to the γ_3-axis) are important for the "spin down" entropy quantity; whereas for the "spin up" spinor, it is the x_3 space (and, implicitly, the time axis $x_0 \equiv ct$) co-ordinates that play the important roles. The physical (geometric) interpretation [5] of the "spin down" spinor is straightforward to understand in the subsequent entropy analysis, whereas the "spin up" spinor is less easy to physically interpret. It is straightforward to confirm that the 2nd rows of Eqs.A.5 and A.6a are equivalent to writing for the entropy eigenvector: $\left|s_\downarrow\right\rangle \equiv i\left(s_1 I\sigma^1 + is_2 I\sigma^2\right) = is_1 I\sigma^1 - s_2 I\sigma^2$, or, taking the spin-down component as the physically interpretable part of the entropy eigenvector s:

$$s = i\left(s_1 I\sigma^1 + is_2 I\sigma^2\right) = is_1 I\sigma^1 - s_2 I\sigma^2 = k_B\left(i\ln(x_1)I\sigma^1 - \ln(x_2)I\sigma^2\right) \qquad \text{(A6b)}$$

Maxwell's Equations using Geometrical Algebra

We assume that the entropy s and information h are coupled with each other in an analogous fashion to the electric and magnetic fields in the Maxwell equations [6]. Equivalent to the Riemann-Silberstein complex vector for the 1-vector electric and magnetic fields [7], $\underline{F} = (\underline{E} + \underline{i}c\underline{B})\gamma_0$, we combine the information and entropy fields into the equivalent complex info-entropy bivector quantity: $f = s + Ih$, The overall complex entropy vector f can therefore be described using complex quaternions, which are isomorphic with a 4-component column bispinor:

$$\left|f\right\rangle = \begin{pmatrix} s_0 + is_3 \\ -s_2 + is_1 \\ -h_3 + ih_0 \\ -h_1 + ih_2 \end{pmatrix} \leftrightarrow f = s_0 + s_n I\sigma^n + I\left(h_0 + h_n I\sigma^n\right) \qquad \text{(A.7a)}$$

The information eigenvector h is represented in the final (4th) row of the LHS of Eq.A.7a by:

$$h \equiv -iI\left(-h_1\sigma^1 + ih_2\sigma^2\right) \qquad \text{(A.7b)}$$

Hence, we create a 4-component entropic bispinor as follows:

$$\left|f\right\rangle = \begin{pmatrix} \left|f_{\Uparrow\uparrow}\right\rangle \\ \left|f_{\Uparrow\downarrow}\right\rangle \\ \left|f_{\Downarrow\uparrow}\right\rangle \\ \left|f_{\Downarrow\downarrow}\right\rangle \end{pmatrix} = \begin{pmatrix} ik_B\ln\left(x_3\right) \\ -k_B\ln\left(x_2\right) + ik_B\ln\left(x_1\right) \\ -k_B\ln\left(x_3\right) \\ -k_B\ln\left(x_1\right) + ik_B\ln\left(x_2\right) \end{pmatrix} \qquad \text{(A.8)}$$

Such a four-vector aspect to the overall entropy spinor description is formally required by relativity. However, in the following analysis, we focus only on the "spin down" quantity $\left|f_{\Uparrow\downarrow}\right\rangle$ with its more obvious physical interpretation.

Another key aspect to our analysis is the "holomorphic pairing" description of two entropic trajectories, $\Sigma = l_1 + il_2$, which therefore acts as another type of holomorphic Riemann-Silberstein complex vector. We consider the l_2 trajectory to have C2 symmetry with respect to l_1, so that l_2 travels in the negative γ_3 direction. In order to maintain the same handedness as the l_1 trajectory, we find that the γ_1 axis of l_2 also has to change sign (according to the parity (P) requirements of spatial

inversion), but the γ_2 co-ordinate maintains its sign so as to maintain the appropriate handedness of the trajectory; i.e. the complex conjugate of the spinor is taken in the case of spatial inversion [8]. Reading from the equation (A.8), the entropy "spin down" spinor of interest associated with the trajectory l_1 is therefore:

$$\left|f_{\Uparrow\downarrow 1}\right\rangle = -k_B \ln\left(x_2\right) + ik_B \ln\left(x_1\right) \tag{A.9a}$$

whilst the entropy "spin down" spinor associated with the trajectory l_2 is given by:

$$\left|f_{\Uparrow\downarrow 2}\right\rangle = \left|f_{\Uparrow\downarrow 1}\right\rangle^* = -k_B \ln\left(x_2\right) - ik_B \ln\left(x_1\right) \tag{A.9b}$$

The spatially-inverted (complex conjugated) version of the information eigenvector h of Eq.A.7b can now be expressed as:

$$h = -iI\left(-h_1\sigma^1 + ih_2\sigma^2\right) = I\left(is_2 I\sigma^1 - s_1 I\sigma^2\right) = k_B\left(\ln(x_1)\sigma^2 - i\ln(x_2)\sigma^1\right) \tag{A.9c}$$

Hence, the overall "spin down" entropy spinor for the overall entropic trajectory $\Sigma = l_1 + il_2$ is given by:

$$\left|f_{\Uparrow\downarrow}\right\rangle = \left|f_{\Uparrow\downarrow 1}\right\rangle + i\left|f_{\Uparrow\downarrow 2}\right\rangle = \left(i+1\right)k_B \ln\frac{x_1}{x_2} \tag{A.10a}$$

or, in quaternion notation, where $f = s + Ih$,

$$f = I\left[i\sigma_1 + \sigma_2\right]k_B \ln\left(x_1/x_2\right) \tag{A.10b}$$

We notice that the argument of the logarithm exhibited in the overall entropy spinor is now dimensionless, as is conventionally appropriate.

For the case where the holomorphic trajectory Σ represents a double helix configuration (with axis along the γ_3 direction with co-ordinate x_3), we define the co-ordinates along the γ_1 and γ_2 directions as $x_1 = R\exp\left(i\kappa x_3\right)$ and $x_2 = -iR\exp\left(i\kappa x_3\right)$ so that x_1 and x_2 each represent a mutually orthogonal holomorphic plane wave, i.e. the geometric interpretation here is that each plane wave represents a different (orthogonal) polarisation. In particular, differentiating each with respect to x_3 (indicated by the prime symbol) we find that the x_1 and x_2 co-ordinates are related to each other via a pair of cross-coupled equations: $x_1' = -\kappa x_2$ and $x_2' = \kappa x_1$. The coupling parameter is given by $\kappa = 2\pi/\lambda$, where λ is the helical pitch along the γ_3-axis. Substituting in for x_2 into (A.10), we can define the "spin down" entropy spinor using just x_1 and its derivative:

$$\left|f_{\Uparrow\downarrow}\right\rangle_1 = \left(1+i\right)k_B \ln\frac{\kappa x_1}{-x_1'} \tag{A.11a}$$

Alternatively, we can also define the entropy spinor using just x_2 and its derivative:

$$\left|f_{\Uparrow\downarrow}\right\rangle_2 = \left(1+i\right)k_B \ln\frac{x_2'}{\kappa x_2} \tag{A.11b}$$

These two entropy expressions (A.11) are independent of each other, since each represents a different co-ordinate direction. Thus we can linearly sum them, to create an overall spinor entropy expression, depending only on the holomorphic plane waves in space. In addition, we drop the bra-ket notation, as well as the reminder of its intrinsic "spin-down" aspect, and also take the entropy as the real part of Eq.A.11b, i.e. the entropy is the real part of $f = s + Ih$, to write the local entropy as:

$$s = k_B \ln\left(\frac{\kappa x^n}{x_n'}\right) \qquad \text{summation convention, } n \in \{1,2\} \tag{A.12}$$

Chirality Revealed

Writing the free-space Maxwell equations $\nabla F = 0$, with $F = (E + \underline{i}cB)\gamma_0$, in the Clifford algebra, where $\underline{i}$ is the 1+3 pseudoscalar as above ($\underline{i}^2 = -1$), we have

$$\nabla^j F = \nabla^j(E^k\gamma_k\gamma_0 - cB^k\gamma_k\gamma_1\gamma_2\gamma_3) = 0 \quad \text{summation convention, } k \in \{1,2,3\} \tag{A.13}$$

where $j \in \{0,1,2,3\}$; similarly, writing the complex info-entropy vector equations $\nabla f = 0$, with $f = s + Ih$, in the Clifford algebra, we have

$$\nabla^j f = \nabla^j(s^k\gamma_k\gamma_0 - h^k\gamma_k\gamma_1\gamma_2\gamma_3) = 0 \quad \text{summation convention, } k \in \{1,2,3\} \tag{A.14}$$

that is, following Denker [9]

$$\nabla f = \begin{matrix} +\nabla^0 s^1\gamma_1 & +\nabla^1 s^1\gamma_1 & -\nabla^2 s^1\gamma_0\gamma_1\gamma_2 & +\nabla^3 s^1\gamma_0\gamma_3\gamma_1 \\ +\nabla^0 s^2\gamma_2 & +\nabla^1 s^2\gamma_0\gamma_1\gamma_2 & +\nabla^2 s^2\gamma_0 & -\nabla^3 s^2\gamma_0\gamma_2\gamma_3 \\ +\nabla^0 s^3\gamma_3 & -\nabla^1 s^3\gamma_0\gamma_3\gamma_1 & +\nabla^2 s^3\gamma_0\gamma_2\gamma_3 & +\nabla^3 s^3\gamma_0 \\ -\nabla^0 h^1\gamma_0\gamma_2\gamma_3 & -\nabla^1 h^1\gamma_1\gamma_2\gamma_3 & -\nabla^2 h^1\gamma_3 & +\nabla^3 h^1\gamma_2 \\ -\nabla^0 h^2\gamma_0\gamma_3\gamma_1 & +\nabla^1 h^2\gamma_3 & -\nabla^2 h^2\gamma_1\gamma_2\gamma_3 & -\nabla^3 h^2\gamma_1 \\ -\nabla^0 h^3\gamma_0\gamma_1\gamma_2 & -\nabla^1 h^3\gamma_2 & +\nabla^2 h^3\gamma_1 & -\nabla^3 h^3\gamma_1\gamma_2\gamma_3 \end{matrix} = 0 \tag{A.15}$$

For the transverse right-handed double-helical structures of interest, we have $s^3 = h^3 = 0$, and $h^1 = R\cos\kappa x_3$ and $h^2 = R\sin\kappa x_3$ so that the ∇^1 and ∇^2 terms are also zero (∇^1 is $\partial/\partial x_1$ *etc.*). So Eq. A.15 for $\nabla f = 0$ reduces to the pair of equations

$$(\nabla^0 s^1 - \nabla^3 h^2)\gamma_1 + (\nabla^0 s^2 + \nabla^3 h^1)\gamma_2 = 0 \tag{A.16a}$$

and therefore $\nabla^0 s^1 = \nabla^3 h^2$ and $\nabla^0 s^2 = -\nabla^3 h^1$. That is:

$$\mathrm{d}s^1/\mathrm{dt} = \mathrm{d}h^2/\mathrm{d}x_3 = \kappa R\cos\kappa x_3 = \kappa h^1 \tag{A.16b}$$

$$\mathrm{d}s^2/\mathrm{dt} = -\mathrm{d}h^1/\mathrm{d}x_3 = \kappa R\sin\kappa x_3 = \kappa\, h^2 \tag{A.16c}$$

Thus, $\mathrm{d/dt}(s^1 + s^2) = \kappa(h^1 + h^2)$; that is:

$$\mathrm{d}\boldsymbol{s}/\mathrm{dt} = \kappa\boldsymbol{h} \tag{A.17}$$

For transverse *left*-handed double-helical structures, we have $h^1 = R\cos\kappa x_3$ and $h^2 = -R\sin\kappa x_3$, and Eq.A.17 becomes

$$\mathrm{d}\boldsymbol{s}/\mathrm{dt} = -\kappa\boldsymbol{h} \tag{A.18}$$

Thus, for a right-handed double helix the local rate of change of entropy s must be positively aligned with the information vector h whereas for a left-handed double helix it must be negatively aligned. If the structure has information then $\boldsymbol{h} > 0$ and only the right-handed structure is consistent with the 2nd Law.

For photons in free space both the entropy and the information vanish (since L in Eq.D.4 is a relativistic length which must vanish for particles travelling at the speed of light), so that both left-handed and right-handed polarisations are equally likely. Maxwell's equations are not chiral: Faraday's "Right-Hand Rule" depends on the definition of the curl operator, but in the Clifford algebra the lack of chirality is explicit. Thus the 2nd Law brings in the asymmetry with respect to *time*, which the correct Clifford algebra treatment of the *entropy* as a quantity in Minkowski space-time elegantly formalises.

To emphasise this important point another way: photons, being massless particles, have a zero time metric (they do not experience time); therefore they are not affected by the 2nd Law and may equally have right- or left-polarisation. However,

being massive, DNA does have a time metric, is subject to the 2nd Law, and is expected to be right-handed. The observation of left-handed Z-DNA is not a counter-example of this conclusion because this "... *conformation ... does not exist as a stable feature of the double helix. Instead, it is a transient structure that is occasionally induced by biological activity and then quickly disappears*" [11].

In his review, Podlech (2001) [12] considers that "*biomolecular homochirality seems to be a prerequisite for the creation of life*" , which is widely recognised as one of the "*unanswered questions of nature concerning the origin of life*" [13]. The fact that "unnatural" left-handed DNA can be synthesised [14] is interesting, but Nature certainly prefers a right-handed chirality, and we have possibly identified a fundamental physical reason for this preference.

References

[1] M.C. Parker, S.D. Walker, "A dynamic model of information and entropy", *Entropy*, 12, p80-88, 2010

[2] N. Gershenfeld, The Physics of Information Technology; Cambridge University Press: Cambridge, UK, 2000; Chapter 4.

[3] S.K. Pandey, R.S. Chakravarti, "The Dirac Equation: An approach through Geometric Algebra", *Annales de la Fondation Louis de Broglie*, Vol.34(2), pp.223-228, 2009

[4] M.R.Francis, A.Kosowsky, "The Construction of Spinors in Geometric Algebra", *Annals of Physics*, 317, pp383-409, 2005

[5] D. Hestenes, "Oersted Medal Lecture 2002: Reforming the Mathematical Language of Physics", 2002

[6] M.C. Parker, S.D. Walker, 'A Dynamic Model of Information and Entropy', *Entropy*, **12** (2010) 80-88

[7] I. Bialynicki-Birula, "Photon Wave Function", *Progress in Optics XXXVI* pp. 245-294 (Editor E. Wolf, Elsevier, Amsterdam, 1996), arXiv:quant-ph/0508202

[8] A.J.S. Hamilton, "*General Relativity, Black Holes, and Cosmology*", Section 14.4, page 336, 2005, https://jila.colorado.edu/~ajsh/astr3740_17/grbook.pdf (downloaded 27th September 2018)

[9] John S. Denker, *Electromagnetism using Geometric Algebra versus Components*, 2008 (https://www.av8n.com/physics/maxwell-ga.pdf; downloaded 26th June 2018)

[10] R.E.A.C.Paley, N.Wiener, Fourier transforms in the complex domain (American Mathematical Society Colloquium) *Am.Math.Soc.C.Public* **19** (1934)

[11] A.Rich, S.Zhang, Z-DNA: the long road to biological function, *Nature Genetics* **4** (2003) 566-572

[12] J. Podlech, Origin of organic molecules and biomolecular homochirality, *Cellular & Molecular Life Sciences* 2001, **58**, 44–60

[13] S. Negi, M. Dhanasekaran, T. Hirata, H. Urata, Y. Sugiura, Biomolecular Mirror-Image Recognition: Reciprocal Chiral-Specific DNA Binding of Synthetic Enantiomers of Zinc Finger Domain from GAGA Factor, *Chirality* 2006, **18**:254–258

[14] Hidehito Urata, Emiko Ogura, Keiko Shinohara, Yoshiaki Ueda and Masao Akagi, Synthesis and properties of mirror-image DNA, *Nucleic Acids Research*, 1992, **20**, 3325-3332

Annex - Derivation of Equation Eq.A2

We wish to perform the integration:

$$h_n = k_B \int_{-\infty}^{\infty} \left(\rho_n \ln \rho_n\right) c\,dt\,\gamma_0 \qquad \text{(AA.1a)}$$

and prove that Eq.A.2 is correct, that is,

$$h_n = k_B \left[\oint \rho_n \ln \rho_n \, dx_{0n} \right] = k_B \ln\left(x_n\right) \gamma_n \gamma_0 \qquad \text{(AA.1b)}$$

Such a calculation requires the transformation from a line integral of Eq.(AA.1a) into a contour integration (AA.1b), as indicated above, which therefore forms a key step to this analysis. For a causal and stable function such as $\rho_n\left(ct\right)$, that obeys the Paley-Wiener condition – that is, it is square-integrable (see below) and causal in nature – and whose denominator is a Hurwitz polynomial (that is, it is a stable function), means that we can employ Jordan's Lemma, whereby the curved section Ω_C of the contour integral with infinite radius can be assumed to not contribute to the final

result. In which case, we therefore have a direct equality between the line integral along the real axis and the closed contour integral across the complex plane, i.e. as indicated in the Figure AA1. This assumes a real (i.e. physically realizable), stable and causal function for $\rho_n(ct)$, which is indeed (unconditionally) the case for the kind of information-bearing (i.e. entropic) and physical structures that we are interested in. Our analysis therefore does not apply to physically implausible functions or geometries. Such a restriction does not undermine the application of our theory to the physical world; rather it reinforces the fact that our theory is truly only a "real world" physical analysis.

Consider the time-domain function $\varphi_n(ct)=\sqrt{\frac{x_n\gamma_n}{\pi}}\frac{1}{ct\gamma_0-x_{\text{pole }n}}$, which is a function featuring a pole at $x_{\text{pole }n}=ct_0\gamma_0+x_n\gamma_n$. Here, we employ the Clifford algebra, such that the axes of the complex plane are given by γ_0 (i.e. real axis) with $\gamma_0^2=+1$, and γ_n (i.e. an imaginary axis) where $\gamma_n^2=-1$. The "amplitude-squared" of the function $\varphi_n(ct)$ is given by $\rho_n=\varphi_n(ct)\varphi_n^*(ct)$.

Calculation of Square-Integrability of $\varphi_n(ct)$

As a pre-cursor to calculating Eq.AA1, we first calculate the square-integrability of $\rho_n(ct)$ as follows, and learn some useful rules in so doing:

$$\int_{-\infty}^{\infty}\rho_n\gamma_0 cdt=\int_{-\infty}^{\infty}\varphi_n\varphi_n^*\gamma_0 cdt=\frac{x_n\gamma_n}{\pi}\int_{-\infty}^{\infty}\frac{1}{ct\gamma_0-(ct_0\gamma_0+x_n\gamma_n)}\cdot\frac{1}{ct\gamma_0-(ct_0\gamma_0-x_n\gamma_n)}\gamma_0 cdt$$
$$=\frac{x_n\gamma_n}{\pi}\int_{-\infty}^{\infty}\frac{\gamma_0 cdt}{(ct-ct_0)^2+x_n^2} \qquad \text{(AA.2)}$$

Eq.(AA.2) is therefore just a Lorentzian function, which is normalised via the parameter x_n, which is the location of the pole at $x_{\text{pole}n}$ along the imaginary γ_n axis. Equation (AA.2) can be straightforwardly proven by a contour integral in the complex $x_{0n}=ct\gamma_0+\chi_n\gamma_n$ plane, by analytically continuing $\varphi_n(ct)$ from the time domain into the complex space-time plane: $\varphi_n(ct)\to\varphi_n(x_{0n})$. In which case, consider:

$$\int_{-\infty}^{\infty}\varphi_n(ct)\varphi_n^*(ct)\gamma_0 cdt\Rightarrow\oint\varphi_n(x_{0n})\varphi_n^*(x_{0n})dx_{0n}=\frac{x_n\gamma_n}{\pi}\oint\frac{1}{x_{0n}-x_{\text{pole }n}}\cdot\frac{1}{x_{0n}-x_{\text{pole }n}^*}dx_{0n} \qquad \text{(AA.3)}$$

Where the "straight" time-axis section h_n of the contour integral corresponds to equation Eq.(AA.2), and the semi-circular section Ω_C is across the complex plane, as indicated in the Figure AA1, below.

We can partial fractionate the argument of the contour integral in Eq.(AA.3) thus:

$$\frac{1}{x_{0n}-x_{\text{pole }n}}\cdot\frac{1}{x_{0n}-x_{\text{pole }n}^*}=\frac{1}{2x_n\gamma_n}\frac{1}{(x_{0n}-x_{\text{pole }n})}-\frac{1}{2x_n\gamma_n}\frac{1}{(x_{0n}-x_{\text{pole }n}^*)}$$
$$\Rightarrow\varphi_n\varphi_n^*=\frac{1}{2\pi}\frac{1}{(x_{0n}-x_{\text{pole }n})}-\frac{1}{2\pi}\frac{1}{(x_{0n}-x_{\text{pole }n}^*)} \qquad \text{(AA.4)}$$

Hence the contour integral of Eq.(AA.3) can be expressed as:

$$\frac{x_n\gamma_n}{\pi}\oint\frac{1}{x_{0n}-x_{\text{pole }n}}\cdot\frac{dx_{0n}}{x_{0n}-x_{\text{pole }n}^*}=\oint\frac{1}{2\pi}\frac{dx_{0n}}{(x_{0n}-x_{\text{pole }n})}-\frac{1}{2\pi}\frac{dx_{0n}}{(x_{0n}-x_{\text{pole }n}^*)}=2\pi\gamma_n\gamma_0\sum\mathcal{R} \qquad \text{(AA.5)}$$

where the quantity $\Sigma\mathcal{R}$ corresponds to the sum of the residues of the contour integral. However, the pole at $x^*_{\text{pole }n}$ is not enclosed by the contour integral, as indicated in the Figure AA1 below, and so contributes nothing to the integral. By contrast, the pole at $x_{\text{pole }n}$ is enclosed, and it has a residue of $\mathcal{R} = 1/2\pi$ (i.e., the coefficient of x_{0n}^{-1},) as can be seen from Eq.(AA.5).

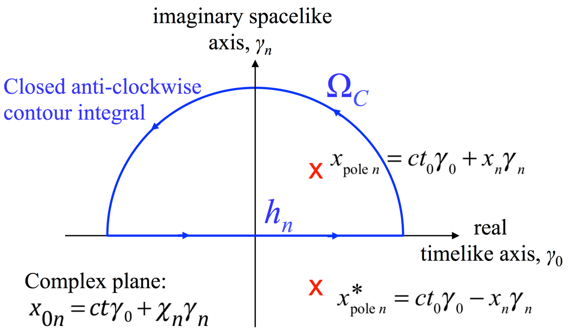

Figure AA1: Closed contour integral across complex x_{0n} plane.

Thus the integral of Eq.(AA.5) yields

$$2\pi\gamma_n\gamma_0\sum\mathcal{R} = 2\pi\gamma_n\gamma_0\cdot\frac{1}{2\pi} = \gamma_n\gamma_0$$

Using Jordan's Lemma, whereby the curved section Ω_C of the contour integral vanishes as its radius tends to infinity, means we can write:

$$\int_{-\infty}^{\infty}\rho_n(ct)\,\gamma_0 cdt = \int_{-\infty}^{\infty}\varphi_n(ct)\varphi_n^*(ct)\,\gamma_0 cdt = \frac{x_n\gamma_n}{\pi}\oint\frac{1}{x_{0n}-x_{\text{pole }n}}\cdot\frac{1}{x_{0n}-x^*_{\text{pole }n}}dx_{0n} = \gamma_n\gamma_0 \qquad \text{(AA.6)}$$

This proves the square-integrability of ρ_n.

Calculation of Information h_n

We now employ the results gained above to calculate the 'information' h_n, which is the quantity defined in Eq.A2. We again, analytically continue the function $\rho_n = \varphi_n(ct)\varphi_n^*(ct)$ into the complex space-time plane $x_{0n} = ct\gamma_0 + \chi_n\gamma_n$, so that (again using Jordan's Lemma) we can write the algebraic expression for the information as:

$$h_n = k_B\int_{-\infty}^{\infty}(\rho_n\ln\rho_n)\,cdt\,\gamma_0 = k_B\oint\varphi_n\varphi_n^*\ln(\varphi_n\varphi_n^*)\,dx_{0n} \qquad \text{(AA.7)}$$

We make use of the partial fraction result of Eq.(AA.4) to write:

$$h_n = k_B\frac{1}{2\pi}\oint\left(\frac{1}{(x_{0n}-x_{\text{pole }n})}-\frac{1}{(x_{0n}-x^*_{\text{pole }n})}\right)\left(\ln\left(\sqrt{\frac{x_n\gamma_n}{\pi}}\frac{1}{(x_{0n}-x_{\text{pole }n})}\right)+\ln\left(\sqrt{\frac{x_n\gamma_n}{\pi}}\frac{1}{(x_{0n}-x^*_{\text{pole }n})}\right)\right)dx_{0n} \qquad \text{AA.8a)}$$

The logarithms in equation Eq.(AA.8a) can be split apart to create a sum of 3 expressions:

$$h_n = k_B\frac{1}{2\pi}\oint\left(\frac{1}{(x_{0n}-x_{\text{pole }n})}-\frac{1}{(x_{0n}-x^*_{\text{pole }n})}\right)\left(\ln\left(\frac{x_n\gamma_n}{\pi}\right)-\ln(x_{0n}-x_{\text{pole }n})-\ln(x_{0n}-x^*_{\text{pole }n})\right)dx_{0n} \qquad \text{(AA.8b)}$$

These can then be multiplied out with the bracketed expression, to create a set of 5 different contour integrals:

$$h_n = h_{(i)} + h_{(ii)} + h_{(iii)} + h_{(iv)} + h_{(v)} \qquad \text{(AA.9)}$$

where the five integrals are as follows:

$$h_{(i)} = k_B \frac{1}{2\pi} \ln\left(\frac{x_n \gamma_n}{\pi}\right) \oint \left(\frac{1}{\left(x_{0n} - x_{\text{pole } n}\right)} - \frac{1}{\left(x_{0n} - x^*_{\text{pole } n}\right)} \right) dx_{0n} \qquad \text{(AA.9i)}$$

$$h_{(ii)} = -k_B \frac{1}{2\pi} \oint \frac{\ln\left(x_{0n} - x_{\text{pole } n}\right)}{\left(x_{0n} - x_{\text{pole } n}\right)} dx_{0n} \qquad \text{(AA.9ii)}$$

$$h_{(iii)} = +k_B \frac{1}{2\pi} \oint \frac{\ln\left(x_{0n} - x_{\text{pole } n}\right)}{\left(x_{0n} - x^*_{\text{pole } n}\right)} dx_{0n} \qquad \text{(AA.9iii)}$$

$$h_{(iv)} = -k_B \frac{1}{2\pi} \oint \frac{\ln\left(x_{0n} - x^*_{\text{pole } n}\right)}{\left(x_{0n} - x_{\text{pole } n}\right)} dx_{0n} \qquad \text{(AA.9iv)}$$

$$h_{(v)} = +k_B \frac{1}{2\pi} \oint \frac{\ln\left(x_{0n} - x^*_{\text{pole } n}\right)}{\left(x_{0n} - x^*_{\text{pole } n}\right)} dx_{0n} \qquad \text{(AA.9v)}$$

We consider the five contour integrals in turn, and implicitly employ Jordan's Lemma as required:

(i) First, we perform the contour integral of $h_{(i)}$ in the upper half of the complex plane (i.e., as per Figure AA1), such that it doesn't enclose the pole at $x^*_{\text{pole } n}$. In which case the residue at $x_{\text{pole } n}$ is $\mathcal{R}$=1, so that the integral becomes:

$$h_{(i)} = k_B \frac{1}{2\pi} \ln\left(\frac{x_n \gamma_n}{\pi}\right) \oint \left(\frac{1}{\left(x_{0n} - x_{\text{pole } n}\right)} - \frac{1}{\left(x_{0n} - x^*_{\text{pole } n}\right)} \right) dx_{0n} = k_B \frac{1}{2\pi} \ln\left(\frac{x_n \gamma_n}{\pi}\right) \cdot 2\pi \gamma_n \gamma_0 \cdot 1$$

$$h_{(i)} = k_B \ln\left(\frac{x_n \gamma_n}{\pi}\right) \gamma_n \gamma_0 \qquad \text{(AA.10i)}$$

The equation Eq.(AA.10i) is the key result for the proof of equation Eq.(AA.1).

(ii) To solve for $h_{(ii)}$ we revert back to the real axis, and integrate by parts:

$$\begin{aligned} h_{(ii)} &= -\frac{k_B}{2\pi} \oint \frac{\ln\left(x_{0n} - x_{\text{pole } n}\right)}{\left(x_{0n} - x_{\text{pole } n}\right)} dx_{0n} = -\frac{k_B}{2\pi} \int_{-\infty}^{\infty} \frac{u = \ln\left(ct\gamma_0 - x_{\text{pole } n}\right)}{v^{-1} = \left(ct\gamma_0 - x_{\text{pole } n}\right)} c\,dt\gamma_0 = -\frac{k_B}{2\pi} \left\{ u \int v - \int u' \int v \right\} \\ &= -\frac{k_B}{2\pi} \left\{ \left[\ln\left(ct\gamma_0 - x_{\text{pole } n}\right) \right]_{-\infty}^{\infty} \left[\ln\left(ct\gamma_0 - x_{\text{pole } n}\right) \right]_{-\infty}^{\infty} - \int_{-\infty}^{\infty} \frac{\ln\left(ct\gamma_0 - x_{\text{pole } n}\right)}{\left(ct\gamma_0 - x_{\text{pole } n}\right)} c\,dt\gamma_0 \right\} \\ &= -\frac{k_B}{2\pi} \left\{ \left[\ln\left(ct\gamma_0 - x_{\text{pole } n}\right) \right]_{-\infty}^{\infty} \left[\ln\left(ct\gamma_0 - x_{\text{pole } n}\right) \right]_{-\infty}^{\infty} \right\} - h_{(ii)} \\ \Rightarrow h_{(ii)} &= -\frac{1}{2} \frac{k_B}{2\pi} \left\{ \left[\ln\left(ct\gamma_0 - x_{\text{pole } n}\right) \right]_{-\infty}^{\infty} \right\}^2 \end{aligned} \qquad \text{(AA.10iia)}$$

To solve Eq.(AA.10iia) we substitute for $ct\gamma_0 = R$ and $ct\gamma_0 = Re^{i\pi}$ for the limits, so we can write:

$$\begin{aligned} h_{(ii)} &= -\frac{k_B}{4\pi} \left\{ \left[\ln\left(ct\gamma_0 - x_{\text{pole } n}\right) \right]_{-\infty}^{\infty} \right\}^2 = -\frac{k_B}{4\pi} \left\{ \ln\left(R - x_{\text{pole } n}\right) - \ln\left(Re^{i\pi} - x_{\text{pole } n}\right) \right\}^2 \\ &= -\frac{k_B}{4\pi} \left\{ \ln R\left(1 - \tfrac{x_{\text{pole } n}}{R}\right) - \ln Re^{i\pi}\left(1 - \tfrac{x_{\text{pole } n}}{Re^{i\pi}}\right) \right\}^2 = -\frac{k_B}{4\pi} \left\{ \ln R + \ln\left(1 - \tfrac{x_{\text{pole } n}}{R}\right) - \ln R - \ln e^{i\pi} - \ln\left(1 - \tfrac{x_{\text{pole } n}}{Re^{i\pi}}\right) \right\}^2 \end{aligned} \qquad \text{(AA.10iib)}$$

We let $R \to \infty$, assume that $R >> x_{\text{pole } n}$, and that ln1 = 0, such that we can simply make Eq.(AA.10iib) equal to:

$$h_{(ii)} = -\frac{k_B}{4\pi}\left\{\ln R - \ln R - \ln e^{i\pi}\right\}^2 = -\frac{k_B}{4\pi}\left(-i\pi\right)^2 = \frac{\pi k_B}{4} \qquad \text{(AA.10iic)}$$

(iii) To solve for $h_{(iii)}$ we consider a contour in the lower half of the complex plane, with a pole at $x^*_{\text{pole }n}$, hence:

$$\begin{aligned} h_{(iii)} &= \frac{k_B}{2\pi}\oint\frac{\ln\left(x_{0n}-x_{\text{pole }n}\right)}{\left(x_{0n}-x^*_{\text{pole }n}\right)}dx_{0n} = \frac{k_B}{2\pi}\left\{2\pi\gamma_n\gamma_0\sum\mathcal{R}\right\} = \frac{k_B}{2\pi}\cdot 2\pi\gamma_n\gamma_0\cdot\ln\left(x^*_{\text{pole }n}-x_{\text{pole }n}\right) \\ h_{(iii)} &= k_B\ln\left(x^*_{\text{pole }n}-x_{\text{pole }n}\right)\gamma_n\gamma_0 \end{aligned} \qquad \text{(AA.10iii)}$$

(iv) To solve for $h_{(iv)}$ we consider a contour in the upper half of the complex plane where there is a pole at $x_{\text{pole }n}$:

$$\begin{aligned} h_{(iv)} &= -\frac{k_B}{2\pi}\oint\frac{\ln\left(x_{0n}-x^*_{\text{pole }n}\right)}{\left(x_{0n}-x_{\text{pole }n}\right)}dx_{0n} = -\frac{k_B}{2\pi}\left\{2\pi\gamma_n\gamma_0\sum\mathcal{R}\right\} = -\frac{k_B}{2\pi}\cdot 2\pi\gamma_n\gamma_0\cdot\ln\left(x_{\text{pole }n}-x^*_{\text{pole }n}\right) \\ h_{(iv)} &= -k_B\ln\left(x_{\text{pole }n}-x^*_{\text{pole }n}\right)\gamma_n\gamma_0 \end{aligned} \qquad \text{(AA.10iv)}$$

(v) Finally, we consider $h_{(v)}$, where we again revert back to the real axis, as for the case of $h_{(ii)}$, and integrate by parts, and find again that the final result is essentially independent of the position of the pole, such that we can immediate write:

$$h_{(v)} = -\frac{\pi k_B}{4} \qquad \text{(AA.10v)}$$

where we note the simple change of sign as compared with $h_{(ii)}$, i.e. c.f. Eqs.(AA.9ii) & (AA.9v).

(Sum i…v) Thus, summing all the five integral results we find:

$$\begin{aligned} h_n &= k_B\ln\left(\frac{x_n\gamma_n}{\pi}\right)\gamma_n\gamma_0 + \frac{\pi k_B}{4} + k_B\ln\left(x^*_{\text{pole }n}-x_{\text{pole }n}\right)\gamma_n\gamma_0 - k_B\ln\left(x_{\text{pole }n}-x^*_{\text{pole }n}\right)\gamma_n\gamma_0 - \frac{\pi k_B}{4} \\ &= k_B\ln\left(\frac{x_n\gamma_n}{\pi}\right)\gamma_n\gamma_0 + k_B\ln\left(\frac{x^*_{\text{pole }n}-x_{\text{pole }n}}{x_{\text{pole }n}-x^*_{\text{pole }n}}\right) = k_B\ln\left(\frac{x_n\gamma_n}{\pi}\right)\gamma_n\gamma_0 + k_B\ln\left(\frac{-2x_n\gamma_n}{2x_n\gamma_n}\right)\gamma_n\gamma_0 \\ &= k_B\ln\left(\frac{x_n\gamma_n}{\pi}\right)\gamma_n\gamma_0 + k_B\ln\left(e^{\pm i\pi}\right)\gamma_n\gamma_0 = k_B\ln\left(\frac{x_n\gamma_n}{\pi}\right)\gamma_n\gamma_0 \pm i\pi k_B\gamma_n\gamma_0 \end{aligned} \qquad \text{(AA.11)}$$

Since h_n is essentially an integral quantity, we ignore the 'constant' aspects of the result obtained in Eq.(AA.11), both because of the indeterminate sign of the imaginary constant quantity and the constants being indeed just a constant of the integration, but more importantly also because we subsequently use the information h_n as a differential quantity in a dynamic calculus, where the constant aspects are simply differentiated away. Thus we can write:

$$h_n = k_B\int_{-\infty}^{\infty}\left(\rho_n\ln\rho_n\right)cdt\,\gamma_0 = k_B\ln\left(x_n\right)\gamma_n\gamma_0 \qquad \text{(AA.12)}$$

as per the original equation Eq.(AA.1). ***QED***

Appendix B

Appendix B: Entropic Hamiltonian & Lagrangian Analysis

Abstract

Trajectory equations in hyperbolic space are set up for the double-helix and the entropic analogue to the kinetic energy (that is, the *kinetic entropy*) derived in this case. The double-helix is analogous to a structure in free space so that the entropic potential is zero, and the canonical relations for the entropic Lagrangian and Hamiltonian are confirmed. We also confirm directly in this case that both the entropic momentum and the entropic Hamiltonian are conserved.

The same treatment is applied to the logarithmic double spiral. In this case the entropic potential is calculated: it does not vanish, and its behaviour is shown to be analogous in certain ways to that of a gravitational potential. Again, we confirm directly that both the entropic momentum and the entropic Hamiltonian are conserved. The Lagrangian in this case is given explicitly in Appendix C, which also explicitly confirms that the Euler-Lagrange equations in hyperbolic space are satisfied for both the logarithmic double spiral and the double helix (where the latter is shown to be a special case of the former).

In the Annex we demonstrate that the double-helix and the double logarithmic spiral are indeed holomorphic functions, that is, that the appropriate hyperbolic Cauchy-Riemann equations are satisfied.

Introduction

We perform an entropy-based analysis, in analogy to the conventional energy-based (kinematic) treatments based upon the Lagrangian and Hamiltonian approach. In particular, in our analysis, the entropic Lagrangian $L_S(q,q',x_3)$ is defined in hyperbolic 3-space q, and its variation is performed with respect to the Euclidean x_3 spatial parameter. This is in contrast to the conventional energetic approach, where the kinematic Lagrangian L is defined in Euclidean 3-space x, and varied according to the time parameter t. Thus, we therefore also note the key differentiating parameter x_3 in our entropic approach is defined in Euclidean space, and is distinct from its hyperbolic counterpart q_3. Indeed, in the entropic system, the x_3 axis can therefore often be seen to play a role that has similarities closer to the time axis than a conventional space dimension; the x_3 and $x_0 \equiv ct$ co-ordinates indeed being conjugates of each other in the Pauli algebra (see Appendix A). Thus in our analysis, we define the entropic Lagrangian equations of the space trajectories in analogy to the conventional equations of motion. Liouville's Theorem clearly applies (we shall return to this in further work), and we can consider the differential to be performed with respect to a surface normal, since in a 3D holographic system it is a 2D surface that defines the characteristics of the system.

The Double Helix

Trajectory Equations

For the holomorphic twin-trajectory geometries we are interested in (e.g. double helix of a photon, or of DNA etc.) the Euclidean co-ordinates of the double-helix (holomorphic) locus are given by:

$$x_1 = R_1 e^{i\kappa_0 x_3} \tag{B.1a}$$

$$x_2 = -iR_2 e^{i\kappa_0 x_3} \tag{B.1b}$$

$$x_3 \tag{B.1c}$$

We make the distinction between the radii in Eq.B.1a and Eq.B.1b, R_1 and R_2, respectively, which can be different from each other. But for a circular double helix we have $R_1 = R_2 = R_0$. The x_1 and x_2 co-ordinates manifest their holomorphic quality via their dependence on complex exponentials, i.e. each x_1 and x_2 co-ordinate representing an orthogonal polarised (e.g. linear, in the direction of the respective co-ordinate axis) plane wave travelling at constant velocity in the γ_3 direction. The x_3 co-ordinate itself is not holomorphic *per se*, and simply represents itself as the appropriate Euclidean co-ordinate quantity. However, we can transform from the conventional Euclidean space into the appropriate hyperbolic space by defining the position variable q, for the two co-ordinates x_1 and x_2:

Hyperbolic co-ordinate, q:
$$q_n = R_n \ln\left(\frac{x_n}{R_n}\right) \qquad n=1,2 \tag{B.2}$$

From which, by substituting as appropriate for x_1 and x_2, we can immediately write:

$$q_1 = iR_1\kappa_0 x_3 \tag{B.3a}$$

$$q_2 = iR_2\left(\kappa_0 x_3 - \frac{\pi}{2}\right) \tag{B.3b}$$

$$q_3 \equiv x_3 \tag{B.3c}$$

where Eq.B.3c is included for generality: see the comparable Eq.B.25c, and Eq.B.10 which must include all three spatial directions.

The hyperbolic co-ordinates q are linear functions of x_3, with q_1 and q_2 being imaginary (with a $\pi/2$ phase difference w.r.t. each other), while q_3 is real. Using Eq.B.2 we can also immediately define the first derivative w.r.t. x_3 for the three hyperbolic co-ordinate variables, to create a hyperbolic velocity term:

Hyperbolic (inverse) velocity, :
$$q'_n \equiv \frac{dq_n}{dx_3} = R_n\frac{x'_n}{x_n} \qquad n = 1,2,3 \tag{B.4}$$

From which we can write the three hyperbolic (inverse) velocities as:

$$q'_1 = iR_1\kappa_0 \tag{B.5a}$$

$$q'_2 = iR_2\kappa_0 \tag{B.5b}$$

$$q'_3 = 1 \tag{B.5c}$$

We note that for Eq.B.2, if we consider the Euclidean position x_n to be equal to $x_n = R_n + \Delta x_n$, i.e. the geometric position of interest is offset by the radius R_n, and further if $\Delta x_n << R_n$, then it is easy to show that the hyperbolic position q_n tends towards that of the Euclidean offset, $q_n \approx \Delta x_n$. Thus, for a very large holomorphic helix, and a geometric position located close to the radius of the helix, the Euclidean position and hyperbolic position are approximately equivalent; as are their derivatives. The hyperbolic position also thereby becomes independent of the normalising metric R_n.

We define the entropic momentum quantity p, as follows:

Entropic momentum, p:
$$p_n = \frac{i\kappa_0 k_B}{q'_n} \equiv \frac{m_S}{q'_n} \qquad n = 1,2,3 \tag{B.6a}$$

With this definition we shall see that the momentum p and position q variables satisfy the canonical equations of state, as well as the Euler-Lagrange equations for inflexions or stationary points as related to the principle of Least Exertion.

Eq.B.6a can be re-arranged to form an important identity relation, which appears particularly repeatedly in the Lagrangian analysis of the entropic system, as well as the Legendre transformation:

$$q'_n p_n = i\kappa_0 k_B \equiv m_S \qquad n = 1,2,3 \qquad \text{(B.6b)}$$

appears very frequently, playing a rôle analogous to that of mass. It is an "entropic mass" quantity of units [$JK^{-1}m^{-1}$], in contrast to conventional (inertial) mass which has units of [kg]. The subscript *"S"* used here and elsewhere signifies an entropic quantity.

The holomorphic double helix offers an interesting simple geometric interpretation of the entropic momentum. Substituting Eqs.B.5 for the inverse velocities into Eq.B.6a gives the nth coordinate p_n of the entropic momentum of a double helix as:

$$p_n = \frac{k_B}{R_n} \qquad n = 1,2,3 \qquad \text{(B.7)}$$

In this case, we can see that the transverse entropic momenta of a double helix are inversely proportional to the radius of curvature. Indeed, the simplicity of Eq.B.7 is dimensionally similar to the de Broglie definition of kinematic momentum in quantum mechanics $p = 2\pi\hbar/\lambda$, where $\hbar$ is the reduced Planck constant, and λ is the wavelength of the quantum system under consideration. Thus we see that in the entropic domain the Boltzmann constant k_B is isomorphic to the Planck constant $\hbar$.

Digression

As an aside, we note that we can make the derivation of Eq.B.3c consistent with Eqs.B.3a, B.3b by defining $x_3 \equiv R_3 e^{i\tilde{\kappa}_0 x_3}$ (which also makes Eq.B.1c more consistent in its form with Eqs.B.1a, B.1b) where the coupling coefficient is now negative imaginary and small, given by $\tilde{\kappa}_0 = -i/R_3$, and we assume that the radius tends to infinity $R_3 \to \infty$. Indeed, the geometric position of x_3 is therefore also understood to be offset by the radius R_3 (see the discussion after Eqs.B.5 above). Again, q_3 remains *qualitatively different* to q_1 and q_2, being real but now also having an imaginary (and very small) coupling coefficient $\tilde{\kappa}_0$ associated with it, that is $q_3 = i\tilde{\kappa}_0 R_3 x_3 = x_3$.

Using Eqs.B.2, B.4, we can also write the associated hyperbolic velocity $q'_3 = i\tilde{\kappa}_0 R_3$, such that we therefore also have $q'_3 = 1$ in agreement with Eq.B.5c. The associated entropic momentum term is therefore given by $p_3 = i\tilde{\kappa}_0 k_B / q'_3 = \tilde{m}_S$. This implies a different value (actually, also a real quantity) for the entropic mass in the γ_3 direction, $\tilde{m}_S = i\tilde{\kappa}_0 k_B = k_B / R_3$. Since in the following analyses the entropic mass represents a constant quantity in the differential equations of state, so that it tends to differentiate away, for simplicity and conciseness of notation we choose to make no distinction between $\tilde{m}_S$ and m_S in a lot of the subsequent analyses.

We recall the result of Appendix A, that in representing the double helix the bivector forms (each with a time-like edge) of the 4-space bases γ^n, $n \in \{0 \ldots 3\}$, are isomorphic to the 3-space Pauli spin matrices. In addition, the Pauli algebra identifies γ^0 (the time-like axis) to be conjugate to γ^3 (the propagation axis of the helix), and γ^1 and γ^2 are mutually conjugate and represent the transverse directions: analogues of the electric and magnetic fields in the EM wave. Therefore, our treatment of $n \in \{1,2\}$ holographically representing 3-space ($n \in \{1,2,3\}$), with the spatial coordinate

γ^3 being treated in the Lagrangian as analogous to time, is reasonable since the 3-space itself is derived as a quaternion sub-algebra from the contour integral with respect to time (γ^0) in Minkowski space-time (see Appendix A).

Kinetic & Potential Entropy

Having defined entropic momentum and velocity terms, we can now also define an associated equivalent kinetic entropy (KE) term. In particular, in conventional kinematics, the kinetic energy is given by the integration of the momentum.velocity product along the path:

$$T = \int p\,dv = \int mv\,dv = \tfrac{1}{2}mv^2 \tag{B.8a}$$

In the entropy case we can follow the same procedure to create an expression for the kinetic entropy T_S, using Eq.B.6a for the entropic momentum:

$$T_S = -\int p\,dv = -\int \frac{m_S}{q'}dq' = -m_S \ln q' = m_S \ln \frac{1}{q'} \tag{B.8b}$$

Due to the use of the inverse velocity, we need to employ an additional negative sign in the definition of the KE, to account for its reciprocal nature. As an aside, we can also see that the logarithmic nature of Eq.B.8b also allows us to naturally use the inverse velocity in the kinetic entropy expression such that the entropic mass term maintains its original sign. Of additional interest is that we can also use the rules of logarithms to modify Eq.B.8b so that it looks even more similar to the more conventional expression for the kinetic energy:

$$T_S = -m_S \ln q' = -\tfrac{1}{2} m_S \ln q'^2 \tag{B.8c}$$

Considering the three independent KE terms in the different axial directions, we therefore have:

$$T_S = -\tfrac{1}{2} m_S \ln q'_n q'^n \qquad \text{summation convention, } n = 1,2,3 \tag{B.9}$$

We can also employ Eq.B.6a to express the KE as a function of the entropic momentum p. In this case, simple substitution of $q'_n = m_S / p_n$ into Eq.B.9 gives:

$$T_S = \tfrac{1}{2} m_S \ln p_n p^n - 3m_S \ln m_S \qquad \text{summation convention, } n = 1,2,3 \tag{B.10}$$

Note the characteristic Shannon entropy metric appearing for the latter (constant) term in Eq.B.10. Considering the entropic equivalent to the potential energy, i.e. potential entropy (PE), this is conventionally just a function of position $V(x)$; hence we similarly assume that the potential entropy (PE) quantity is a function of hyperbolic position q, i.e. $V_S(q)$. In the conventional energy kinematics, the potential energy in a simple (uniform) gravitational field is given by $V=mgx$, where the gravitational acceleration is g [m/s^2], and x [m] is the distance moved by a point object of inertial mass m [kg] in the direction against the gravitational field. We can create an analogous (isomorphic) expression for the potential entropy (PE) quantity along the γ_3 axis of the geometry, using:

$$V_S = m_S \cdot \Gamma \cdot q \tag{B.11}$$

where the entropic acceleration Γ is given by:

$$\Gamma \equiv -\frac{q''}{q'^2} \tag{B.12}$$

and q is the hyperbolic distance moved in the direction against the entropic field acceleration Γ. Note that the entropic acceleration Γ is defined with a minus sign in Eq.B12 on account of q' being an inverse velocity. A more rigorous justification for Eq.B.11 is given in the sections §B.4 & §B.5 later in this Appendix B, where we consider conservation principles and the appropriate entropic Euler-Lagrange equation analogue to Newton's second law of motion.

Entropic Hamiltonian & Lagrangian Canonical Relations

Again, in analogy to conventional kinematics where the Hamiltonian is the total energy of the system, we define the entropic Hamiltonian H_S as the sum of the total kinetic entropy (KE) and potential entropy (PE) of the system:

$$H_S\left(q,p,x_3\right) = T_S + V_S = \sum_n^{1,2,3} -m_S \ln q_n' + V_S\left(q_n\right) \tag{B.13a}$$

In a canonical system, the entropic Lagrangian L_S is related to the entropic Hamiltonian H_S via the following Legendre transformation canonical relation, where we also take advantage of the identity Eq.B.6b:

$$L_S\left(q,p,x_3\right) = q_n' p^n - H_S = 3m_S - H_S \qquad \text{summation convention, } n = 1,2,3 \tag{B.14}$$

Whereas in the conventional kinematics differentials are with respect to time, in the entropic kinematics the differentiation is with respect to the Euclidean x_3 co-ordinate. As has been previously discussed, the γ_3 axis (although being a spatial dimension) has analogies to the time axis in kinematics. The canonical differential relationship between L_S and H_S can be quickly confirmed using Eq.B.14:

$$\frac{\partial L_S}{\partial x_3} = -\frac{\partial H_S}{\partial x_3} \tag{B.15}$$

To be appropriate (entropic) trajectory equations in space the usual canonical relations for the entropic Lagrangian must be satisfied, where $q' \equiv \mathrm{d}q/\mathrm{d}x_3$ (Eq.B.4) and similarly $p' \equiv \mathrm{d}p/\mathrm{d}x_3$; that is, the prime indicates the derivative w.r.t. the spatial x_3 co-ordinate which is taken as the entropic isomorph to the usual derivative w.r.t. time for kinematic systems:

$$p_n' = \frac{\partial L_S}{\partial q_n} \tag{B.16a}$$

$$q_n' = -\frac{\partial L_S}{\partial p_n} \tag{B.16b}$$

$$p_n = \frac{\partial L_S}{\partial q_n'} \tag{B.16c}$$

The canonical relations for the entropic Hamiltonian must also be satisfied:

$$p_n' = -\frac{\partial H_S}{\partial q_n} \tag{B.17a}$$

$$q_n' = \frac{\partial H_S}{\partial p_n} \tag{B.17b}$$

Canonical Relations for a Holomorphic Double Helix

We demonstrate here that the entropic Hamiltonian H_S and Lagrangian L_S for a holomorphic double helix structure satisfy all of the required canonical relations of Eqs.B.16, B.17, indicating that they are therefore canonically valid for describing an entropic trajectory in space. From Eqs.B.11, B.12, B.13, the entropic Hamiltonian is given by:

$$H_S = T_S + V_S = \sum_n^{1,2,3} \left(-\tfrac{1}{2} m_S \ln\left(q_n'^2\right) - m_S \frac{q_n''}{q_n'^2} q_n \right) \tag{B.18}$$

We can immediately see that for a holomorphic double helix there are no potential entropy terms, since from Eqs.B.5 the entropic velocities q' are constant such that $q'' = 0$. Hence, the Hamiltonian for a holomorphic double helix simply consists of a KE term, i.e. equivalent to a free moving particle in the absence of any fields. (This is also consistent with our earlier observation of the description of a holomorphic double helix as being that of two orthogonal plane waves moving at constant velocity in the γ_3 direction.) Thus for a double helix, the entropic Hamiltonian is given by:

$$H_S = -\tfrac{1}{2} m_S \ln\left(q_n' q'^n\right) = \tfrac{1}{2} m_S \ln\left(p_n p^n\right) - 3 m_S \ln m_S \tag{B.19a}$$

Where we have also used Eq.B.6a to express the KE also in terms of the momentum p. The relationship (B.17a) is trivially satisfied by noting that for a holomorphic double-helix the momentum is constant, $p_n = k_B / R_n$ according to Eq.B.7, so that we must have $p_n' = 0$; whilst the entropic Hamiltonian expression of Eq.B.19a contains no PE terms, such that $\partial H_S / \partial q_n = 0$. Hence Eq.B.17a is satisfied:

$$p_n' = -\frac{\partial H_S}{\partial q_n} = 0 \tag{B.20a}$$

By simple inspection of Eq.B.19a and again using Eq.B.6a, Eq. B.17b is also satisfied:

$$\frac{\partial H_S}{\partial p_n} = \frac{m_S}{p_n} = q_n' \tag{B.20b}$$

The equivalent canonical expressions for the entropic Lagrangian L_S are also easily seen to be satisfied by using Eq.B.14 and substituting for the entropic Hamiltonian of Eq.B.19a giving:

$$L_S = q_n' p^n - H_S = 3 m_S - \tfrac{1}{2} m_S \ln\left(p_n p^n\right) + 3 m_S \ln m_S \tag{B.21a}$$

Similarly, it is straightforward to show:

$$p_n' = \frac{\partial L_S}{\partial q_n} = 0 \tag{B.22a}$$

$$\frac{\partial L_S}{\partial p_n} = -\frac{m_S}{p_n} = -q_n' \tag{B.22b}$$

Finally, we can also re-write Eq.B.21a in terms of the entropic velocities q_n' , and then differentiate with respect to q_n' giving:

$$L_S = q_n' p^n - H_S = 3 m_S + \tfrac{1}{2} m_S \ln\left(q_n' q'^n\right) \tag{B.21b}$$

Differentiating with respect to q'_n we find agreement with equation (B.16c):

$$\frac{\partial L_S}{\partial q'_n} = \frac{m_S}{q'_n} = p_n \tag{B.22c}$$

confirming that the generalised momenta p_n are canonically conjugate to the q_n co-ordinates.

Conservation of Entropic Momentum and Entropic Hamiltonian

The fact that we have been able to construct an entropic Hamiltonian with associated Lagrangian canonical equations, which together also satisfy the Euler-Lagrange equations of stationary calculus (see Appendix C) means that according to Noether's theorem both the entropic Hamiltonian and also the entropic momentum are conserved, just as energy and momentum are conserved in kinematics.

The analysis of a holomorphic double helix is a trivial example of such entropic conservation laws, since the entropic momentum (Eq.B.7) is clearly constant for a fixed radius. In other words, a holomorphic double helix is analogous to a free particle travelling at constant velocity in the absence of any external fields, such that its momentum and KE remain constant. Indeed, substituting the expressions for the entropic velocities of equations (B.5) into the double helical entropic Hamiltonian of equation (B.19a), we see that the kinetic entropy and therefore the entropic Hamiltonian of a double helix is given by:

$$H_S = T_S = -\tfrac{1}{2} m_S \ln\left(q'_n q'^n\right) = -2m_S \ln\left(i\kappa_0 R_0\right) \tag{B.19b}$$

For constant radius R_0 and coupling coefficient κ_0, the entropic KE is clearly constant. For completeness, we also provide here the entropic momentum terms for a holomorphic double helix, using Eqs.B.5, B.6, B.7:

$$p_1 = \frac{m_S}{q'_1} = \frac{k_B}{R_0} \tag{B.23a}$$

$$p_2 = \frac{m_S}{q'_2} = \frac{k_B}{R_0} \tag{B.23b}$$

$$p_3 = \frac{\tilde{m}_S}{q'_3} = \tilde{m}_S = \frac{k_B}{R_3} \tag{B.23c}$$

All three momentum terms are constants for the entropic trajectory, and are therefore conserved along the path of the space trajectory. However, although the first two momentum terms are unambiguous in their geometric interpretation, the third momentum component p_3 has a less obvious interpretation since its associated radius (a real quantity) derived from $\tilde{m}_S = i\tilde{\kappa}_0 k_B$ and $R_3 \equiv 1/i\tilde{\kappa}_0 = \tilde{\lambda}/2\pi i$ is not obviously clear from the double helix geometry, since $\tilde{\lambda}$ is not to be identified with the helical pitch λ. However, it is clear that $p'_n = 0$ for all n=1,2,3, such that the momentum components are all conserved along the trajectory of the double helix.

Application to a Holomorphic Logarithmic Double Spiral

Trajectory Equations

We have studied the Hamiltonian and Lagrangian expressions for the fundamental (i.e. most basic) case of the holomorphic double helix, and derived the associated kinetic entropy T_S and entropic momentum p_n terms. However, we can now consider the more complex geometry of the logarithmic double spiral, and show how such a system can also obey the same entropic conservation laws and entropic equations of the trajectory in space.

In particular, we start with the holomorphic logarithmic double spiral in Euclidean co-ordinates:

$$x_1 = R_0 e^{-\Lambda x_3} e^{i\kappa x_3} \tag{B.24a}$$

$$x_2 = -i\, x_1 \tag{B.24b}$$

$$x_1 = R \exp(i\kappa x_3); \quad R \equiv R_0 \exp(-\Lambda x_3) \tag{B.24c}$$

Here, the radius of the spiral exponentially reduces with increasing distance x_3 along the axis; the rate of radius reduction being controlled by the logarithmic spiral parameter Λ. It is clear that when the logarithmic spiral parameter Λ=0, then the equations (B.24) revert back to the appropriate co-ordinate equations for a holomorphic double helix of radius R_0. We point out here that the coupling coefficient κ is assumed in the Eqs.B.24 here to vary in a similar fashion to the radius, that is

$$\kappa = \kappa_0 \exp\left(\Lambda x_3\right) \tag{B.24d}$$

The implications of this are discussed further in the sub-section on the "*Logarithmically varying coupling coefficient*" below, where we show how such an assumption actually leads to a simplified analysis; indeed that it makes a logarithmic double spiral in hyperbolic space completely isomorphic to that of a double helix in hyperbolic space. However, in the rest of this section §B.3 as well as the section §B.4 the coupling coefficient is assumed to be an unvarying constant κ_0.

We note a subtlety here, in that it is tempting to simply perform an apparently elegant one-to-one substitution for the coupling coefficient κ_0, by complexifying it and substituting it via $\kappa_0 \to \kappa_0 + i\Lambda$, particularly since κ_0 and Λ have the same dimensionality [m^{-1}] and appear to be the real and imaginary counterparts to each other. However, the coupling coefficient κ_0 represents a periodic quantity (i.e. the wavenumber related to the pitch of the spiral), whereas Λ does not represent such a periodic aspect. This is perhaps intrinsic due to their different complex properties; but whereas the coupling coefficient κ_0 can be exploited for useful Fourier functionalities (such as $\partial/\partial x_3 \equiv i\kappa_0$), a complexified κ_0 does not lend itself so easily to such Fourier application (that is, we can't assume such operators as $d/dx_3 \equiv i\kappa_0 + \Lambda$). In addition, by substituting for a complexified coupling coefficient κ_0, we start losing the clear geometrical interpretation of physical quantities, the momenta p or the positions q, which also have to be complexified. Instead, when the quantities κ_0 and Λ are kept unambiguously apart as in Eq.B.24, then the application of Eq. B.2 to Eq.B.24, yielding the hyperbolic co-ordinate variables q with the instantaneous (real) radius R_n of Eq.B.2 made equal to the logarithmic spiral quantity $R_n = R_0 e^{-\Lambda x_3}$ for (n=1,2), means the results are both more intuitive and more unambiguous.

Applying Eq.B.2 to Eqs.B.24 to obtain the hyperbolic co-ordinate variables q, with the instantaneous radius $R_n = R_0 e^{-\Lambda x_3}$ (for n=1,2), we find:

$$q_1 = iR_0 e^{-\Lambda x_3} \kappa_0 x_3 \tag{B.25a}$$

$$q_2 = iR_0 e^{-\Lambda x_3} \left(\kappa_0 x_3 - \frac{\pi}{2} \right) \tag{B.25b}$$

The n=3 hyperbolic co-ordinate can also be defined as:

$$q_3 = x_3 e^{-\Lambda x_3} \tag{B.25c}$$

Here, using a similar approach as that discussed in the Digression in §2 above, we again therefore assume $x_3 \equiv R_3 e^{i\tilde{\kappa}_0 x_3}$, however with the radius R_3 now also undergoing an equivalent exponential reduction from an initial radius $\tilde{R}_0$, so that $R_3 = \tilde{R}_0 e^{-\Lambda x_3}$ with the implication that $\tilde{\kappa}_0 = -i/\tilde{R}_0$. We calculate the entropic velocities as follows:

$$q_1' = iR_0 e^{-\Lambda x_3}\kappa_0\left(1-\Lambda x_3\right) \tag{B.26a}$$

$$q_2' = iR_0 e^{-\Lambda x_3}\kappa_0\left(1-\Lambda x_3 - \frac{\pi\Lambda}{2\kappa_0}\right) \tag{B.26b}$$

$$q_3' = e^{-\Lambda x_3}\left(1-\Lambda x_3\right) \tag{B.26c}$$

Clearly, for Λ=0 the entropic velocities revert to those for the holomorphic double helix of Eqs.B.5. Having defined the position and velocity variables for the logarithmic double spiral, we can now consider the conservation of the entropic momentum and the entropic Hamiltonian. We first provide an approximate overview of the interplay between the kinetic entropy (KE) and potential entropy (PE) as the quantities vary along the q_3 axis of the logarithmic double spiral, so as to provide an insight into how the subsequent results can be understood when we perform a more rigorous analysis based upon the Euler-Lagrange equations.

For our overview, we consider the direction along the q_3 axis. Using Eqs. B.26c, B.9, and B.13a the q_3-direction component of the entropic Hamiltonian is given by:

$$H_{S,q_3} = T_{S,q_3}\left(q_3'\right) + V_{S,q_3}\left(q_3\right) = -m_S \ln q_3' + V_{S,q_3} = -m_S\left(-\Lambda q_3 + \ln\left(1-\Lambda q_3\right)\right) + V_{S,q_3} \tag{B.27}$$

obtaining $q_3 \approx x_3$ from Eq.B.25c and assuming $\Lambda x_3 << 1$ (and hence, also, $\Lambda q_3 << 1$).

Since the total quantity as described by the entropic Hamiltonian is conserved, then as the logarithmic double spiral trajectory progresses a distance q along the q_3 axis from the location q_{3a} to the position $q_{3b} = q_{3a} + q$, then using Eq.B.27 we must have:

$$\begin{aligned} H_{S,q_3} &= -m_S\left(-\Lambda q_{3a} + \ln\left(1-\Lambda q_{3a}\right)\right) + V_S\left(q_{3a}\right) \\ &= -m_S\left(-\Lambda q_{3b} + \ln\left(1-\Lambda q_{3b}\right)\right) + V_S\left(q_{3b}\right) \end{aligned} \tag{B.28}$$

On the assumption that $\Lambda q_3 << 1$ we can employ the approximation $\ln\left(1-\Lambda q_3\right) \approx -\Lambda q_3$, from which we can see that the change in potential entropy is given by:

$$\Delta V_{S,q3} = V_S\left(q_{3b}\right) - V_S\left(q_{3a}\right) = -m_S 2\Lambda\left(q_{3b} - q_{3a}\right) = -m_S \cdot 2\Lambda \cdot q \tag{B.29a}$$

Thus, if the entropic Hamiltonian is constant along the trajectory line, the potential entropy (PE) must change by an amount linear to the distance moved along the γ_3-axis. In other words, the change in PE is analogous to the change in potential energy in a uniform gravitational (or accelerating) field: in this approximation the PE is simply proportional to the product of the entropic mass m_S, the distance moved q against the direction of the field, and the strength of the accelerating field. Then, from Eq.B.26c, the strength of the accelerating field is given by:

$$q_3'' = -2\Lambda e^{-\Lambda x_3}\left(1-\tfrac{1}{2}\Lambda x_3\right) \tag{B.30a}$$

On the assumption that $\Lambda x_3 << 1$ (that is, similar to $\Lambda q_3 << 1$, as in Eq.B.29a) we can approximate Eq.B.30a to show:

$$q_3'' \approx -2\Lambda \tag{B.30b}$$

From Eq.B26c, for $\Lambda x_3 << 1$ it is also clear that $q_3' \approx 1$, such that the entropic acceleration Γ_3 in the x_3 direction is closely given by:

$$\Gamma_3 = -\frac{q_3''}{q_3'^2} \approx 2\Lambda \tag{B.30c}$$

Thus the change in the potential entropy of Eq.B.29a can also be expressed as:

$$\Delta V_{S,q3} = -m_S \cdot \Gamma_3 \cdot q \tag{B.29b}$$

which is in agreement with Eq.B.11. The change in potential entropy is therefore analogous to the change in energy when moving in a uniform gravitational field, qualitatively supporting what was previously discussed in section §B.2 with respect to the initial description of potential entropy (PE).

The same qualitative analysis can also be performed for the q_1 and q_2 directions. However, we shall now instead proceed rigorously to a proof of these relations using the Euler-Lagrange equation.

Using the Eqs.B.26 for the entropic velocities and using Eq.B.6, the entropic momentum terms are:

$$p_1 = \frac{m_S}{q_1'} = \frac{k_B}{R_0 e^{-\Lambda x_3}\left(1-\Lambda x_3\right)} \tag{B.31a}$$

$$p_2 = \frac{m_S}{q_2'} = \frac{k_B}{R_0 e^{-\Lambda x_3}\left(1-\Lambda x_3 - \frac{\pi\Lambda}{2\kappa_0}\right)} \approx \frac{k_B}{R_0 e^{-\Lambda x_3}\left(1-\Lambda x_3\right)} \tag{B.31b}$$

$$p_3 = \frac{\tilde{m}_S}{q_3'} = \frac{\tilde{m}_S}{e^{-\Lambda x_3}\left(1-\Lambda x_3\right)} \tag{B.31c}$$

We can see how the entropic momentum terms p_1 and p_2 for the logarithmic double spiral conform to Eq.B.7 such that they are closely inversely proportional to the instantaneous (local) radius of curvature at the location q_n, and also exhibit the logarithmic spiral characteristic. However, in contrast to the case for the holomorphic double helix, all the momentum terms of Eqs.B.31 clearly contain an additional x_3 dependency, which means that they are changing along the trajectory of the logarithmic double spiral. In which case, they are clearly not being conserved; or rather, there is the presence of an *entropic force*, causing the momentum to change. Given that we have just calculated the entropic accelerating field, the existence of associated entropic forces should not be surprising.

Logarithmically varying coupling coefficient κ

We indicated (Eq.B.24d) that the coupling coefficient may vary logarithmically as $\kappa = \kappa_0 \exp\left(\Lambda x_3\right)$, such that the pitch of the logarithmic double-spiral therefore varies in sympathy as $\lambda = \lambda_0 \exp\left(-\Lambda x_3\right)$. The Euclidean co-ordinates of Eq.B.1 therefore become:

$$x_1 = R_1 e^{i\kappa_0 e^{\Lambda x_3} x_3} \tag{B.32a}$$

$$x_2 = -iR_2 e^{i\kappa_0 e^{\Lambda x_3} x_3} \tag{B.32b}$$

Employing the same equation Eq.B.2 to transform from the Euclidean into the hyperbolic space, means that the hyperbolic co-ordinate variables q, are now given by:

$$q_1 = R_0 e^{-\Lambda x_3}\left(i\kappa_0 e^{\Lambda x_3} x_3\right) = iR_0\kappa_0 x_3 \tag{B.33a}$$

$$q_2 = R_0 e^{-\Lambda x_3}\left(i\kappa_0 e^{\Lambda x_3} x_3 - i\frac{\pi}{2}\right) = iR_0\kappa_0 x_3 - i\frac{\pi}{2}R_0 e^{-\Lambda x_3} \tag{B.33b}$$

For the n=3 hyperbolic co-ordinate we again assume $x_3 \equiv R_3 e^{i\tilde{\kappa}_0 e^{\Lambda x_3} x_3}$, with the radius R_3 undergoing an equivalent exponential reduction from an initial radius $\tilde{R}_0$, so that $R_3 = \tilde{R}_0 e^{-\Lambda x_3}$; however, in this case we assume $\tilde{\kappa} = -i/R_3$, (indeed, $\tilde{\kappa}_0 = -i/\tilde{R}_0$) such that $\tilde{\kappa} = \tilde{\kappa}_0 e^{\Lambda x_3}$. Thus the n=3 hyperbolic co-ordinate is treated as:

$$q_3 = \tilde{R}_0 e^{-\Lambda x_3}\left(i\tilde{\kappa}_0 e^{\Lambda x_3} x_3\right) = i\tilde{R}_0\tilde{\kappa}_0 x_3 = x_3 \tag{B.33c}$$

It is also equally clear that the entropic velocities are therefore given by:

$$q_1' = iR_0\kappa_0 \tag{B.34a}$$

$$q_2' = iR_0\kappa_0 + i\frac{\pi}{2}\Lambda R_0 e^{-\Lambda x_3} \approx iR_0\kappa_0 \tag{B.34b}$$

$$q_3' = 1 \tag{B.34c}$$

If we assume that $0.5\pi\Lambda e^{-\Lambda x_3} << \kappa_0$ (as is the case for the Milky Way galactic structure) then we can make the approximation $q_2 \approx iR_0\kappa_0 x_3$ and $q_2' \approx iR_0\kappa_0$. In which case, the hyperbolic accelerations are therefore all zero, $q_n'' = 0$, indicating the effective absence of any entropic forces or any entropic potentials, V_S=0. Thus the entropic Hamiltonian in this case is only dependent on the kinetic entropy term T_S, as a function of the hyperbolic velocity and entropic mass. We continue to assume from Eqs.B.6a and B.6b that the entropic mass is given by $m_S = i\kappa_0 k_B$, and that for the n=3 hyperbolic co-ordinate case, we have $q_3' p_3 = i\tilde{\kappa}_0 k_B = \tilde{m}_S$. This means that the associated Hamiltonian H_S is a constant (that is, conserved) in hyperbolic space. In which case, it is clear that in hyperbolic space the entropic Hamiltonian of a logarithmic double spiral with logarithmically-varying coupling-coefficient $\kappa(x_3)$ is mathematically equivalent to that of a double helix of radius R_0 and pitch λ_0.

$$H_S = T_S = -\tfrac{1}{2} m_S \ln\left(q_n' q'^n\right) = -2m_S \ln\left(i\kappa_0 R_0\right) \tag{B.19c}$$

This means that the appropriate analyses demonstrating the conservation of the entropic momentum and the entropic Hamiltonian of the logarithmic double spiral now simply follow that of the double helix of section §B.2. However, in the following section, we continue to show how the logarithmic double spiral geometry still obeys these conservation laws in hyperbolic space, even when the coupling coefficient is assumed a constant of the geometry.

Entropic analogue to Newton's second law of motion

The Euler-Lagrange equation as applied in the entropic context is:

$$\frac{d}{dx_3}\frac{\partial L_S}{\partial q_n'} - \frac{\partial L_S}{\partial q_n} = 0 \tag{B.35}$$

For classical mechanics, such an Euler-Lagrange equation can be used to derive Newton's second law of motion ($F = m\ddot{x}$). We can use the same methodology to derive the equivalent entropic relationship. Using the Lagrangian of Eqs.B.14, B.13a and the entropic identity $q_n' p_n \equiv m_S$ from Eq.B.6a, we write the first term on the LHS of Eq.B.35 as follows:

$$\frac{d}{dx_3}\frac{\partial L_S}{\partial q_n'} = \frac{d}{dx_3}\frac{m_S}{q_n'} = \frac{dp_n}{dx_3} \tag{B.36a}$$

The classical kinematic analogue to the RHS of Eq.B.36a is the rate of change of momentum with time $\left(dp/dt = d(m\dot{x})/dt = m\ddot{x}\right)$. The second term on the LHS of Eq.B.35 can be considered as an equivalent to an entropic force: the negative gradient of the entropic potential. This is because the potential entropy (PE) component of the entropic Lagrangian contains the entropic position terms q_n.

$$\frac{\partial L_S}{\partial q_n} = \frac{d}{dx_3}\frac{\partial L_S}{\partial q_n'} = \frac{d}{dx_3}\left[\frac{m_S}{q_n'}\right] = -m_S\frac{q_n''}{q_n'^2} = m_S\Gamma_n \tag{B.36b}$$

The entropic acceleration (in hyperbolic space) is therefore given by Γ_n. The logarithmic double spiral offers a convenient system to show the properties of the entropic version of the Euler-Lagrange equation and how it acts as an entropic analogue to Newton's 2nd Law of motion. In the following, we employ the Euler-Lagrange equation to show how we can define the associated entropic potential fields V_S and entropic forces F_S associated with a double-armed logarithmic spiral, and how they therefore act together to create a consistent entropic system obeying the conservation of the sum of kinetic entropy and entropic potential.

Considering each of the three spatial components in turn:

q_1-coordinate

$$\frac{\partial L_S}{\partial q_1} = -\frac{\partial V_S}{\partial q_1} = -m_S\frac{q_1''}{q_1'^2} \tag{B.37a}$$

From Eq.B.26a we have:

$$q_1' = i\kappa_0 R_0 e^{-\Lambda x_3}\left(1-\Lambda x_3\right) \tag{B.26a}$$

so that we can write

$$q_1'' = -i\kappa_0 R_0 e^{-\Lambda x_3}\Lambda\left(2-\Lambda x_3\right) \tag{B.37b}$$

Such that

$$\Gamma_1 = -\frac{q_1''}{q_1'^2} = \frac{\Lambda}{i\kappa_0 R_0 e^{-\Lambda x_3}}\frac{\left(2-\Lambda x_3\right)}{\left(1-\Lambda x_3\right)^2} \tag{B.37c}$$

In which case, from Eq.B.37a, and reminding ourselves that the entropic potential $V_{S,q}$ here is defined in hyperbolic space q, we can write the entropic force in the q_1-direction as:

$$F_1 = -\frac{\partial V_{S,q1}}{\partial q_1} = m_S\Gamma_1 = -m_S\frac{q_1''}{q_1'^2} = \frac{m_S}{i\kappa_0 R_0}\frac{\Lambda\left(2-\Lambda x_3\right)e^{\Lambda x_3}}{\left(1-\Lambda x_3\right)^2} \tag{B.37d}$$

We need to integrate Eq.B.37d with respect to q_1 in order to find the q_1-dependency of the entropic potential V_S in hyperbolic space:

$$V_{S,q1} = m_S \int \frac{q_1''}{q_1'^2} dq_1 = m_S \int \frac{q_1''}{q_1'^2} q_1' dx_3 = m_S \int \frac{q_1''}{q_1'} dx_3 \tag{B.37e}$$

$$V_{S,q1} = -m_S \Lambda \int \frac{2-\Lambda x_3}{1-\Lambda x_3} dx_3 = -m_S \left[\Lambda x_3 - \ln\left(1-\Lambda x_3\right) \right] - m_S \ln K_1 \tag{B.37f}$$

This is the entropic field in hyperbolic space in the q_1-direction, and we can assume that the constant of integration is given (for convenience) by the composite quantity $-m_S \ln K_1$. However, the interpretation of Eq.B.37f isn't particularly obvious, since it is expressed in hyperbolic space, which isn't so intuitive. Alternatively, we can choose to express the entropic field in a Euclidean form $V_{S,x1}$, where the hyperbolic form of $V_{S,q1}$ can be related to its Euclidean version $V_{S,x1}$ by:

$$V_{S,q1} = -m_S \ln\left(\frac{-V_{S,x1}}{m_S/R_0} \right) \tag{B.37g}$$

This means that the entropic force F_1 in the γ_1 direction can also be expressed in a Euclidean format, so that it is given by:

$$F_1 = -\frac{\partial V_{S,q1}}{\partial q_1} = m_S \frac{\partial \ln\left(-\frac{R_0}{m_S} V_{S,x1}\right)}{\partial q_1} = \frac{m_S}{q_1'} \frac{\partial \ln\left(V_{S,x1}\right)}{\partial x_3} = \frac{m_S}{q_1' V_{S,x1}} \frac{\partial V_{S,x1}}{\partial x_3} \tag{B.37h}$$

Using Eqs.B.37g and B.37f we can assume by inspection that the form for $V_{S,x1}$ (that is, the entropic potential in Euclidean space) in the γ_1 direction as a function of x_3 is therefore as follows:

$$V_{S,x1} = \frac{-m_S K_1 e^{\Lambda x_3}}{R_0\left(1-\Lambda x_3\right)} \tag{B.37i}$$

where K_1 now appears as a constant multiplying factor. In addition, as used in the RHS of Eq.B.37h, it is also straightforward to demonstrate using Eqs.B.26a and B.37i that $q_1' V_{S,x1}/m_S = -i\kappa_0 K_1$, such that we can re-write Eq.B37h for the entropic force simply as:

$$F_1 = -\frac{\partial V_{S,q1}}{\partial q_1} = -\frac{1}{i\kappa_0 K_1} \frac{\partial V_{S,x1}}{\partial x_3} \tag{B.37j}$$

However, it should be noted that the RHS of Eq.37j, $\partial V_{S,x1}/\partial x_3$, although clearly a potential gradient does not represent the conventional form of a force: this being due to the derivative with respect to x_3 not being in the appropriate γ_1 direction. As such, although being proportional to and having the same direction of the entropic force F_1, the quantity $\partial V_{S,x1}/\partial x_3$ by itself cannot be considered as representing an entropic force, even though it is confusing that differentiating Eq.B.37i with respect to x_3 and including the factor $-i\kappa_0 K_1$ does indeed give the 'correct' result:

$$-\frac{1}{i\kappa_0 K_1} \frac{\partial V_{S,x1}}{\partial x_3} = \frac{m_S \Lambda \left(2-\Lambda x_3\right) e^{\Lambda x_3}}{i\kappa_0 R_0 \left(1-\Lambda x_3\right)^2} \tag{B.37k}$$

which is the same as Eq.B.37d. Rather, in considering the entropic potential in Euclidean space, it is more useful to recognise in Eq.B.37i the fact that it also implicitly includes $x_1 = R_0 e^{-\Lambda x_3} e^{i\kappa_0 x_3}$ within it, so that we can re-write it as:

$$V_{S,x1}\left(x_1\right) = -\frac{m_S K_1 e^{i\kappa_0 x_3}}{x_1\left(1-\Lambda x_3\right)} \tag{B.37l}$$

Thus the entropic field in the x_1 direction is found to be inversely proportional to the Euclidean space variable x_1. As a 'correctly' formed entropic force in Euclidean space F_{x1}, the gradient of V_S in the x_1 direction is therefore simply given by:

$$F_{x1} = -\frac{\partial V_{S,x1}}{\partial x_1} = -\frac{m_S K_1 e^{i\kappa_0 x_3}}{x_1^2\left(1-\Lambda x_3\right)} \tag{B.37m}$$

Here, we now see that the Euclidean entropic force in the x_1 direction appears to obey an inverse-square law with respect to the x_1 variable (that is with respect to the radius of the entropic locus about the x_3 axis) and is also seen to be proportional to the entropic mass m_S. It is therefore interesting to note that the interpretation of Eq.37f in hyperbolic space is not so obvious as its equivalent Euclidean form of Eq.B37l; in particular the appearance of an inverse-square law, such as seen in Eq.B37m. Hence, although the Euler-Lagrange equation is defined and satisfied in hyperbolic space, its most intuitive interpretation is actually to be found in Euclidean space.

q_2-coordinate

$$\frac{\partial L_S}{\partial q_2} = -\frac{\partial V_S}{\partial q_2} = -m_S \frac{q_2''}{q_2'^2} \tag{B.38a}$$

From Eq.B.26b we have:

$$q_2' = iR_0 e^{-\Lambda x_3}\kappa_0\left(1-\Lambda x_3 - \tfrac{\pi\Lambda}{2\kappa_0}\right) \tag{B.26b}$$

so that we can write

$$q_2'' = -i\Lambda R_0 e^{-\Lambda x_3}\kappa_0\left(2-\Lambda x_3 - \tfrac{\pi\Lambda}{2\kappa_0}\right) \tag{B.38b}$$

Such that

$$\Gamma_2 = -\frac{q_2''}{q_2'^2} = \frac{\Lambda}{i\kappa_0 R_0 e^{-\Lambda x_3}} \frac{\left(2-\Lambda x_3 - \pi\Lambda/2\kappa_0\right)}{\left(1-\Lambda x_3 - \pi\Lambda/2\kappa_0\right)^2} \tag{B.38c}$$

In which case, from Eq.B.38a we can write the entropic force in the q_2-direction as:

$$F_2 = -\frac{\partial V_{S,q2}}{\partial q_2} = m_S\Gamma_2 = -m_S\frac{q_2''}{q_2'^2} = \frac{m_S}{i\kappa_0 R_0}\frac{\Lambda\left(2-\Lambda x_3 - \pi\kappa/2\kappa_0\right)e^{\Lambda x_3}}{\left(1-\Lambda x_3 - \pi\kappa/2\kappa_0\right)^2} \tag{B.38d}$$

We integrate with respect to q_2 in order to find the q_2-dependency of V_S in hyperbolic space:

$$V_{S,q2} = m_S\int\frac{q_2''}{q_2'^2}dq_2 = m_S\int\frac{q_2''}{q_2'^2}q_2' dx_3 = m_S\int\frac{q_2''}{q_2'}dx_3 \tag{B.38e}$$

$$V_{S,q2} = -m_S\Lambda\int\frac{2-\Lambda x_3 - \pi\Lambda/2\kappa_0}{1-\Lambda x_3 - \pi\Lambda/2\kappa_0}dx_3 = -m_S\left[\Lambda x_3 - \ln\left(1-\Lambda x_3 - \pi\Lambda/2\kappa_0\right)\right] - m_S \ln K_2 \tag{B.38f}$$

This is the entropic field in hyperbolic space in the q_2-direction, and we can assume that the constant of integration is given (again for convenience) by the composite quantity $-m_S \ln K_2$. We can also choose again to express the entropic field in a Euclidean form $V_{S,x2}$, where the hyperbolic form of $V_{S,q2}$ is related to its Euclidean version $V_{S,x1}$ by:

$$V_{S,q2} = -m_S \ln\left(\frac{-V_{S,x2}}{m_S/R_0}\right) \tag{B.38g}$$

Again, this means that the entropic force F_2 in the γ_2 direction can also be expressed in a Euclidean format given by:

$$F_2 = -\frac{\partial V_{S,q2}}{\partial q_2} = m_S \frac{\partial \ln\left(-\frac{R_0}{m_S} V_{S,x2}\right)}{\partial q_2} = \frac{m_S}{q_2'} \frac{\partial \ln\left(V_{S,x2}\right)}{\partial x_3} = \frac{m_S}{q_1' V_{S,x2}} \frac{\partial V_{S,x2}}{\partial x_3} \tag{B.38h}$$

Using Eqs.B.38g and B.38f we can assume by inspection that the form for $V_{S,x2}$ (that is, the entropic potential in Euclidean space) in the γ_2 direction as a function of x_3 is therefore as follows:

$$V_{S,x2} = -\frac{m_S K_2 e^{\Lambda x_3}}{R_0\left(1 - \Lambda x_3 - \pi\Lambda/2\kappa_0\right)} \tag{B.38i}$$

As used in the RHS of Eq.B.38h, it is again straightforward to also demonstrate using Eqs.B.26b and B.38i that $q_2' V_{S,x2}/m_S = -i\kappa_0 K_2$, such that we can re-write Eq.B38h for the entropic force simply as:

$$F_2 = -\frac{\partial V_{S,q2}}{\partial q_2} = -\frac{1}{i\kappa_0 K_2} \frac{\partial V_{S,x2}}{\partial x_3} \tag{B.38j}$$

Again, it should be noted that the RHS of Eq.38j, $\partial V_{S,x2}/\partial x_3$, although clearly a potential gradient does not represent the conventional form of a force for the same reasons as given previously. Although being proportional to and having the same direction of the entropic force F_2, the quantity $\partial V_{S,x2}/\partial x_3$ cannot be considered as representing an entropic force, even though differentiating Eq.B.38i with respect to x_3 and including the factor $-i\kappa_0 K_2$ does indeed again give the 'correct' result:

$$-\frac{1}{i\kappa_0 K_2} \frac{\partial V_{S,x2}}{\partial x_3} = \frac{m_S}{i\kappa_0 R_0} \frac{\Lambda\left(2 - \Lambda x_3 - \pi\Lambda/2\kappa_0\right) e^{\Lambda x_3}}{\left(1 - \Lambda x_3 - \pi\Lambda/2\kappa_0\right)^2} \tag{B.38k}$$

which is the same as Eq.B.38d. Rather, in considering the entropic potential in Euclidean space, it is again more useful to recognise in Eq.B.38i the fact that it also implicitly includes $x_2 = -iR_0 e^{-\Lambda x_3} e^{i\kappa_0 x_3}$ within it, so that we can re-write it as:

$$V_{S,x2}\left(x_2\right) = \frac{i m_S K_2 e^{i\kappa_0 x_3}}{x_2\left(1 - \Lambda x_3 - \pi\Lambda/2\kappa_0\right)} \tag{B.38l}$$

We can see that for the case where $\Lambda << \kappa$ we can simplify Eq.B.38l and re-write it as:

$$V_{S,x2}\left(x_2\right) \approx \frac{i m_S K_2 e^{i\kappa_0 x_3}}{x_2\left(1 - \Lambda x_3\right)} \tag{B.38m}$$

Thus we see that in Euclidean space the entropic field in the γ_2-direction has the same form as that previously found for the γ_1 direction, but is imaginary; the two entropic fields together form a holomorphic pair. Again, we also find that the entropic field in the γ_2 direction is inversely proportional to the Euclidean space variable x_2, so that the gradient of V_S in the x_2 direction in Euclidean space is simply given by:

$$F_{x2} = -\frac{\partial V_{S,x2}}{\partial x_2} = \frac{i m_S K_2 e^{i\kappa_0 x_3}}{x_2^2\left(1-\Lambda x_3 - \pi\Lambda/2\kappa_0\right)} \approx \frac{i m_S K_2 e^{i\kappa_0 x_3}}{x_2^2\left(1-\Lambda x_3\right)} \tag{B.38n}$$

The entropic force in the γ_2 direction therefore also obeys an inverse-square law with respect to x_2 in Euclidean space, and is also seen to be proportional to the entropic mass m_S.

q_3-coordinate

$$\frac{\partial L_S}{\partial q_3} = -\frac{\partial V_S}{\partial q_3} = -\tilde{m}_S \frac{q_3''}{q_3'^2} \tag{B.39a}$$

From Eq.B.26c, $q_3' = e^{-\Lambda x_3}\left(1-\Lambda x_3\right)$, we can immediately write $q_3'' = -\Lambda\left(2-\Lambda x_3\right)e^{-\Lambda x_3}$, such that using both expressions, we have:

$$F_3 = -\frac{\partial V_{S,q3}}{\partial q_3} = \tilde{m}_S \Gamma_3 = -\tilde{m}_S \frac{q_3''}{q_3'^2} = \tilde{m}_S \frac{\Lambda\left(2-\Lambda x_3\right)e^{\Lambda x_3}}{\left(1-\Lambda x_3\right)^2} \tag{B.39b}$$

We integrate Eq.B.39b with respect to q_3 in order to gain the q_3-dependency of the entropic potential $V_{S,q3}$ in hyperbolic space:

$$V_{S,q3} = \tilde{m}_S \int \frac{q_3''}{q_3'^2} dq_3 = \tilde{m}_S \int \frac{q_3''}{q_3'^2} q_3' dx_3 = \tilde{m}_S \int \frac{q_3''}{q_3'} dx_3 \tag{B.39c}$$

$$V_{S,q3} = -\tilde{m}_S \Lambda \int \frac{2-\Lambda x_3}{1-\Lambda x_3} dx_3 = -\tilde{m}_S\left[\Lambda x_3 - \ln\left(1-\Lambda x_3\right)\right] - \tilde{m}_S \ln K_3 \tag{B.39d}$$

Thus the entropic field $V_{S,q3}$ in the γ_3-direction is a function in hyperbolic space, and we assume the constant of integration is given (again for convenience) by the quantity $-\tilde{m}_S \ln K_3$. We can express the entropic field in its Euclidean form $V_{S,x3}$, so that we can relate its hyperbolic form $V_{S,q3}$ to the Euclidean version $V_{S,x3}$ by:

$$V_{S,q3} = -\tilde{m}_S \ln\left(\frac{-V_{S,x3}}{\tilde{m}_S/R_3}\right) \tag{B.39e}$$

The entropic force F_3 in the γ_3 direction can then be expressed in a Euclidean format, given by:

$$F_3 = -\frac{\partial V_{S,q3}}{\partial q_3} = \tilde{m}_S \frac{\partial \ln\left(-\frac{R_3}{\tilde{m}_S} V_{S,x3}\right)}{\partial q_3} = \frac{\tilde{m}_S}{q_3'} \frac{\partial \ln\left(V_{S,x3}\right)}{\partial x_3} = \frac{\tilde{m}_S}{q_1' V_{S,x3}} \frac{\partial V_{S,x3}}{\partial x_3} \tag{B.39f}$$

By inspection, that is, comparing Eqs.B.39d and B.39e, we can therefore directly write the Euclidean version $V_{S,x3}$ as:

$$V_{S,x3}\left(x_3\right) = -\frac{\tilde{m}_S K_3 e^{\Lambda x_3}}{R_3\left(1-\Lambda x_3\right)} \tag{B.39g}$$

so that it also becomes clear that we can therefore assume $q_3' V_{S,x3} / \tilde{m}_S = -i\tilde{\kappa}_0 K_3$, remembering that $i\tilde{\kappa}_0 = 1/R_3$. In which case, it is immediately apparent from Eq.B39f that the entropic force in the γ_3 direction is equally given by:

$$F_3 = -\frac{\partial V_{S,q3}}{\partial q_3} = -\frac{1}{i\tilde{\kappa}_0 K_3}\frac{\partial V_{S,x3}}{\partial x_3} \tag{B.39h}$$

Thus, similar again to the q_1 and q_2 situations discussed earlier, the entropic force F_3 in the γ_3 direction is proportional to the potential gradient as expressed in Euclidean space; although the derivative with respect to x_3 is now in the correct (γ_3) direction. However, together, these differences emphasise again the fact that the Euclidean version of the potential gradient cannot, in general, be considered as representing an appropriate or 'correct' entropic force. It is the gradient of hyperbolic space that is required for the appropriate expression of the entropic force. That said, applying the RHS of Eq.B.39h to Eq.B.39g allows us to use the Euclidean form to derive the same expression for the entropic force F_3 as seen in Eq.B.39b.

For completeness we give the entropic Lagrangian for the double-armed logarithmic spiral in hyperbolic space from Eqs. B.37f, B.38f, B.39d:

$$L_S(q,q',x_3) = \sum_{n=1}^{3} q_n' p_n - H_S = 3m_S - (T_S + V_S) = 3m_S + \sum_{n=1}^{3} m_S \ln q_n' - V_S(q_n) \tag{B.40a}$$

Overall Entropic Hamiltonian of Holomorphic Logarithmic Double Spiral

Having defined all the various component contributions of both the KE and PE terms, we can now sum them together to define the overall entropic Hamiltonian of the logarithmic double spiral system in hyperbolic space. From Eq.B.13a we have:

$$H_S(q,q',x_3) = T_S + V_S = \sum_{n}^{1,2,3} -m_S \ln(q_n') + V_S(q_n) \tag{B.13b}$$

Substituting Eq.B.26 and Eqs.B.37f, B38f and B.39d into Eq.B.13b we can therefore write the following expression for the overall entropic Hamiltonian (where each line of the first part of Eq.B.40b corresponds to a different n=1,2,3 number):

$$\begin{aligned} H_S = &-m_S\left[\ln i\kappa_0 R_0 + \ln(1-\Lambda x_3) - \Lambda x_3\right] - m_S\left[\Lambda x_3 - \ln(1-\Lambda x_3)\right] - m_S \ln K_1 \\ &- m_S\left[\ln i\kappa_0 R_0 + \ln(1-\Lambda x_3 - \pi\Lambda/2\kappa_0) - \Lambda x_3\right] - m_S\left[\Lambda x_3 - \ln(1-\Lambda x_3) - \pi\Lambda/2\kappa_0\right] - m_S \ln K_2 \\ &- \tilde{m}_S\left[\ln(1-\Lambda x_3) - \Lambda x_3\right] - \tilde{m}_S\left[\Lambda x_3 - \ln(1-\Lambda x_3)\right] - \tilde{m}_S \ln K_3 \\ = &-2m_S \ln i\kappa_0 R_0 - m_S(\ln K_1 + \ln K_2) - \tilde{m}_S \ln K_3 \end{aligned} \tag{B.40b}$$

We emphasise that the coupling coefficient κ_0 here is a constant of the geometry, such that it is clear that the overall entropic Hamiltonian in hyperbolic space is therefore conserved and functionally equivalent to the entropic Hamiltonian of a double helix, as seen in Eqs.B.19b and B.19c. The terms associated with the constants of integration $\{K_n\}$ can be assumed essentially independent of the actual geometric structure and are simply a background consideration.

Overall Entropic Field

Summing the 3 components (Eqs.B.37l, B.38m and B.39g) forming the entropic potential field as defined in Euclidean space:

$$V_S(x) = -\frac{e^{i\kappa_0 x_3}}{1-\Lambda x_3}\left(\frac{m_S K_1}{x_1} - i\frac{m_S K_2}{x_2}\right) - \frac{\tilde{m}_S K_3 e^{\Lambda x_3}}{R_3(1-\Lambda x_3)} \tag{B.41}$$

Which we re-write as follows (and from symmetry assuming $K_1 = K_2 = K_0$):

$$V_S(x) = \frac{im_S K_0 e^{i\kappa_0 x_3}}{1-\Lambda x_3}\left(\frac{x_1 + ix_2}{x_1 x_2}\right) - \frac{\tilde{m}_S K_3 e^{\Lambda x_3}}{R_3(1-\Lambda x_3)} \tag{B.42}$$

It is interesting to note what we might consider to be a holomorphic inverse-square law at the heart of this entropic potential field. Here we see that as the trajectory of the logarithmic double spiral progresses with increasing x_3 then the transverse γ_1 and γ_2 components of the entropic field both increase in strength as the radius reduces. For the case where Λ=0 (the double-helix case), then using Eqs.B.1a, B.1b, the entropic field simplifies to:

$$\begin{aligned} V_S(x) &= i\left(\frac{x_1 + ix_2}{x_1 x_2}\right) K_0 m_S e^{i\kappa_0 x_3} - \frac{\tilde{m}_S K_3}{R_3} \\ &= -\frac{e^{i\kappa_0 x_3}}{x_1} K_0 m_S + i\frac{e^{i\kappa_0 x_3}}{x_2} K_0 m_S - \frac{\tilde{m}_S K_3}{R_3} = -\frac{2m_S K_0}{R_0} - \frac{\tilde{m}_S K_3}{R_3} = -\frac{2m_S K_0}{R_0} \end{aligned} \tag{B.43}$$

In this case, the entropic field reduces to the term $-2m_S K_0 / R_0$ (since we can also assume $R_3 \to \infty$) which is uniform and constant such that in the force calculations (differentials) it simply drops out and does not play a direct role: that is, for a double-helix geometry where there is no entropic field, we can equivalently assume V_S=0. The strength of this entropic field is controlled by the factor K_0, which at this stage remains undefined. However, given that the twin-armed logarithmic spiral structure of a galaxy appears to be controlled by the entropy of the super massive black hole at its centre, we can assume that the associated entropic field (of strength controlled by the magnitude of K_0) is therefore considerably stronger than the accompanying gravitational field.

For the logarithmic double spiral the entropic field V_S of Eq.B.42 has been derived using the entropic Euler-Lagrange equation based upon the associated entropic Lagrangian L_S. Since the equations developed in this section obey the Euler-Lagrange equation (B.35), our analysis therefore rigorously demonstrates that the logarithmic double-spiral is a trajectory consistent with the associated entropic Hamiltonian representing a conserved quantity. Our analysis also shows that the generalised mathematical framework (based upon Lagrangian and Hamiltonian equations of state) that we have developed to describe an entropic system, therefore also exhibits an entropic force F_S and entropic mass with associated accelerating trajectory equations that together form an entropic analogue to Newton's kinematic second law of motion.

Concluding Remarks

We have demonstrated that the holomorphic trajectory equations for the double-helix are equivalent to a particle (or a pair of orthogonal plane-waves) travelling in free space in the absence of any fields; also that a logarithmic double spiral is equivalent to a particle travelling in an inverse-square-law potential field. In analysing the trajectory equations, we have shown both how the sum of kinetic entropy (T_S) and entropic potential (V_S) of the trajectories is conserved (as required) and also how the entropic momentum p is conserved in the absence of an entropic field.

When an entropic potential field V_S is present (for the doubled-armed logarithmic spiral, Eq.B.42) , we have demonstrated how the entropic momentum p of the trajectory changes according to the entropic analogue to Newton's 2nd Law of Motion (that is, according to the appropriate Euler-Lagrange equation of variational calculus as defined in hyperbolic space q). Since the entropic Lagrangian L_S for the double-armed logarithmic spiral is associated with an exertion integral X that is at an extremum (or, at the least, is stationary) δX=0, this proves that such a structure (along with a double-helix) is a most-likely (maximum entropy, MaxEnt) configuration, which therefore represents a new explanation for its general ubiquity seen in the natural world.

In performing this analysis, we have also defined entropic analogues to the Hamiltonian (a conserved quantity) and the Lagrangian (which defines the trajectory equations of state). Overall, we find we have created a complete entropic analogue (based in hyperbolic q space) to the conventional energy-based kinematic (mechanical) equations of motion in Euclidean x space.

Annex: holomorphism demonstration

A holomorphic function obeys the Cauchy-Riemann relationships as outlined by Courant & Hilbert [1]. In particular, the entropic functions discussed here obey the hyperbolic Cauchy-Riemann relationships, of which Maxwell's equations are an example. Thus, in order to demonstrate their holomorphism, we simply need to demonstrate that the functions discussed here obey the appropriate Cauchy-Riemann equations.

Consider a 2D holomorphic function, $F = U(x,y) + i\,V(x,y)$ dependent on only two (real) variables x,y and defined as the complex vector formed by the real functions U and V: then the Cauchy-Riemann equations are simply given in 2D by:

$$\frac{\partial U}{\partial x} = \frac{\partial V}{\partial y} \quad \text{and} \quad \frac{\partial V}{\partial x} = -\frac{\partial U}{\partial y} \tag{B.44}$$

In four dimensions, for the hyperbolic Cauchy-Riemann relationships, the 4D holomorphic function $F = U(x_0, x_1, x_2, x_3) + i\,V(x_0, x_1, x_2, x_3)$ must obey equations that are entirely equivalent to the Maxwell's equations:

$$\nabla \times U = \frac{\partial V}{\partial x_0} \quad \text{and} \quad \nabla \times V = -\frac{\partial U}{\partial x_0} \tag{B.45}$$

These Eqs.B.45 can be expressed together and compactly in the geometric algebra as:

$$\nabla F = 0 \tag{B.46}$$

where Eq.B46 can be expressed in the geometrical algebra following Denker [2] (see Appendix A):

$$\nabla F = \begin{matrix} +\nabla^0 U^1\gamma_1 & +\nabla^1 U^1\gamma_1 & -\nabla^2 U^1\gamma_0\gamma_1\gamma_2 & +\nabla^3 U^1\gamma_0\gamma_3\gamma_1 \\ +\nabla^0 U^2\gamma_2 & +\nabla^1 U^2\gamma_0\gamma_1\gamma_2 & +\nabla^2 U^2\gamma_0 & -\nabla^3 U^2\gamma_0\gamma_2\gamma_3 \\ +\nabla^0 U^3\gamma_3 & -\nabla^1 U^3\gamma_0\gamma_3\gamma_1 & +\nabla^2 U^3\gamma_0\gamma_2\gamma_3 & +\nabla^3 U^3\gamma_0 \\ -\nabla^0 V^1\gamma_0\gamma_2\gamma_3 & -\nabla^1 V^1\gamma_1\gamma_2\gamma_3 & -\nabla^2 V^1\gamma_3 & +\nabla^3 V^1\gamma_2 \\ -\nabla^0 V^2\gamma_0\gamma_3\gamma_1 & +\nabla^1 V^2\gamma_3 & -\nabla^2 V^2\gamma_1\gamma_2\gamma_3 & -\nabla^3 V^2\gamma_1 \\ -\nabla^0 V^3\gamma_0\gamma_1\gamma_2 & -\nabla^1 V^3\gamma_2 & +\nabla^2 V^3\gamma_1 & -\nabla^3 V^3\gamma_1\gamma_2\gamma_3 \end{matrix} = 0 \tag{B.47}$$

Holomorphism of a Double Helix

We want to test the holomorphism of the complex-vector $F=U+iV$, where the trajectory functions for a double helix are given by:

$$U = \gamma_1 R_0 \cos\kappa_0 x_3 + \gamma_2 R_0 \sin\kappa_0 x_3 \tag{B.48a}$$

and:

$$V = \gamma_1 R_0 \sin\kappa_0 x_3 - \gamma_2 R_0 \cos\kappa_0 x_3 \tag{B.48b}$$

We notice that the 4D Cauchy-Riemann relationships also require the time-varying aspect to the entropic structure. As the simplest time-varying function, we therefore multiply F by $\exp(-i\omega_0 t)$, where the angular frequency ω_0 can be assume to

be related to κ_0 by the speed of light, $c=\omega_0/\kappa_0$, so that we also have $cdt\equiv dx_0$. Thus, we have $F=(U+iV)\exp(-i\omega_0 t)$. In which case, the Eqs.48 are straightforwardly modified to:

$$U=\gamma_1 R_0\cos\left(\kappa_0 x_3-\omega_0 t\right)+\gamma_2 R_0\sin\left(\kappa_0 x_3-\omega_0 t\right) \quad \text{(B.49a)}$$

and:

$$V=\gamma_1 R_0\sin\left(\kappa_0 x_3-\omega_0 t\right)-\gamma_2 R_0\cos\left(\kappa_0 x_3-\omega_0 t\right) \quad \text{(B.49b)}$$

The Eqs.B49 continue to represent a transverse structure, with $U^3=V^3=0$, so that we have:

$$U^1=R_0\cos\left(\kappa_0 x_3-\omega_0 t\right) \text{ and } U^2=R_0\sin\left(\kappa_0 x_3-\omega_0 t\right) \quad \text{(B.50a)}$$

$$V^1=R_0\sin\left(\kappa_0 x_3-\omega_0 t\right) \text{ and } V^2=-R_0\cos\left(\kappa_0 x_3-\omega_0 t\right) \quad \text{(B.50b)}$$

and it is also clear that the ∇^1 and ∇^2 terms are also zero (∇^1 is $\partial/\partial x_1$ *etc.*), so that Eq.B.47 reduces to the sets of equations:

$$(\nabla^0 U^1-\nabla^3 V^2)\gamma_1+(\nabla^0 U^2+\nabla^3 V^1)\gamma_2=0 \quad \text{(B.51a)}$$

$$(\nabla^3 U^1-\nabla^0 V^2)\gamma_0\gamma_3\gamma_1-(\nabla^3 U^2+\nabla^0 V^1)\gamma_0\gamma_2\gamma_3=0 \quad \text{(B.51b)}$$

and therefore $\nabla^0 U^1=\nabla^3 V^2$ and $\nabla^0 U^2=-\nabla^3 V^1$; as well as $\nabla^3 U^1=\nabla^0 V^2$ and $\nabla^3 U^2=-\nabla^0 V^1$. That is:

$$dU^1/cdt=dV^2/dx_3=\kappa_0 R_0\sin\left(\kappa_0 x_3-\omega_0 t\right) \quad \text{(B.52a)}$$

$$dU^2/cdt=-dV^1/dx_3=-\kappa_0 R_0\cos\left(\kappa_0 x_3-\omega_0 t\right) \quad \text{(B.52b)}$$

and

$$dV^2/cdt=dU^1/dx_3=-\kappa_0 R_0\sin\left(\kappa_0 x_3-\omega_0 t\right) \quad \text{(B.52c)}$$

$$dV^1/cdt=-dU^2/dx_3=-\kappa_0 R_0\cos\left(\kappa_0 x_3-\omega_0 t\right) \quad \text{(B.52d)}$$

The fact that the Eqs.B.52 are indeed all correctly satisfied, and comprise all the non-zero elements of $\nabla F=0$ from Eq.B47, means that the Cauchy-Riemann relations (that is, the analogue to Maxwell's equations) are all satisfied (since the zero elements of $\nabla F=0$ are, by default, also satisfied) so that a double helix (as described by the Eqs.B.48) is therefore a holomorphic structure.

Holomorphism of a Logarithmic Double Spiral

We want to test the holomorphism of a logarithmic double spiral structure, where the radius is now varying exponentially with x_3. The trajectories for the logarithmic double spiral are now given by:

$$U=\gamma_1 R_0 e^{-\Lambda x_3}\cos\kappa_0 x_3+\gamma_2 R_0 e^{-\Lambda x_3}\sin\kappa_0 x_3 \quad \text{(B.53a)}$$

and:

$$V=\gamma_1 R_0 e^{-\Lambda x_3}\sin\kappa_0 x_3-\gamma_2 R_0 e^{-\Lambda x_3}\cos\kappa_0 x_3 \quad \text{(B.53b)}$$

Taking the complex-vector form $F=U+iV$, it is clear that taken together, we can therefore also express the complex-vector as $F\equiv\gamma_1 R_0 e^{-\Lambda x_3}e^{i\kappa_0 x_3}-i\gamma_2 R_0 e^{-\Lambda x_3}e^{i\kappa_0 x_3}$. It is worth noting here that this alternative form for F cannot be directly used to check the Cauchy-Riemann relations, even though it appears to conform to a potentially alternative version of the identity

F=U+iV. However, the Cauchy-Riemann relations only hold for purely real functions U and V; whilst the functions of this alternative form for F are clearly complex in form, in which case they do not form a well-formed set by which to test the Cauchy-Riemann relations. Again, we require an appropriate time-varying component, which we achieve by multiplying F by $\exp(-i\omega_0 t)$ in the same way as was performed for the double helix. However, we also need to additionally impose an analogous 'conjugate' temporal element into each of the exponential terms (that is, in addition to the temporal aspect within the trigonometric expressions) so as to achieve the appropriate balance in the Cauchy-Riemann equations. The equivalent angular frequency for this additional temporal aspect in the exponentials is taken as $c\Lambda$. Together, this therefore allows us to re-express the Eqs.53, using purely real functions, as:

$$U=\gamma_1 R_0 e^{-\Lambda x_3+\Lambda ct}\cos(\kappa_0 x_3-\omega_0 t)+\gamma_2 R_0 e^{-\Lambda x_3+\Lambda ct}\sin(\kappa_0 x_3-\omega_0 t) \tag{B.54a}$$

and:

$$V=\gamma_1 R_0 e^{-\Lambda x_3+\Lambda ct}\sin(\kappa_0 x_3-\omega_0 t)-\gamma_2 R_0 e^{-\Lambda x_3+\Lambda ct}\cos(\kappa_0 x_3-\omega_0 t) \tag{B.54b}$$

These Eqs.B.54 continue to represent a transverse structure, with U^3=V^3=0, such that:

$$U^1=R_0 e^{-\Lambda x_3+\Lambda ct}\cos(\kappa_0 x_3-\omega_0 t) \text{ and } U^2=R_0 e^{-\Lambda x_3+\Lambda ct}\sin(\kappa_0 x_3-\omega_0 t) \tag{B.55a}$$

$$V^1=R_0 e^{-\Lambda x_3+\Lambda ct}\sin(\kappa_0 x_3-\omega_0 t) \text{ and } V^2=-R_0 e^{-\Lambda x_3+\Lambda ct}\cos(\kappa_0 x_3-\omega_0 t) \tag{B.55b}$$

Again, it is also clear that the ∇^1 and ∇^2 terms are also zero (∇^1 is $\partial/\partial x_1$ *etc.*), so that Eq.B.47 again reduces to the same sets of equations:

$$(\nabla^0 U^1-\nabla^3 V^2)\gamma_1+(\nabla^0 U^2+\nabla^3 V^1)\gamma_2=0 \tag{B.56a}$$

$$(\nabla^3 U^1-\nabla^0 V^2)\gamma_0\gamma_3\gamma_1-(\nabla^3 U^2+\nabla^0 V^1)\gamma_0\gamma_2\gamma_3=0 \tag{B.56b}$$

and therefore $\nabla^0 U^1=\nabla^3 V^2$ and $\nabla^0 U^2=-\nabla^3 V^1$; as well as $\nabla^3 U^1=\nabla^0 V^2$ and $\nabla^3 U^2=-\nabla^0 V^1$. That is:

$$dU^1/cdt=dV^2/dx_3=\kappa_0 R_0 e^{-\Lambda x_3+\Lambda ct}\sin(\kappa_0 x_3-\omega_0 t)+\Lambda R_0 e^{-\Lambda x_3+\Lambda ct}\cos(\kappa_0 x_3-\omega_0 t) \tag{B.57a}$$

$$dU^2/cdt=-dV^1/dx_3=-\kappa_0 R_0 e^{-\Lambda x_3+\Lambda ct}\cos(\kappa_0 x_3-\omega_0 t)+\Lambda R_0 e^{-\Lambda x_3+\Lambda ct}\sin(\kappa_0 x_3-\omega_0 t) \tag{B.57b}$$

and

$$dV^2/cdt=dU^1/dx_3=-\kappa_0 R_0 e^{-\Lambda x_3+\Lambda ct}\sin(\kappa_0 x_3-\omega_0 t)-\Lambda R_0 e^{-\Lambda x_3+\Lambda ct}\cos(\kappa_0 x_3-\omega_0 t) \tag{B.57c}$$

$$dV^1/cdt=-dU^2/dx_3=-\kappa_0 R_0 e^{-\Lambda x_3+\Lambda ct}\cos(\kappa_0 x_3-\omega_0 t)+\Lambda R_0 e^{-\Lambda x_3+\Lambda ct}\sin(\kappa_0 x_3-\omega_0 t) \tag{B.57d}$$

Thus, we can see that the Eqs.B.57 are indeed all correctly satisfied; all of which again which represent the non-zero elements of $\nabla F=0$ of Eq.B.47. Therefore the Cauchy-Riemann relations are all satisfied and the logarithmic double spiral is indeed holomorphic.

References

[1] R.Courant & D.Hilbert, *Methods of Mathematical Physics* (Interscience - Wiley, New York, 1962) vol.II p.178

[2] John S. Denker, *Electromagnetism using Geometric Algebra versus Components*, 2008 (https://www.av8n.com/physics/maxwell-ga.pdf; downloaded 26th June 2018)

Appendix C

Appendix C: Euler-Lagrange Equations applied to the Principle of Least Exertion

Abstract

The Euler-Lagrange equations are expressed in hyperbolic space for the entropic cases considered, and the entropic analogue of the principle of stationary action is called the ***exertion***. We confirm that the Euler-Lagrange equations are valid for both the double-helix and the logarithmic double spiral cases.

Exertion

The Euler-Lagrange equations are:

$$\frac{d}{dx_3}\frac{\partial L_S}{\partial q'_n} - \frac{\partial L_S}{\partial q_n} = 0 \tag{C.1}$$

where the prime symbol describes differentiation with respect to x_3 (classically the time dimension), L_S is the appropriate Lagrangian, and (q_n', q_n) are the independent variables of the system.

Such a physical system implies the *principle of stationary action*, which here we can write as:

$$\delta X = \delta\left(\int L_S\, dx_3\right) = 0 \tag{C.2}$$

where X is the integral of the Lagrangian with respect to x_3 (classically known as the *action*).

We show that our definition of the "Entropic Lagrangian" L_S (Eqs.B.14,B.40a) conforms to Eq.C.1 where now L_S is defined in the hyperbolic coordinate system q_n, and x_3 (here the analogue of time) is the index along the geometrical stucture being considered. Recall that the treatment depends on a formal contour integration *with respect to time* in 4-space (see Appendix A), so that it is already a holographic treatment with the 4-space information represented in 3-space: we will also treat the double-helix and the double-armed logarithmic spiral holographically in the sense of using the structural symmetries to further reduce the dimensionality, so that the x_3 direction is treated special (and analogous to time).

Then in our treatment of the *entropic* Lagrangian $L_S(q,q',x_3)$, we will call the analogue of the classical action (X, Eq.C.2), the ***exertion***.

Double Helix

We consider first the relatively straightforward case of the Double Helix. In Appendix B, we have derived the associated entropic Lagrangian, where the entropic potential is zero (V_S=0), as being:

$$L_S\left(q,q',x_3\right) = \sum_{n=1}^{3} q'_n p_n - H_S = 3m_S - \left(T_S + V_S\right) = 3m_S + \sum_{n=1}^{3} m_S \ln q'_n \tag{C.3}$$

As a reminder, the entropic Hamiltonian H_S for a double helix is simply given by the kinetic entropy (KE) term T_S (again, remembering that the entropic potential is zero, V_S=0), since the situation is analogous to that of a free particle in the absence of any fields:

$$H_S = T_S = -\sum_{n=1}^{3} m_S \ln q_n' \tag{C.4}$$

From Appendix B, we have already defined the hyperbolic coordinates appropriate for an entropic double helical geometry as:

$$q_1 = i\kappa R x_3 \tag{C.5a}$$

$$q_2 = iR\left(\kappa x_3 - \tfrac{\pi}{2}\right) \tag{C.5b}$$

$$q_3 \equiv x_3 \tag{C.5c}$$

The associated differential (entropic velocity) terms for a double helix are therefore:

$$q_1' = i\kappa R \tag{C.6a}$$

$$q_2' = i\kappa R \tag{C.6b}$$

$$q_3' = 1 \tag{C.6c}$$

We now consider the three spatial directions in turn.

q_1-coordinate

The Euler-Lagrange equation for the q_1 coordinate direction is given by:

$$\frac{d}{dx_3}\frac{\partial L_S}{\partial q_1'} - \frac{\partial L_S}{\partial q_1} = 0 \tag{C.7}$$

Considering the first term of Eq.C.7 and using (C.3) along with $m_S = i\kappa k_B$, we have:

$$\frac{\partial L_S}{\partial q_1'} = \frac{m_S}{q_1'} = \frac{m_S}{i\kappa R} = \frac{k_B}{R} = \text{constant} \tag{C.8}$$

In which case, the differential of (C.8) with respect to x_3 is simply zero:

$$\frac{d}{dx_3}\frac{\partial L_S}{\partial q_1'} = \frac{d}{dx_3}\left(\frac{k_B}{R}\right) = 0 \tag{C.9}$$

Again, considering the second term of the Euler-Lagrange equation, we note that according to equation (C.3) there is no direct dependence on the actual hyperbolic coordinates q_n; i.e. the entropic potential field is zero, V_S=0. In which case, we can simply write:

$$\frac{\partial L_S}{\partial q_1} = 0 \tag{C.10}$$

From Eqs. C.9, C.10, Eq. C.7 is satisfied, and hence:

$$(\delta X)_{q_1} = \delta\left(\int L_S \, dx_3\right)_{q_1} = 0 \tag{C.11}$$

q_2-coordinate

The Euler-Lagrange equation for the q_2 coordinate direction is given by:

$$\frac{d}{dx_3}\frac{\partial L_S}{\partial q_2'} - \frac{\partial L_S}{\partial q_2} = 0 \tag{C.12}$$

Again, considering the first term of the Euler-Lagrange equation, and using (C.3) along with $m_S = i\kappa k_B$, we have:

$$\frac{\partial L_S}{\partial q_2'} = \frac{m_S}{q_2'} = \frac{m_S}{i\kappa R} = \frac{k_B}{R} = \text{constant} \tag{C.13}$$

In which case, the differential of (C.13) with respect to x_3 is simply zero:

$$\frac{d}{dx_3}\frac{\partial L_S}{\partial q_2'} = \frac{d}{dx_3}\left(\frac{k_B}{R}\right) = 0 \tag{C.14}$$

Again, considering the second term of the Euler-Lagrange equation, we note that according to equation (C.3) there is no dependence on the actual hyperbolic coordinates q_n; i.e. the entropic potential field is zero, V_S=0. In which case, we can simply write:

$$\frac{\partial L_S}{\partial q_2} = 0 \tag{C.15}$$

From Eqs. C.14, C.15, Eq. C.12 is satisfied, and hence:

$$(\delta X)_{q_2} = \delta\left(\int L_S \, dx_3\right)_{q_2} = 0 \tag{C.16}$$

q_3-coordinate

The Euler-Lagrange equation for the q_3 coordinate direction is given by:

$$\frac{d}{dx_3}\frac{\partial L_S}{\partial q_3'} - \frac{\partial L_S}{\partial q_3} = 0 \tag{C.17}$$

Considering the first term of the Euler-Lagrange equation and using (C.3), we have:

$$\frac{\partial L_S}{\partial q_3'} = \frac{m_S}{q_3'} = \frac{m_S}{1} = m_S = \text{constant} \tag{C.18}$$

In which case, the differential of (C.18) with respect to x_3 is simply zero:

$$\frac{d}{dx_3}\frac{\partial L_S}{\partial q_3'} = \frac{d}{dx_3}(m_S) = 0 \tag{C.19}$$

Again, considering the second term of the Euler-Lagrange equation, we note that according to equation (C.3) there is no dependence on the actual hyperbolic coordinates q_n; i.e. the entropic potential field is zero, V_S=0. In which case, we can simply write:

$$\frac{\partial L_S}{\partial q_3} = 0 \tag{C.20}$$

From Eqs. C.19, C.20, Eq. C.17 is satisfied, and hence:

$$(\delta X)_{q_3} = \delta\left(\int L_S \, dx_3\right)_{q_3} = 0 \tag{C.21}$$

Summary

Taking the equations (C.11), (C.16) and (C.21) together, we can therefore write:

$$\delta X = (\delta X)_{q_1} + (\delta X)_{q_2} + (\delta X)_{q_3} = 0 \tag{C.22}$$

We have therefore proved that for a double-helix geometry in hyperbolic space, the exertion X is at an extremum or point of inflection. We now consider the more complex case of the double-armed logarithmic spiral.

Double-Armed Logarithmic Spiral

The entropic Lagrangian for the double-armed logarithmic spiral in hyperbolic space is given by (Appendix B Eq.B.40a):

$$L_S\left(q,q',x_3\right)=\sum_{n=1}^{3}q'_n p_n - H_S = 3m_S-\left(T_S+V_S\right)=3m_S+\sum_{n=1}^{3}m_S\ln q'_n - V_S\left(q_n\right) \qquad \text{(C.23)}$$

where the entropic potential terms in the three hyperbolic spatial directions $V_S(q_n)$ are (Eqs. B.37f, B.38f, B.39d):

$$V_S\left(q_1\right)=-m_S\left[\Lambda x_3-\ln\left(1-\Lambda x_3\right)\right]-m_S\ln K_1 \qquad \text{(C.24a)}$$

$$V_S\left(q_2\right)=-m_S\left[\Lambda x_3-\ln\left(1-\Lambda x_3-\pi\Lambda/2\kappa\right)\right]-m_S\ln K_2 \qquad \text{(C.24b)}$$

$$V_S\left(q_3\right)=-m_S\left[\Lambda x_3-\ln\left(1-\Lambda x_3\right)\right]-m_S\ln K_3 \qquad \text{(C.24c)}$$

where the $-m_S\ln(K_n)$ terms (n=1,2,3) represent background constants of integration. As an aside, it's interesting to note that the equations (C.24) for the entropic potential are expressed as functions in hyperbolic space q. They look quite different when expressed in conventional Euclidean space; indeed the equations (C.25) below therefore do not obey the entropic Euler-Lagrange equation (C.1) since they are described in Euclidean space; equation (C.1) being only valid in hyperbolic space.

$$V_S\left(x_1\right)=\frac{-m_S K_1 e^{i\kappa x_3}}{x_1\left(1-\Lambda x_3\right)} \qquad \text{(C.25a)}$$

$$V_S\left(x_2\right)=\frac{i m_S K_2 e^{i\kappa x_3}}{x_2\left(1-\Lambda x_3-\pi\Lambda/2\kappa\right)}\approx\frac{i m_S K_2 e^{i\kappa x_3}}{x_2\left(1-\Lambda x_3\right)} \qquad \text{(C.25b)}$$

$$V_S\left(x_3\right)=\frac{-m_S K_3 e^{\Lambda x_3}}{R_3\left(1-\Lambda x_3\right)} \qquad \text{(C.25c)}$$

Such that summing them together, the overall entropic potential field in Euclidean space is:

$$V_S\left(x\right)=\frac{i m_S K_0 e^{i\kappa x_3}}{1-\Lambda x_3}\left(\frac{x_1+ix_2}{x_1 x_2}\right)-\frac{m_S K_3 e^{\Lambda x_3}}{R_3\left(1-\Lambda x_3\right)} \qquad \text{(C.26)}$$

where we assume $K_1= K_2= K_0$, and R_3 represents the appropriate radial metric for the x_3 direction. However, Eqs.C.25 and C.26 are not used in this Appendix C, since being in Euclidean space, they do not obey the Euler-Lagrange equations which are only valid in hyperbolic space.

From Appendix B (Eqs. B.25), we have already defined the hyperbolic coordinates appropriate for an entropic double-armed logarithmic spiral geometry as:

$$q_1=iR_0e^{-\Lambda x_3}\kappa x_3 \qquad \text{(C.27a)}$$

$$q_2=iR_0e^{-\Lambda x_3}\left(\kappa x_3-\frac{\pi}{2}\right) \qquad \text{(C.27b)}$$

$$q_3 = x_3 e^{-\Lambda x_3} \tag{C.27c}$$

The associated differential (entropic velocity) terms for a double-armed logarithmic spiral are:

$$q_1' = iR_0 e^{-\Lambda x_3}\kappa\left(1-\Lambda x_3\right) \tag{C.28a}$$

$$q_2' = iR_0 e^{-\Lambda x_3}\kappa\left(1-\Lambda x_3-\frac{\pi\Lambda}{2\kappa}\right) \tag{C.28b}$$

$$q_3' = e^{-\Lambda x_3}\left(1-\Lambda x_3\right) \tag{C.28c}$$

We now consider the three spatial directions in turn.

q_1-coordinate

The Euler-Lagrange equation is given by:

$$\frac{d}{dx_3}\frac{\partial L_S}{\partial q_1'}-\frac{\partial L_S}{\partial q_1}=0 \tag{C.29}$$

The Lagrangian of relevance (ignoring the constant terms) and using Eq.C.24a is given by:

$$L_S\left(q_1,q_1',x_3\right)=-T_S\left(q_1'\right)-V_S\left(q_1\right)=m_S\ln q_1'+m_S\left[\Lambda x_3-\ln\left(1-\Lambda x_3\right)\right] \tag{C.30}$$

Considering the first term of the Euler-Lagrange equation, and using (C.28a) we can write:

$$\frac{\partial L_S}{\partial q_1'}=\frac{m_S}{q_1'}=\frac{m_S}{i\kappa R_0}\frac{e^{\Lambda x_3}}{\left(1-\Lambda x_3\right)} \tag{C.31}$$

Differentiating (C.31) with respect to x_3, and remembering $m_S = i\kappa k_B$, we have:

$$\frac{d}{dx_3}\frac{\partial L_S}{\partial q_1'}=\frac{m_S}{i\kappa R_0}\frac{d}{dx_3}\left(\frac{e^{\Lambda x_3}}{1-\Lambda x_3}\right)=\frac{k_B}{R_0}\frac{\Lambda\left(2-\Lambda x_3\right)}{\left(1-\Lambda x_3\right)^2}e^{\Lambda x_3} \tag{C.32}$$

Considering the second term of Eq.C29 and using equations (C.30) & (C.28a), we can write:

$$\begin{aligned}\frac{\partial L_S}{\partial q_1}&=-\frac{\partial V_S\left(q_1\right)}{\partial q_1}=-\frac{\partial V_S\left(q_1\right)}{\partial x_3}\frac{\partial x_3}{\partial q_1}=-\frac{1}{q_1'}\frac{\partial V_S\left(q_1\right)}{\partial x_3}=\frac{1}{q_1'}\frac{\partial L_S\left(q_1\right)}{\partial x_3}\\&=\frac{e^{\Lambda x_3}}{iR_0\kappa\left(1-\Lambda x_3\right)}m_S\left(\Lambda+\frac{\Lambda}{1-\Lambda x_3}\right)=\frac{k_B}{R_0}\frac{\Lambda\left(2-\Lambda x_3\right)}{\left(1-\Lambda x_3\right)^2}e^{\Lambda x_3}\end{aligned} \tag{C.33}$$

From Eqs.C.32,C.33, Eq.C.29 is satisfied, and hence:

$$\left(\delta X\right)_{q_1}=\delta\left(\int L_S\,dx_3\right)_{q_1}=0 \tag{C.34}$$

q_2-coordinate

The Euler-Lagrange equation is given by:

$$\frac{d}{dx_3}\frac{\partial L_S}{\partial q_2'}-\frac{\partial L_S}{\partial q_2}=0 \tag{C.35}$$

The Lagrangian of relevance (ignoring the constant terms) is (from Eq.C.24b):

$$L_S\left(q_2, q_2', x_3\right) = -T_S\left(q_2'\right) - V_S\left(q_2\right) = m_S \ln q_2' + m_S\left[\Lambda x_3 - \ln\left(1 - \Lambda x_3 - \pi\Lambda/2\kappa\right)\right] \quad \text{(C.36)}$$

Considering the first term of the Euler-Lagrange equation, and using (C.28b) we can write:

$$\frac{\partial L_S}{\partial q_2'} = \frac{m_S}{q_2'} = \frac{m_S}{i\kappa R_0}\frac{e^{\Lambda x_3}}{\left(1 - \Lambda x_3 - \pi\Lambda/2\kappa\right)} \quad \text{(C.37)}$$

Differentiating (C.37) with respect to x_3, and remembering $m_S = i\kappa k_B$, we have:

$$\frac{d}{dx_3}\frac{\partial L_S}{\partial q_2'} = \frac{m_S}{i\kappa R_0}\frac{d}{dx_3}\left(\frac{e^{\Lambda x_3}}{1 - \Lambda x_3 - \pi\Lambda/2\kappa}\right) = \frac{k_B}{R_0}\frac{\Lambda\left(2 - \Lambda x_3 - \pi\Lambda/2\kappa\right)}{\left(1 - \Lambda x_3 - \pi\Lambda/2\kappa\right)^2}e^{\Lambda x_3} \quad \text{(C.38)}$$

Considering the second term of Eq.C.35 and using equations (C.36) & (C.28b), we can write:

$$\begin{aligned}\frac{\partial L_S}{\partial q_2} &= -\frac{\partial V_S\left(q_2\right)}{\partial q_2} = -\frac{\partial V_S\left(q_2\right)}{\partial x_3}\frac{\partial x_3}{\partial q_2} = -\frac{1}{q_2'}\frac{\partial V_S\left(q_2\right)}{\partial x_3} = \frac{1}{q_2'}\frac{\partial L_S\left(q_2\right)}{\partial x_3} \\ &= \frac{e^{\Lambda x_3}}{iR_0\kappa\left(1 - \Lambda x_3 - \pi\Lambda/2\kappa\right)}m_S\left(\Lambda + \frac{\Lambda}{1 - \Lambda x_3 - \pi\Lambda/2\kappa}\right) = \frac{k_B}{R_0}\frac{\Lambda\left(2 - \Lambda x_3 - \pi\Lambda/2\kappa\right)}{\left(1 - \Lambda x_3 - \pi\Lambda/2\kappa\right)^2}e^{\Lambda x_3}\end{aligned} \quad \text{(C.39)}$$

From Eqs.C.38,C.39, Eq.C.35 is satisfied, and hence:

$$\left(\delta X\right)_{q_2} = \delta\left(\int L_S\, dx_3\right)_{q_2} = 0 \quad \text{(C.40)}$$

q_3-coordinate

The Euler-Lagrange equation is given by:

$$\frac{d}{dx_3}\frac{\partial L_S}{\partial q_3'} - \frac{\partial L_S}{\partial q_3} = 0 \quad \text{(C.41)}$$

The Lagrangian of relevance (ignoring the constant terms) is (from Eq.C.24c):

$$L_S\left(q_3, q_3', x_3\right) = -T_S\left(q_3'\right) - V_S\left(q_3\right) = m_S \ln q_3' + m_S\left[\Lambda x_3 - \ln\left(1 - \Lambda x_3\right)\right] \quad \text{(C.42)}$$

Considering the first term of the Euler-Lagrange equation, and using (C.28c) we can write:

$$\frac{\partial L_S}{\partial q_3'} = \frac{m_S}{q_3'} = \frac{m_S e^{\Lambda x_3}}{\left(1 - \Lambda x_3\right)} \quad \text{(C.43)}$$

Differentiating (C.43) with respect to x_3, we have:

$$\frac{d}{dx_3}\frac{\partial L_S}{\partial q_3'} = m_S\frac{d}{dx_3}\left(\frac{e^{\Lambda x_3}}{1 - \Lambda x_3}\right) = m_S 2\Lambda\frac{\left(1 - \frac{1}{2}\Lambda x_3\right)}{\left(1 - \Lambda x_3\right)^2}e^{\Lambda x_3} \quad \text{(C.44)}$$

Considering the second term of the Euler-Lagrange equation and using equations (C.42) & (C.28c), we can write:

$$\frac{\partial L_S}{\partial q_3} = -\frac{\partial V_S(q_3)}{\partial q_3} = -\frac{\partial V_S(q_3)}{\partial x_3}\frac{\partial x_3}{\partial q_3} = -\frac{1}{q_3'}\frac{\partial V_S(q_3)}{\partial x_3} = \frac{1}{q_3'}\frac{\partial L_S(q_3)}{\partial x_3}$$
$$= \frac{e^{\Lambda x_3}}{(1-\Lambda x_3)} m_S \left(\Lambda + \frac{\Lambda}{1-\Lambda x_3}\right) = m_S 2\Lambda \frac{\left(1-\tfrac{1}{2}\Lambda x_3\right)}{(1-\Lambda x_3)^2} e^{\Lambda x_3} \quad \text{(C.45)}$$

From Eqs.C.44,C.45, Eq.C.41 is satisfied, and hence:

$$(\delta X)_{q_3} = \delta\left(\int L_S\, dx_3\right)_{q_3} = 0 \quad \text{(C.46)}$$

Summary

From Eqs.C.34,C.40, C.46 we have:

$$\delta X = (\delta X)_{q_1} + (\delta X)_{q_2} + (\delta X)_{q_3} = 0 \quad \text{(C.47)}$$

We have therefore proved that for a double-armed logarithmic spiral geometry in hyperbolic space, the exertion X is at an extremum or point of inflection. Note that the double-helix is only a special case (with $\Lambda = 0$) of the logarithmic double-spiral.

Appendix D

Appendix D: Geometric Entropy Analysis of Double Helical or Spiral Structures: DNA & the Milky Way Galaxy

Abstract

The geometrical entropy of the double-helix, and then (as a generalisation) of the logarithmic double-spiral is calculated. DNA is the exemplar of the first case, and the Milky Way (arguably a double-armed galaxy) of the second. It is not surprising that the same entropic treatment applies from the nano-scale to the cosmic, since entropy is intrinsically scaleless.

Calculation of the Entropy of a Double Helix

We first determine the geometric entropy associated with a double-helical structure. We have already obtained (in Appendix B, Eq.B.19b) the generalised local entropic Hamiltonian H_S for an axial structure (such as a double helix, for which q_3'=1) where in the absence of any entropic potential field ($V_S = 0$, so that $H_S = T_S$), and bearing in mind that in this case the q_n' (n=1,2) are pure imaginary (see Eqs.B.5):

$$H_S = -m_S\left(\ln q_1' + \ln q_2'\right) \tag{D.1}$$

where we underline that a double-helix is associated with 2 orthogonal plane waves or eigenvectors.

Since the Hamiltonian is defined as an integral it is permissible without loss of generality to include any convenient constant "background" term. In this case, we choose to add the constant quantity $m_S \ln \kappa_0 R_0$ (clearly independent of the entropic momentum, position and velocity quantities: p, q and q') and clearly only a function of the two unvarying geometric parameters of the double helix (i.e. radius R_0, and pitch λ_0) for each of the two eigenvectors. See Appendix B: m_S is given by Eq.B.6a, and κ_0 and R_0 are given by Eqs.B.1. Then, from Eq.B.19a, the resulting entropic Hamiltonian for the double helix is:

$$H_S = m_S\left(2\ln\kappa_0 R_0 - \ln q' - \ln q_2'\right) = m_S\left(2\ln\kappa_0 R_0 + \ln\frac{p_1}{m_S} + \ln\frac{p_2}{m_S}\right) \tag{D.2a}$$

For the case of the DNA molecule, the radius of the double helix transverse to its axis is R_0, that is $R_1 = R_2 = R_0$. From Eqs.B.4,5,6a we have:

$$p_n / (k_B/R_0) = \kappa_0 x_n / x_n' \qquad n = 1,2 \tag{D.2b}$$

for the double helical geometry, using $x_1 = R_0 \exp\left(i\kappa_0 x_3\right)$ and $x_2 = -iR_0 \exp\left(i\kappa_0 x_3\right)$, such that $\kappa_0 x_n / x_n' = -i$. Additionally, given $m_S \equiv i\kappa_0 k_B$ (and Euler's identity) we can write the entropic Hamiltonian for a double helix:

$$H_S = i\kappa_0 k_B\,(2\ln\kappa_0 R_0 + 2\ln(1/i\kappa_0 R_0)\,) = \pi\kappa_0 k_B \tag{D.3a}$$

That means that the associated entropic Langrangian for a double helix is therefore simply given by:

$$L_S = 3m_S - H_S = 3m_S - \pi\kappa_0 k_B \tag{D.3b}$$

The geometric entropy of the double helix is given as $S = \sqrt{1+\kappa_0^2 R_0^2}\int H_S\, dx_3$, thus the entropy for the DNA double helix is given by the following integral:

$$S = \pi \kappa_0 k_B \sqrt{1+\kappa_0^2 R_0^2} \int_0^L dx_3 = \pi \kappa_0 \sqrt{1+\kappa_0^2 R_0^2}\, L k_B \tag{D.4}$$

This simple expression (D.4) for S is well behaved and also physically plausible, being directly proportional to the length of the double helix (DNA), dependent upon the physical extent of the structure (that is, its radius R_0, as well as its length L) and also independent of the absolute location of the double helix. It is also a straightforward function of the radius R_0 and pitch λ_0 (or wavenumber κ_0) of the double helix, and is also appropriately dimensioned by Boltzmann's constant k_B.

Calculation of B- and P-DNA Geometric Entropies

Under a constant tension of K=45 pN, the DNA sample was overwound by 1,200 turns, during which the B-DNA underwent a molecular extension in length of Δx = 0.7 µm. From the simple geometry, the energy expended during this procedure is $W=K\times\Delta x$ =31.5 aJ. Assuming all things remain equal, for the situation where the DNA sample is overwound by approximately 4,800 turns (as indicated in ref.[28], and in inset of its Fig.2) for an extension in length of Δx = 2.8 µm, we therefore expect the extension energy expended to be of the order of W=126 aJ. The additional energy expended due to the applied torque of 34 pN.nm and 4,800 turns is 34 pN.nm × 2π× 4,800=1025 aJ, so that the total expended mechanical energy is 1151 aJ.

The change in geometric entropy for the B- and P-DNA geometries is calculated as follows. A length of B-DNA, consisting of 14,800 base-pairs (14.8 kbp), with 10.5 bp/turn, has N=14.8x10^3/10.5=1410 turns. With the pitch of standard B-DNA measured to be λ = 3.32 nm, the length of the 14.8-kbp B-DNA molecule is $L=N\times\lambda$=4.681 µm. The radius of B-DNA is taken to be R=1.0nm. Eq.D.4 is used to calculate the geometric entropy of B-DNA as $S_{\text{B-DNA}}$=59,572k_B.

When the same length of B-DNA is supercoiled (overwound) it adopts the P-DNA over-extended, high-helicity form. In this case, P-DNA now features 2.6 bp per turn, so for the same number of 14.8 kbp, the total number of turns is now given by N=14.8×10^3/2.6=5,692 turns. The pitch of P-DNA is given as λ=1.28 nm, so its over-extended total length L is now given as $L=N\times\lambda$=7.286 µm; that is, the P-DNA is approximately 55.6% longer than the B-DNA structure, and the change in overall length is Δx=7.286 µm – 4.681 µm = 2.605 µm (in reasonable agreement with the *ceteris paribus* assumption above of Δx=2.8µm.) We assume the radius of P-DNA after undergoing a 55.6% extension is close to R=0.6 nm. Then from Eq.D.4 the geometric entropy of the 14.8-kbp P-DNA molecule is $S_{\text{P-DNA}}$=349,480k_B.

The change in geometric entropy of the 14.8-kbp DNA molecule is therefore ΔS=289,908k_B. At an ambient temperature of 23°C (that is, 296 K) and with k_B=1.38×10^{-23} m^2.kg/s^2.K, this represents an energy change of 1184 aJ.

Geometric Interpretation for Structural Entropy of Double Helix

The equation (D.4) also offers an interesting geometric interpretation for the entropy associated with a double-helical structure. It can be straightforwardly rewritten as:

$$S = 2\pi^2 \frac{L\sqrt{\lambda_0^2 + (2\pi R_0)^2}}{\lambda_0^2} k_B = \frac{2\pi^2}{\sin\alpha} \frac{A}{\lambda_0^2} k_B \tag{D.5}$$

where $A \equiv 2N(2\pi R_0 \lambda_0/2) = 2\pi R_0 L$ is the surface area of the curved part of the cylinder encapsulating a section of the double-helical structure of length $L = N\lambda_0$ (ignoring the area of the two end pieces of the cylinder, each of area πR_0^2 , since $R_0 \ll L$ for DNA). The helical angle α is as indicated in the figure below, such that $\tan\alpha = 2\pi R_0/\lambda_0$.

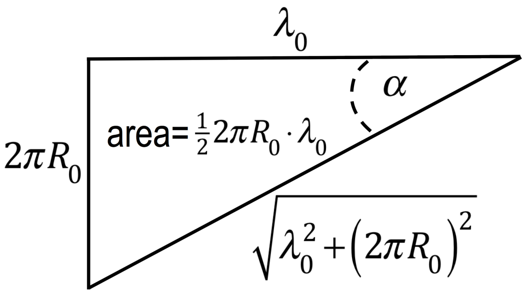

Figure D.1: Length of one period of an unwrapped helix to show its geometric properties.

The geometric entropy (D.5) is directly proportional to the surface area A of the double-helical structure under consideration, thus conforming to the holographic principle. The entropy is also inversely proportional to the square of the wavelength associated with the structure with the constant of proportionality given by $2\pi^2/\sin\alpha$. It is clear that, for $\kappa_0 R_0 \gg 1$, we can approximate (D.4) to:

$$S = \pi\kappa_0^2 R_0 L k_B \tag{D.6a}$$

And, if the length $L/2$ is the same as the radius (that is, a "spherical" structure), this becomes:

$$S = 2\pi\kappa_0^2 R_0^2 k_B \tag{D.6b}$$

Calculation of the Geometric Entropy of a Double Spiral Galaxy

Consider two logarithmic spirals with trajectories described by the following equations:

$$l_1 = r_{BH} e^{-\Lambda x_3} \cos\kappa x_3 \gamma_1 + r_{BH} e^{-\Lambda x_3} \sin\kappa x_3 \gamma_2 \tag{D.7a}$$

$$l_2 = r_{BH} e^{-\Lambda x_3} \sin\kappa x_3 \gamma_1 - r_{BH} e^{-\Lambda x_3} \cos\kappa x_3 \gamma_2 \tag{D.7b}$$

where the logarithmically varying radius has parameter Λ. We will model the Milky Way galaxy with this formalism.

In this case, the Schwarzschild radius of the black hole at the centre of the galaxy is given by r_{BH}, while the radius of the galaxy is given by R_G at $x_3 = -L/2$ (where the subscript 'G' is used to indicate the parameter's value at the galaxy's outer edge, while subscript 'BH' is generally used to indicate the value at the black hole), while Λ describes the logarithmic change in the radius of the galactic arm with azimuthal angle. The azimuthal angle is given by $\theta = \kappa x_3$, where $\kappa = 2\pi/\lambda$ is the galactic wavenumber with λ being the wavelength scale in the γ_3 direction (see Fig.D.2).

The two galaxy arms together form a composite system described by $\Sigma = l_1 + il_2$:

$$\Sigma = r_{BH} e^{i(\kappa + i\Lambda)x_3} \gamma_1 - i r_{BH} e^{i(\kappa + i\Lambda)x_3} \gamma_2 \tag{D.8}$$

which is holomorphic. From Appendix B (Eq.B.24c) the radius R varies exponentially with x_3, and for the entropic Hamiltonian H_S to be constant in hyperbolic space (as is required since the equations of state obey the Euler-Lagrange equations in hyperbolic space; see Appendix C), the product $\kappa R = \kappa_G R_G = \kappa_{BH} r_{BH}$ must be independent of the γ_3 direction, that is:

$$R = r_{BH} \exp(-\Lambda x_3) \qquad \text{by definition} \tag{D.9a}$$

$$\kappa = \kappa_{BH} \exp(\Lambda x_3) \qquad \text{for constant } \kappa R \text{, and hence} \tag{D.9b}$$

$$\lambda = \lambda_{BH} \exp(-\Lambda x_3) \qquad \text{since } \kappa \equiv 2\pi/\lambda \tag{D.9c}$$

The entropic Hamiltonian in general is given, as is conventional, by $H_S = T_S + V_S$, where both entropic Hamiltonian H_S and the entropic potential terms V_S are defined in hyperbolic space. From Appendix B, §B.3, Eq.B.19c, as well as Appendix C, Eqs.C.24 and Eqs.C.28 we therefore have:

$$H_S = -2m_S \ln(i\kappa_G R_G), \quad \text{with} \quad m_S \equiv i\kappa_G k_B \tag{D.10a}$$

As for the double helix (see Eqs.D.2-D.3) we add the constant $2m_S\ln(\kappa_G R_G)$ to the entropic Hamiltonian of Eq.D.10a, so that in the subsequent analysis we can use the more insightful quantity:

$$H_S = \pi\kappa_G k_B \tag{D.10b}$$

Note that in hyperbolic space the double helix is therefore isomorphic to the double-armed logarithmic spiral.

The hyperbolic co-ordinates for the logarithmic spiral then become:

$$q_1 = ir_{BH}e^{-\Lambda x_3}\kappa_{BH}e^{\Lambda x_3}x_3 = i\kappa_{BH}r_{BH}x_3 = i\kappa_G R_G x_3 \tag{D.11a}$$

$$q_2 = ir_{BH}e^{-\Lambda x_3}\left(\kappa_{BH}e^{\Lambda x_3}x_3 - \frac{\pi}{2}\right) = ir_{BH}\left(\kappa_{BH}x_3 - \frac{\pi}{2}e^{-\Lambda x_3}\right) \approx i\kappa_{BH}r_{BH}x_3 = i\kappa_G R_G x_3 \tag{D.11b}$$

We can make the close approximation in Eq.D.11b since $\kappa_{BH} >> \Lambda\exp(-\Lambda x_3)\pi/2$. For completeness, we include here the q_3 hyperbolic co-ordinate:

$$q_3 = x_3 \tag{D.11c}$$

In the limit as $\Lambda = 0$, the logarithmic double-spiral tends to the simple double-helix. It is also clear that the constant κR of the logarithmic spiral trajectory represents the transverse hyperbolic velocities:

$$q_1' = i\kappa_G R_G = i\kappa R \tag{D.12a}$$

$$q_2' = ir_{BH}\left(\kappa_{BH} + \Lambda\frac{\pi}{2}e^{-\Lambda x_3}\right) \approx i\kappa R \tag{D.12b}$$

where the approximation $q_1' = q_2'$ is valid if $\Lambda\exp(-\Lambda x_3)\pi/2 << \kappa_{BH}$.

Having determined the entropic Hamiltonian H_S and confirmed that it is a conserved quantity, we now calculate the overall entropy S associated with the double spiral by integrating the Hamiltonian over the length of the holomorphic trajectory, $S = \int H_S\, dl$. Figure D.2 indicates the geometry of performing such a line integral along the logarithmic spiral trajectory.

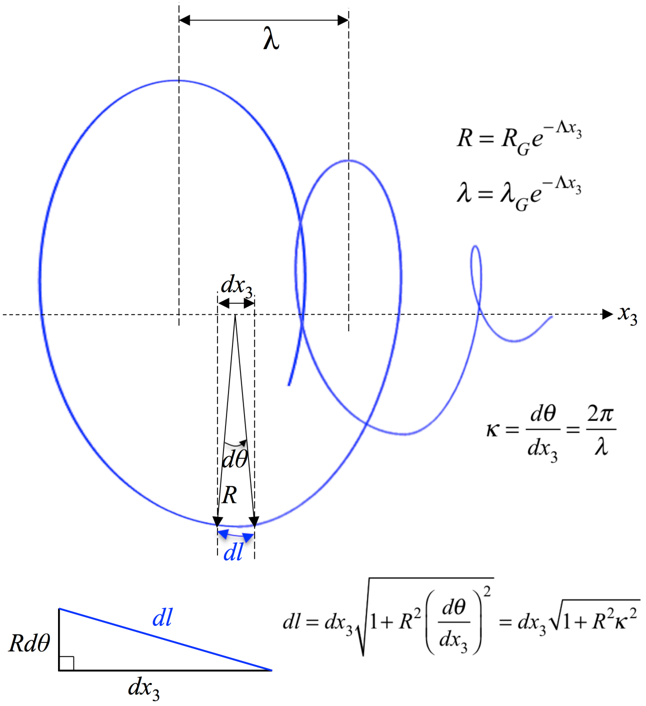

Figure D.2: Geometry of the pathlength along a logarithmic spiral trajectory.

In particular, we note the Pythagorean relationship between the infinitesimal distance along the pathlength dl and the infinitesimal distance dx_3 along the x_3-axis, such that we can assume $dl=dx_3\sqrt{(1+(\kappa R)^2)}$, where $\kappa R = \kappa_G R_G = \kappa_{BH} r_{BH}$=const. Then, using Eq.D.10b:

$$S = \int_0^L H_S \, dl = \pi \kappa_G k_B L \sqrt{1+\kappa_G^2 R_G^2} \tag{D.13a}$$

It is also clear, that for $\kappa_G R_G \gg 1$, we can approximate (D.13a) to:

$$S = \pi \kappa_G^2 R_G L k_B \tag{D.13b}$$

This is identical to the form of Eq.D.6a. In order to calculate the geometric entropy of the galaxy, we therefore also require the parameter λ_G, the appropriate galactic wavelength scale. Although the value for the wavelength (pitch) λ_0 was obvious for the calculation of the DNA geometric entropy, the physical determination and interpretation of the galactic wavelength λ_G is not so obvious.

Calculating the Galactic Wavelength λ_G

It has been noted that the overall entropy of the universe is dominated by the contributions of the entropies due to the presence of super-massive black holes. In other words, at a galactic level, the entropy of a galaxy will be dominated by the entropy of the super massive black hole located at its centre. We therefore approximate the geometric galactic entropy by the Bekenstein-Hawking entropy of the central super-massive black hole.

We first need to calculate a value for the logarithmic radial parameter Λ. We assume that the galactic logarithmic radius reduces from the radius R_G at the galaxy periphery down to the Schwarzschild radius r_{BH} of the central super-massive black hole, given by:

$$r_{BH} = \frac{2GM_{BH}}{c^2} \tag{D.14}$$

where G is the gravitational constant, M_{BH} is the mass of the black hole, and c is the speed of light. Assuming the radial descent occurs over the semi-thickness length $L/2$ of the galaxy we must have

$$r_{BH} = R_G \exp\left(-\Lambda L/2\right) \tag{D.15a}$$

In which case, we have:

$$\Lambda = \frac{2}{L} \ln \frac{R_G}{r_{BH}} \tag{D.15b}$$

For the specific example of the Milky Way, $\kappa >> \Lambda$, $R\kappa >> 1$, and $R_G \kappa_G >> 1$. From Eq.D.13b and the Bekenstein-Hawking expression for the black hole entropy S_{BH} we obtain:

$$S_{BH} = \frac{1}{4} \frac{4\pi r_{BH}^2}{l_P^2} k_B = \frac{4\pi^3 R_G L}{\lambda_G^2} k_B \tag{D.15c}$$

where $l_P = \sqrt{\hbar G / c^3}$ is the Planck length, and hence that the appropriate galactic wavelength λ_G at the galaxy periphery is:

$$\lambda_G = \sqrt{\frac{4\pi^2 \hbar G R_G L}{r_{BH}^2 c^3}} = 2\pi \frac{l_P}{r_{BH}} \sqrt{R_G L} \tag{D.16}$$

If we define the ratio of the galactic radius with its thickness with the symbol Ω, i.e. $\Omega \equiv R_G/L$, then substituting into equation (D.16) indicates that Ω is constant:

$$\Omega = \frac{R_G}{L} = 4\pi^2 \left(\frac{l_P}{\lambda_G} \frac{R_G}{r_{BH}} \right)^2 = 4\pi^2 \frac{e^{-2\Lambda L}}{e^{-2\Lambda L}} = 4\pi^2 \tag{D.17}$$
